# The cross-domain functional organization of posterior lateral temporal cortex: insights from ALE meta-analyses of 7 cognitive domains spanning 12,000 participants

**DOI:** 10.1093/cercor/bhac394

**Published:** 2022-10-20

**Authors:** Victoria J Hodgson, Matthew A Lambon Ralph, Rebecca L Jackson

**Affiliations:** MRC Cognition and Brain Sciences Unit, University of Cambridge, 15 Chaucer Road, Cambridge, CB2 7EF, United Kingdom; MRC Cognition and Brain Sciences Unit, University of Cambridge, 15 Chaucer Road, Cambridge, CB2 7EF, United Kingdom; MRC Cognition and Brain Sciences Unit, University of Cambridge, 15 Chaucer Road, Cambridge, CB2 7EF, United Kingdom; Department of Psychology & York Biomedical Research Institute, University of York, Heslington, York, YO10 5DD, United Kingdom

**Keywords:** language, meta-analysis, semantic cognition, social cognition, temporal lobe

## Abstract

The posterior lateral temporal cortex is implicated in many verbal, nonverbal, and social cognitive domains and processes. Yet without directly comparing these disparate domains, the region’s organization remains unclear; do distinct processes engage discrete subregions, or could different domains engage shared neural correlates and processes? Here, using activation likelihood estimation meta-analyses, the bilateral posterior lateral temporal cortex subregions engaged in 7 domains were directly compared. These domains comprised semantics, semantic control, phonology, biological motion, face processing, theory of mind, and representation of tools. Although phonology and biological motion were predominantly associated with distinct regions, other domains implicated overlapping areas, perhaps due to shared underlying processes. Theory of mind recruited regions implicated in semantic representation, tools engaged semantic control areas, and faces engaged subregions for biological motion and theory of mind. This cross-domain approach provides insight into how posterior lateral temporal cortex is organized and why.

## Introduction

The functional organization of posterior lateral temporal cortex (pLTC) is a mystery. The region, or subregions within, are implicated in a wide array of disparate domains, from aspects of language processing through to understanding intentional action, raising questions about its organization. Is this brain area composed of many tessellated, discrete subregions each subserving different functional domains, or is pLTC responsible for a smaller number of core cognitive processes that underpin a range of domains? Focusing within a single cognitive domain, researchers may miss the clues as to a particular region’s function that could be provided by the region’s involvement in other domains. Here, we take a broader view. In the present study, a series of activation likelihood estimation (ALE) meta-analyses were used to compare and contrast activation systematically across 7 different cognitive domains commonly associated with bilateral pLTC. This large, cross-domain meta-analysis investigated how multiple different cognitive processes are supported by pLTC and determined the principles underlying the functional organization of this area.

The functions ascribed to the pLTC (here defined as including the posterior half of the lateral temporal lobe, excluding the basal surface, and extending dorsally into the temporo-parietal junction, TPJ) are numerous and diverse, and vary in scope. The pLTC has been implicated extensively in semantic cognition, including both the representation of multimodal conceptual knowledge and the controlled, flexible, and goal-oriented use of that knowledge (Jefferies 2013; Lambon Ralph et al. 2017). Following the recognition that posterior brain damage can result in a semantic control deficit (Jefferies and Lambon Ralph 2006; Noonan et al. 2009; Gardner et al. 2012; Rogers et al. 2015), meta-analyses have highlighted a particular role for the left posterior middle temporal gyrus (pMTG) in semantic control, defined as the flexible access and manipulation of meaningful information to focus on task-relevant aspects of a concept (Noonan et al. 2013; Jackson 2020). However, pLTC regions are also implicated in semantic representation, both as a whole and in the representation of specific semantic categories, such as knowledge about tools (defined as manipulable man-made objects) in the left temporo-occipital-parietal junction (Chao et al. 1999a; Boronat et al. 2005; Creem-Regehr and Lee 2005; Lewis 2006; Ebisch et al. 2007; Binder et al. 2009; Xu et al. 2019; Lesourd et al. 2021), actions more generally (Tranel et al. 2003; Campanella et al. 2010), faces in the superior temporal sulcus (STS), typically with stronger activation in the right (Kanwisher et al. 1997; Haxby et al. 1999; Hoffman and Haxby 2000; O’Toole et al. 2002; Bernstein and Yovel 2015; Pitcher and Ungerleider 2021) and bodies or body parts. Indeed, a portion of the pMTG known as the extrastriate body area, particularly in the right hemisphere, is proposed to be specialized for the visual detection of bodies and body parts (Downing et al. 2006; Spiridon et al. 2006; Peelen and Downing 2007; Taylor et al. 2007), and bilateral posterior superior temporal sulcus (pSTS) is implicated in the low level detection of biological motion across a broad range of real and point-light display stimuli (Bonda et al. 1996; Allison et al. 2000), including bodies, faces, expressions, and mouth and eye movements (Perrett et al. 1992; Bonda et al. 1996; Phillips et al. 1997; Puce et al. 1998; Haxby et al. 2000; Hoffman and Haxby 2000; Beauchamp et al. 2003; Hooker et al. 2003; Calder and Young 2005).

Beyond semantics, the pLTC is implicated in phonological processing, particularly in left superior temporal gyrus (STG) and the ventral aspects of inferior parietal cortex, which are included in the scope of the current investigation (Hickok and Poeppel 2004, 2007; Vigneau et al. 2006; Yang and Small 2015). Indeed, damage around the posterior Sylvian fissure can cause conduction aphasia, characterized by impaired repetition, naming difficulties and phonemic paraphasias, despite good comprehension (Damasio and Damasio 1980; Hickok 2000; Graves et al. 2008; Buchsbaum et al. 2011). Regions within the pLTC are also considered important for social cognition, an umbrella term for a collection of processes such as empathy, interpreting intentional actions, and false belief understanding (Allison et al. 2000; Saxe et al. 2004; Saxe 2006). In particular, bilateral TPJ is considered a crucial region for understanding the mental states of others, or “theory of mind,” including classic false belief tasks (Saxe and Kanwisher 2003; Frith and Frith 2006; Saxe 2006).

To date, the regions implicated in these myriad domains have not been systematically compared with one another. As such, it is not clear to what extent overlapping pLTC regions are implicated in different domains; for instance, is the same region of pMTG critical for semantic control, the representation of tools, and the detection of body parts? In part, this is due to the imprecision in anatomical labeling, making it difficult to know whether researchers in disparate areas of cognitive neuroscience are referring to the same, overlapping, or entirely distinct regions when using the same terminology, or indeed, if they are referring to the same regions when using different terminology. The TPJ is a prime example of this; the label “temporo-parietal junction” is ill-defined and does not map precisely to anatomical features (Schurz et al. 2017). As such it is variously used to refer to parts of the pSTG, angular gyrus (AG), and supramarginal gyrus (Binder et al. 2009; Carter and Huettel 2013). It is also likely that these established anatomical labels are too coarse to capture subregions within the posterior temporal cortex. Furthermore, functional involvement and activation patterns do not always respect neat anatomical boundaries.

To combat these difficulties, the present study used a series of ALE meta-analyses, within a pLTC region of interest (ROI), as a tool for direct, statistical comparison across the key domains associated with this region, including semantics, semantic control, phonology, representation of tools, representation of faces, perception of biological motion, and theory of mind. This allowed us to elucidate the functional organization of pLTC by investigating whether different functions recruit the same areas—and as such may rely on common underlying neural processes and computations, about which we can begin to form some hypotheses—or whether they engage distinct, neighboring subregions.

## Materials and methods

### Definition of ROI

**Fig. 1:**
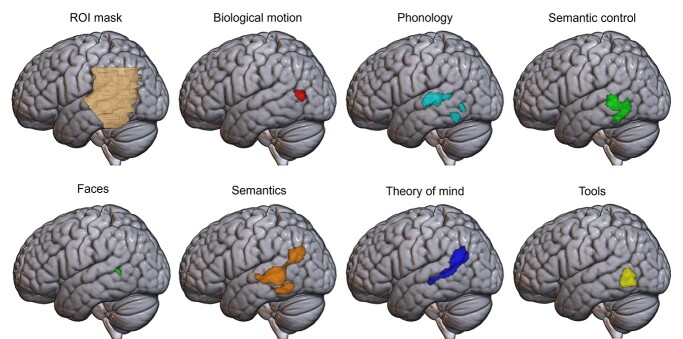
Activation likelihood estimation maps for each of the 7 domains, showing clusters of consistent activation across studies in the left hemisphere, at a voxel-level cluster-forming threshold of *P* < 0.001, and a cluster threshold of *P* < 0.05 (FWE corrected). Top left: the region of interest mask used for all analyses, with only the left hemisphere visible here.

To cover the pLTC, including the ill-defined TPJ, an ROI was constructed consisting of the posterior portion of inferior temporal gyrus (ITG), MTG, and STG along with the inferior portion of parietal cortex. As studies and domains highlighting the role of the “TPJ” may or may not be referring to the posterior lateral temporal cortex proper, a full TPJ region was included, allowing identification of the region implicated in a domain, for comparison with other domains, irrespective of whether the region identified is strictly temporal or parietal, though the parietal lobe was not the focus of the present study. The basal temporal lobe was excluded as its complex organization has already received extensive discussion and is not the focus of the present investigation (Kanwisher et al. 1997; Epstein et al. 1999; Chao et al. 1999b; Cohen et al. 2000; Haxby et al. 2000; McCandliss et al. 2003; Peelen and Downing 2005; Kanwisher 2017). The ROI was constructed using the automated anatomical labeling (AAL) anatomical atlas (Tzourio-Mazoyer et al. 2002) taken from MRIcron (http://www.nitrc.org.projects/mricron) in Montreal Neurological Institute (MNI) space and defined entirely using neuroanatomical landmarks and boundaries, to avoid making reference to existing functional regions. Including the lateral temporal lobe meant bounding the ROI posteriorly by the boundary with the occipital lobe and ventrally by the boundary between the inferior temporal gyri and the fusiform gyrus, as defined by the AAL atlas. To focus on the posterior aspects of the temporal cortex, the ROI was limited anteriorly by a line beginning at the anterior edge of Heschl’s gyrus on the lateral surface (*y* = −19, *z* = 6 in MNI space) and progressing approximately perpendicular to the Sylvian fissure, thus including a similar amount of each gyrus. In addition, an inclusive approach was taken to ensure coverage of the ill-defined TPJ region, by including the inferior parietal lobe, defined as the area ventral to the intraparietal sulcus. Thus, the ROI was bounded dorsally by a horizontal plane at the level of the intraparietal sulcus on the lateral surface (*z* = 42 in MNI space), to allow for the inclusion of the inferior parietal lobe but the exclusion of the superior parietal lobe. The left-hemisphere view of the ROI is displayed in Fig. 1 and the right hemisphere view in Fig. 3.

### Meta-analyses

Independent ALE analyses, restricted to the ROI, were completed for each of the 7 domains, before comparison between domains. This method, which has successfully been used in prior comparisons elsewhere in the brain (Visser et al. 2009; Rice et al. 2015), was chosen over masking a whole-brain ALE map. A masked whole-brain result may result in cluster “fragments” at the edges of the ROI, and would have lower power due to multiple comparison correction for regions that are outside the remit of the study (Müller et al. 2018a). To promote identification of all relevant clusters an inclusive ROI was utilized, including inferior parietal regions, as well as the core pLTC.

The cognitive domains included were based on our assessment of the pLTC literature. In addition, we checked that no further cognitive domains implicating lateral posterior temporal cortex, with existing meta-analyses or significant bodies of literature appropriate for the purposes of ALE meta-analysis, e.g. a minimum of 17 studies for inclusion (Eickhoff et al. 2012; Müller et al. 2018a), were accidentally missed. The online tool NeuroSynth (Yarkoni et al. 2011; https://neurosynth.org/) was used to review the first 200 topic words most associated with 4 seed regions spread across the ROI (peaks at ±56, −46, −4; ±46, −48, and −16). Most of these terms were synonymous or highly related to the domains assessed and none highlighted additional cognitive domains with sufficient data for inclusion.

Studies for each domain were sourced from one or more existing meta-analyses, in order to ensure that accepted, peer-reviewed operationalizations of each domain were used and directly compared with each other for the first time, and to maximize the breadth of the domains that were included. For each study matching the inclusion criteria, each peak was assessed for overlap with the ROI and only peaks within the ROI were included.

#### Inclusion and exclusion criteria

Analyses included only peer-reviewed articles written in English, describing task-based functional magnetic resonance imaging and PET studies that reported peak coordinates of a univariate contrast in standard (MNI or Talairach) space across the whole-brain and focused on a young healthy adult sample (mean age below 40 years old). Contrasts were excluded if they focused on patients, clinical trials, or individual differences (e.g. age, gender, and native language). Within each included article, wherever multiple task contrasts were reported for the same participant sample, all the peak activation coordinates were analyzed as a single contrast, to avoid artificially inflating the consistency between studies following the recommendation from Müller et al. (2018a). No contrasts were included in >1 domain. In some cases where a contrast included content that may overlap with a second domain (e.g. the presentation of face stimuli in contrasts assessing theory of mind), such contrasts were either excluded a priori or, if this resulted in a large reduction in sample size, the analysis was performed with and without their removal to maximize power while aiding the interpretation of the between-domain comparison. Details on the contrasts included for each domain can be found in Supplementary Tables 1–7.

### Semantics

To identify the pLTC regions recruited for semantic cognition across categories and processes, a general semantic contrast was included. Studies were sourced from a recent meta-analysis by Jackson (2020), which included 272 verbal and nonverbal contrasts published between 1992 and 2019 that specifically compared a semantic condition with a non-semantic (or less semantic) condition. These studies could include individual semantic categories, such as faces, compared with a baseline but did not contrast different categories of concepts. All contrasts involving tools, a total of 2 contrasts, were removed to allow uncontaminated comparison with the tool domain. This resulted in the inclusion of 580 foci across 204 experiments after restriction to the pLTC ROI.

### Semantic control

A further 126 contrasts assessing semantic control, in particular, were sourced from Jackson (2020). These contrasted high over low semantic demands with both verbal and nonverbal stimuli, using a range of manipulations, including association strength, competitor interference, and homonym ambiguity. These comparisons recruit a subset of the regions responsible for semantic cognition (dissecting semantics into areas responsible for semantic control and semantic representation). None of these contrasts focused on tool stimuli. Within the ROI, there were 104 foci across 41 experiments.

### Phonology

Studies were sourced from a meta-analysis by Hodgson et al. (2021), which identified studies from the Vigneau et al. (2006) and Humphreys and Lambon Ralph (2015) meta-analyses and performed a literature search extending the timespan of the studies included to April 2021. Tasks included both passive listening and active judgments. The studies contrasted either phonological with semantic or orthographic judgments, or phonological with non-phonological stimuli (e.g. visual perception tasks). Contrasts were excluded if the phonological task contained words or other overtly semantic stimuli. From an initial pool of 82 papers published between 1992 and 2021, 207 foci across 64 experiments were included.

### Theory of mind

Studies were sourced from 2 meta-analyses by Molenberghs et al. (2016) and Diveica et al. (2021), resulting in a pool of 147 papers published between 1999 and 2020. Both meta-analyses included both affective and cognitive theory of mind tasks, implicit and explicit task instructions, and varying stimuli (photographs, cartoons, stories, games, videos, and animations). Commonly used tasks included the Reading the Mind in the Eyes task for mental state evaluation (Baron-Cohen et al. 2001), the false belief over false photograph task (Zaitchik 1990) or similar belief over physical reasoning, and social over nonsocial games or animations. For the full dataset, 502 foci across 152 experiments were included. To aid precise interpretation of the regions initially implicated in both theory of mind and face processing, a subset of the dataset was analyzed further, in which contrasts including face stimuli were removed. This reduced dataset included 384 foci across 122 experiments.

### Biological motion

Studies were sourced from a meta-analysis by Grosbras et al. (2012), which included 110 papers published between 1996 and 2010. Four contrasts that did not include full motion, but only static or implied motion, were removed. All contrasts compared biological motion over scrambled motion, nonbiological motion, or static images. The biological motion stimuli included point-light displays and moving body regions such as the hands or face, and most studies involved passive viewing. Any contrasts that included tool manipulation were removed, to eliminate overlap with the tool domain. Trials including manipulation of non-tool items (e.g. grasping a ball or similar object) were included. The full dataset comprised 223 foci across 53 experiments. In addition, a subset of this dataset was analyzed, in which any contrasts with stimuli that included faces—real or animated—were removed; this subset comprised 151 foci across 40 experiments.

### Faces

Studies were sourced from 2 meta-analyses by Müller et al. (2018a) and Eickhoff et al. (2012), yielding a total of 139 papers published between 1992 and 2015. Contrasts included faces over non-face objects (e.g. houses). Therefore, although stimuli could include both emotional and neutral faces, none explicitly assessed the effect of emotion by contrasting emotional over neutral faces. The full dataset included 85 foci from 41 experiments within the pLTC. A subset of this dataset with all contrasts featuring emotion evaluation tasks removed, was analyzed, with a total of 48 foci from 24 experiments, with most remaining studies employing gender evaluation or identity judgment tasks.

### Tools

Contrasts for the tools domain were drawn from 3, partially overlapping meta-analyses by Ishibashi et al. (2016), Humphreys and Lambon Ralph (2015) and Chen et al. (2017), resulting in a total pool of 76 papers published between 1996 and 2013. All contrasts included the presentation of tool or tool-related stimuli over the presentation of non-tool stimuli, including other semantic categories, such as animals or faces, and non-semantic items such as scrambled images. Contrasts typically included photographs of tools over non-tools, but in some cases focused on tool sounds, action verbs and motor imagery specifically related to tools. The small number of experiments with dynamic stimuli were removed to reduce conflation with the biological motion domain. The final dataset included 116 foci from 41 experiments.

#### Activation likelihood estimation

Meta-analyses were performed using ALE in GingerALE version 3.0.2 using the command line (https://brainmap.org/ale/ (Turkeltaub et al. 2002; Eickhoff et al. 2009, 2012, 2017); code included in Supplemental Materials 1). ALE is a meta-analytic technique that maps the statistically significant convergence of activation probabilities between experiments considered to reflect similar processes. This is achieved by modeling all foci for each experiment as Gaussian probability distributions, with the full width at half maximum (FWHM) of each Gaussian being determined by the sample size of the study (i.e. larger samples result in less uncertainty of the peak’s location and a narrower distribution). This results in a modeled activation map for each experiment included in the analysis. The union of these maps is then calculated to produce an ALE score in each voxel, with each ALE score representing the probability of activation being present at that given voxel in a study.

All analyses were performed in MNI space and restricted to the ROI. Where necessary, foci were converted from Talairach space using GingerALE, which uses the Lancaster transform (Lancaster et al. 2007). Only foci that fell within the ROI were included in each contrast. The ROI was used as a mask for all statistical analyses, restricting the possible locations where a peak would be expected to fall by chance, in line with Müller et al. (2018a). This allows assessment of whether the peak coordinates generated across studies are more consistently clustered than would be expected by chance, within the volume of the ROI. ALE scores were thresholded at the voxel level at *P* < 0.001 for cluster-forming. Cluster-level family-wise error (FWE) correction at *P* < 0.05, with 10,000 permutations, was then applied to determine the minimum significant cluster size and remove nonsignificant clusters (Müller et al. 2018a). All peaks are reported in MNI space.

Pairwise comparisons were also performed on the resulting ALE maps, with conjunction and subtraction analyses revealing the distinct and shared areas across each pair of domains. Contrasts are only presented in the main text for pairs of domains with key overlapping activations, with the remainder presented in the Supplementary Materials (Supplementary Figs. 1–16, see online supplementary material for a color version of these figure). The conjunction image in each case is the voxel-wise minimum of the thresholded ALE maps for the 2 domains. For subtraction analyses, the procedure described in Laird et al. (2005) is used, in which all experiments across the 2 domains are pooled and randomized across many iterations to construct a null distribution for the difference in ALE scores, from which a *Z*-value map for the actual observed difference can be calculated. For the present analyses, 10,000 permutations were performed, and a conservative uncorrected threshold of *P* < 0.001 and minimum cluster volume of 20 mm^3^ were applied to extract the clusters.

## Results

Activation peaks for each of the 7 domains across both hemispheres are provided in Table 1. Though the lack of significant activation likelihood for a domain in one hemisphere does not preclude its involvement, there are clear relative differences across hemispheres for many domains, and laterality appears an important organizational factor. As such, and to aid description, the results are presented separately for each hemisphere followed by a consideration of the effect of laterality, though all analyses were conducted in a single step on the bilateral pLTC ROI.

**Table 1:** Activation likelihood estimation across all domains.

Domain	L/R	Region of activation	Peak MNI coordinate		
			*x*	*y*	*z*
Biological motion	R	Posterior MTG	50	−68	0
			52	−62	4
	L	Posterior MTG	−50	−68	8
Faces	R	Posterior STG	52	−50	8
			56	−40	0
			50	−42	8
	R	Posterior MTG	50	−74	2
			48	−72	−8
	L	Posterior STS	−50	−48	6
Phonology	L	Posterior middle STG/STS	−60	−24	4
			−60	−32	4
			−56	−46	8
	R	Posterior middle STS	60	−30	2
	L	Posterior ITG	−50	−54	−16
	L	Posterior MTG/ITG	−50	−60	−4
Semantic control	L	Posterior MTG/ITG/STS	−54	−42	4
			−56	−46	−4
			−46	−48	−18
			−46	−56	−12
Semantics	L	AG, mid-to-posterior STS, posterior MTG	−56	−38	2
			−46	−66	26
			−62	−20	2
	L	Posterior ITG	−48	−54	−14
			−44	−44	−18
	R	Posterior STS	52	−34	0
Theory of mind	L	AG, mid-to-posterior STS	−52	−58	22
			−56	−26	−6
			−56	−38	0
	R	AG	56	−54	26
	R	Posterior MTG	52	−32	−2
Tools	L	Posterior MTG	−50	−66	−6
			−52	−58	2

### Left hemisphere

In the left hemisphere, significant clusters were found for all 7 domains (see Fig. 1).

The semantics domain engaged a large region of the left hemisphere, extending from the edge of the AG, along the posterior MTG/STG/STS, toward the anterior edge of the ROI (the whole brain results reported by Jackson (2020) extend into anterior temporal lobe) and ventrally into posterior ITG. These posterior MTG/STS and ITG regions were also found to be activated consistently across the semantic control assessments, demonstrating the particular role of this region for the controlled use of semantics (consistent with Jackson, 2020), whereas the more dorsal STG regions identified only in the general semantics contrast may reflect more general semantic processes. The phonology domain was associated with consistent recruitment of the mid-to-posterior STG, as well as 2 clusters in posterior ITG overlapping semantics, semantic control and tools. The theory of mind cluster was similar in extent and volume to the semantics cluster, extending from AG along the STS until it reached the anterior edge of the ROI, although lacking involvement of the ventral portion of MTG and ITG areas associated with control. Note that either inferior parietal or posterior temporal regions could be referred to as TPJ when studying theory of mind (Carter and Huettel 2013). A small cluster for faces was found in the pSTS/pMTG, overlapping the semantics and theory of mind domains, but with minimal overlap with phonology and semantic control. Smaller clusters were identified for tools and biological motion in the most posterior portion of the ROI near the temporo-occipital border. Biological motion consistently recruited the most posterior region of any domain, located in the pMTG bordering the middle occipital gyrus, just dorsal to the tools cluster. Tools consistently recruited an area of the middle temporal sulcus near the temporo-occipital junction, inferior to biological motion and overlapping semantic control.

**Fig. 2:**
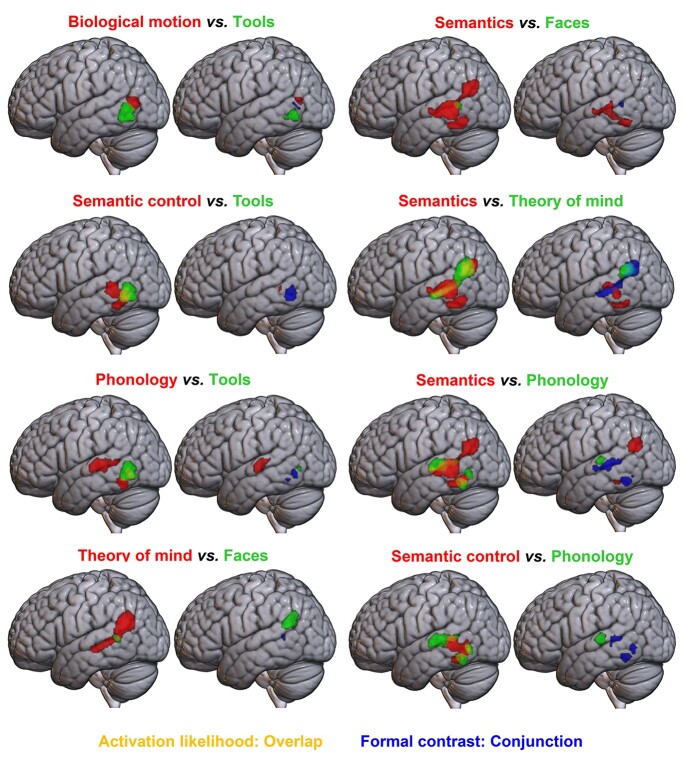
Formal contrast analyses between pairs of domains in the left hemisphere at a voxel-level threshold of *P* < 0.001 (uncorrected). For each pair of images, left: pairwise overlays of ALE maps, showing domain A in red and domain B in green, with overlap in yellow. For each pair of images, right: results of formal contrast and conjunction analyses; domain A > B is shown in red, B > A in green, and their conjunction in blue.

Formal ALE contrast analyses (conjunction and subtraction) were conducted for each pair of domains. The results of key comparisons that further aid understanding of activation likelihood in the left hemisphere are shown in Fig. 2, with peaks given in Table 2. Note that, as the overlapping nature of semantics and semantic control is expected and established (Jackson 2020), the contrasts between semantics and semantic control, and semantics and tools (in the semantic control regions shown to overlap with tools below) do not aid the results interpretation further and can be found in the Supplemental Materials along with full bilateral results of all other conjunction and subtraction analyses between pairs of domains with overlapping voxels (see Supplemental Figs. 1–16, see online supplementary material for a color version of these figures). Similarly, where overlap is very minimal and better explained by other comparisons (for faces vs. semantic control, faces vs. phonology, theory of mind vs. semantics, and theory of mind vs. faces) contrasts may be found in the Supplemental Materials.

**Table 2:** Pairwise contrast analyses in the left hemisphere.

Contrast	Region of activation	Peak MNI coordinate		
		*x*	*y*	*z*
*Subtraction analyses*				
Biological motion > tools	V5/MT	−52	−66	14
		−48	−68	14
Tools > biological motion	ITG	−52	−63	−6
Semantic control > tools	MTG	−54	−46	2
Tools > semantic control	*No peaks*			
Phonology > tools	STG	−57	−22	4
		−57	−29	9
		−60	−16	4
Tools > phonology	ITG	−53	−68	0
Theory of mind > faces	STG	−53	−54	25
		−53	−56	25
Faces > theory of mind	*No peaks*			
Semantics > faces	MTG	−59	−36	−3
		−61	−31	−2
		−62	−36	−8
		−58	−16	0
	Fusiform	−53	−51	−13
		−44	−54	−12
		−48	−58	−14
	Angular gyrus	−46	−68	34
Faces > semantics	*No peaks*			
Semantics > theory of mind	MTG	−62	−44	−2
		−62	−32	5
		−57	−37	5
	ITG	−48	−49	−18
Theory of mind > semantics	STG/AG	−53	−55	22
Semantics > phonology	MTG	−45	−61	24
	ITG/fusiform	−39	−42	−16
		−42	−44	−18
Phonology > semantics	STG	−58	−24	8
Semantic control > phonology	*No peaks*			
Phonology > semantic control	STG	−59	−24	7
*Conjunction analyses*				
Biological motion & tools	MTG/ITG	−52	−66	4
		−52	−64	6
		−50	−68	2
		−50	−62	8
Semantic control & tools	ITG	−50	−58	−8
Phonology & tools	ITG	−50	−60	−4
	ITG	−50	−56	−12
Theory of mind & faces	STS	−52	−48	6
		−50	−50	8
Semantics & faces	STS	−50	−48	6
Semantics & theory of mind	STS/AG	−54	−42	4
Semantics & phonology	STS	−60	−32	4
		−60	−26	2
		−60	−20	2
		−56	−46	8
	ITG	−50	−54	−16
		−48	−60	−10
Semantic control & phonology	STS	−56	−44	6
	ITG	−50	−60	−6
	ITG	−48	−56	−14
		−48	−50	−16
	STS	−58	−36	0

The contrast analyses reveal the possible relations between tools and the other domains. Although the tools cluster is bordering the biological motion result, direct comparison revealed little conjunction between these domains, indicating distinct regions. However, the majority of the tools result overlaps with the dorsal aspects of the semantic control cluster without significant differential involvement, and there are no voxels showing significantly greater involvement in tools over semantic control. The most posterior inferior cluster for phonology overlaps this same region, and contrast analyses show significant conjunction between phonology and semantic control and between phonology and tools here, with only a small cluster for tools over phonology in the associated subtraction analyses. This could reflect a single region for control processes, which may show some domain-specificity, for example for semantic stimuli (including tools) or for language stimuli more generally (see the Discussion for consideration of why this could be the case).

Although the phonology and semantics (and to a lesser extent semantic control) clusters overlap along their edges in the STS, the STG involvement was unique for phonology. The ventral edge of AG and a small region of pITG demonstrated greater involvement for semantics. This suggests relatively distinct regions of pLTC for semantics and phonology, particularly as the semantic domain includes auditory words, so the influence of phonology cannot be entirely excluded. Both phonology and semantic control engaged the pITG. Although the pMTG was identified for semantic control only, this difference did not reach statistical significance.

Direct contrasts revealed a large region of overlap between semantics and theory of mind. Despite this, inferior (semantic control-related) pLTC areas demonstrated greater semantic involvement. Although the more dorsal parietal region is found consistently for both semantics and theory of mind, it has a greater likelihood of activation within the theory of mind domain. Conjunction analyses between faces and semantics and between faces and theory of mind revealed a similar region of conjunction in both cases, and no significant voxels for faces over semantics or theory of mind. Though the faces cluster also shared some overlapping voxels with semantic control and phonology, this overlap was minimal, and so those contrasts are shown in the Supplemental Materials.

### Right hemisphere

**Fig. 3:**
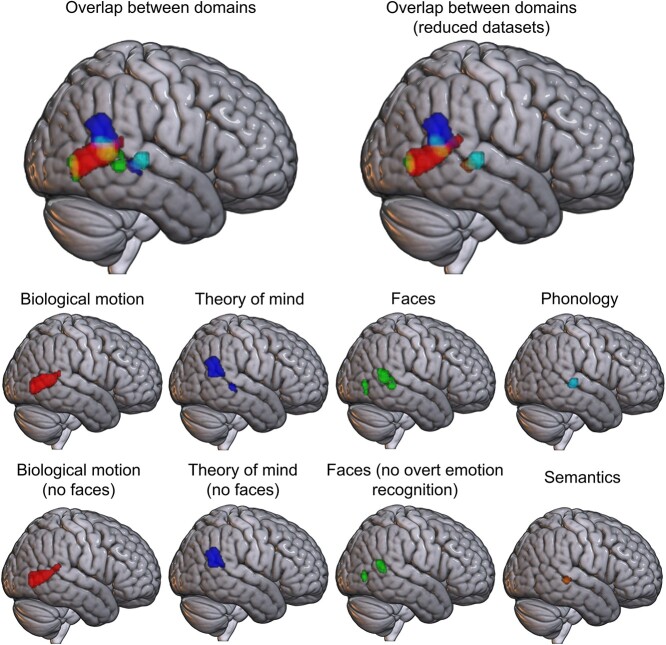
Activation likelihood estimation maps for the 5 domains with significant results in the right hemisphere, at a voxel-level cluster-forming threshold of *P* < 0.001 and a cluster threshold of *P* < 0.05 (FWE corrected). Top: overlap between domains, without (top left) and with (top right) exclusions designed to minimize overlap in the included content. Bottom rows: ALE maps for the 5 domains, plus ALE maps constructed using reduced datasets for 3 of the domains. For domains not shown (semantic control and tools), no significant activation likelihood was found in the right hemisphere.

Significant consistent recruitment of right hemisphere pLTC regions was found for 5 of the 7 domains: faces, theory of mind, biological motion, semantics, and phonology. Further ALE analyses were conducted for subsets of 3 of these domains (theory of mind, biological motion, and faces), to minimize the possible confounding effects of overlap in the content of the included studies (see Fig. 3 and Table 3).

**Table 3:** Activation likelihood estimation across reduced datasets.

Domain	L/R	Region of activation	Peak MNI coordinate		
			*x*	*y*	*z*
Biological motion	R	Posterior STS/MTG/ITG	50	−68	0
			50	−64	2
			60	−42	16
	L	Posterior MTG	−50	−68	8
Faces	R	Posterior STG	54	−56	16
			54	−52	10
	R	Posterior ITG	50	−74	2
Theory of mind	L	TPJ/AG	−52	−58	22
	R	TPJ/AG	56	−52	26
			50	−70	8
	L	STS/MTG	−58	−26	−6

In the STS/MTG, small, overlapping clusters for phonology, theory of mind, and semantics were revealed; for phonology and semantics, this included the entire extent of their activation likelihood in the right hemisphere. Theory of mind, biological motion, and faces all engaged posterior aspects of the pLTC, yet differed on the ventral-dorsal dimension. Theory of mind consistently recruited the AG and posterior STG. Biological motion engaged the posterior aspects of the temporal lobe, including MTG, ITG and to a lesser extent, STG. A small region of posterior MTG/STS was implicated in the faces domain, overlapping with both biological motion and theory of mind, along with a cluster in the most posterior part of the MTG, close to the temporo-occipital border, and which overlapped the posterior edge of the biological motion cluster.

**Fig. 4:**
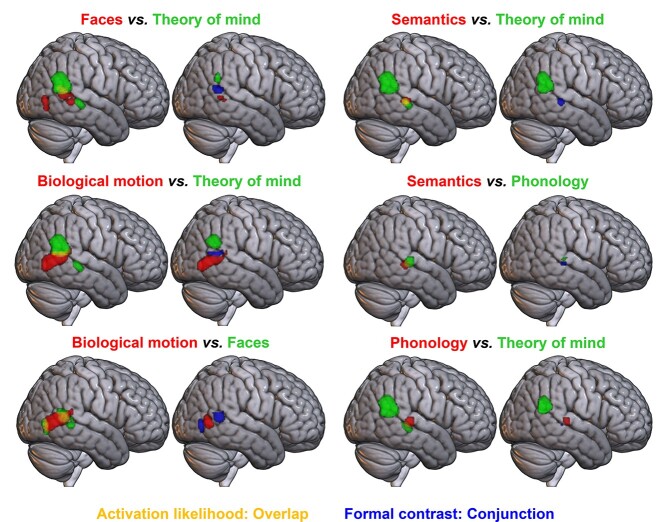
Formal contrast analyses between pairs of domains with overlap in the right hemisphere at a voxel-level threshold of *P* < 0.001 (uncorrected). For each pair of images, left: pairwise overlays of ALE maps, showing domain A in red and domain B in green, with overlap in yellow. For each pair of images, right: results of formal contrast and conjunction analyses; domain A > B is shown in red, B > A in green, and their conjunction in blue.

There were 2 areas of overlapping voxels in the right hemisphere: theory of mind, biological motion, and faces overlapped in the most posterior and dorsal part of the ROI, whereas theory of mind, phonology, and semantics overlapped with one another in the STS/MTG (see Fig. 4 and Table 4). As theory of mind engaged a more dorsal region than biological motion, their overlap was minimal at the edge of each cluster, suggesting distinct, albeit nearby areas are implicated in these domains. However, the more anterior faces cluster was intermediate between theory of mind and biological motion, showing considerable overlap with both, whereas the more posterior faces cluster also overlapped nearly entirely with the biological motion cluster. To assess whether this overlap was the result of the use of face stimuli in some studies within the biological motion and theory of mind domains, reduced datasets excluding face stimuli were considered. Removing face stimuli had little effect; the studies employing face stimuli are insufficient to explain the overlap between theory of mind or biological motion and faces. In addition, the overlap between theory of mind and faces could be hypothesized to be due to the use of emotional recognition tasks in the face domain, a task used to assess both face processing and theory of mind. However, excluding experiments featuring explicit emotion evaluation tasks from the faces domain, did not reduce this overlap. Indeed, although fewer studies resulted in a smaller cluster, it was the regions that overlapped with biological motion and theory of mind that remained. Thus, these potential confounds cannot explain the identified organization of right pLTC, with substantial overlap between faces and both theory of mind and biological motion, even after controlling for these effects.

**Table 4:** Pairwise contrast analyses in the right hemisphere.

Contrast	Region of activation	Peak MNI coordinate		
		*x*	*y*	*z*
*Subtraction analyses*				
Faces > theory of mind	MTG	54	−47	3
		52	−50	6
		55	−45	2
Theory of mind > faces	STG/AG	61	−51	25
		50	−52	28
		54	−52	32
Biological motion > theory of mind	ITG/MTG	52	−61	2
	STG	61	−40	12
Theory of mind > biological motion	TPJ/AG	56	−55	27
Biological motion > faces	MTG	52	−63	3
Faces > biological motion	*No peaks*			
Semantics > theory of mind	*No peaks*			
Theory of mind > semantics	TPJ/AG	56	−52	22
Semantics > phonology	*No peaks*			
Phonology > semantics	STG	59	−30	7
Phonology > theory of mind	STG	57	−26	6
Theory of mind > phonology	TPJ/AG	56	−52	23
*Conjunction analyses*				
Faces & theory of mind	STG	54	−50	10
Biological motion & theory of mind	STG/MTG	54	−52	10
		50	−60	12
Biological motion & faces	STG	52	−50	8
	ITG	50	−74	2
		48	−70	−8
Semantics & theory of mind	STS/MTG	52	−32	0
Semantics & phonology	STS	56	−28	2
Phonology & theory of mind	STS	56	−32	0

The results of pairwise formal contrast analyses between these domains are shown in Fig. 4 (also see Table 4). As the cross-domain overlap could not be explained by any of the factors assessed in the reduced datasets for theory of mind, biological motion, and faces, these formal contrasts employed the full datasets in each of these cases, to maximize power. Formal contrasts between reduced datasets gave similar results and can be found in the Supplemental Materials (Supplemental Figs. 17–19, see online supplementary material for a color version of these figures). Contrast analyses demonstrated significant differences in activation likelihood across the majority of the biological motion and theory of mind clusters, suggesting relatively distinct regions for biological motion, in the most posterior part of the MTG at the border of the occipital lobe, and theory of mind, in the AG. Although overlapping with the faces domain, areas in each of these subregions displayed significantly greater activation likelihood for their associated domain than for faces. Although a small region of posterior MTG was identified for faces over theory of mind, this fell within the biological motion area and there were no significant voxels for faces over biological motion in the subtraction analysis. This provides no support in favor of there being face-specific (i.e. exclusively involved in faces) pLTC subregions, although some areas may show relatively greater responses to face stimuli.

Contrast analyses between phonology, semantics and theory of mind revealed a similar pattern as seen in the left-hemisphere homologue. In the STS/MTG region, semantics and theory of mind showed significant conjunction only. However, both domains showed a small cluster of conjunction with phonology in pSTS, with a region in STG appearing for both phonology over semantics and phonology over theory of mind, indicating that this more dorsal STG region may be recruited for phonology alone in the right hemisphere, as in the left.

## Discussion

**Fig. 5:**
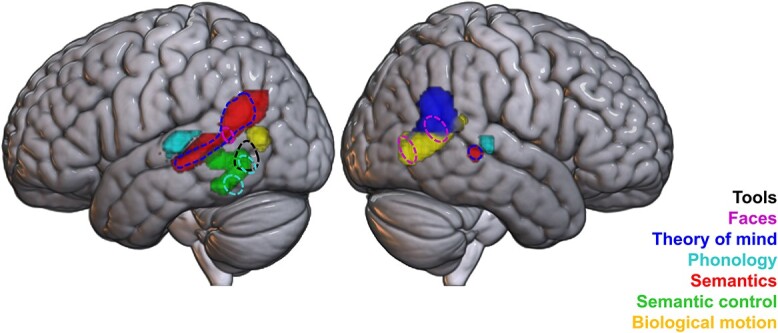
A synthesis of results across the 7 domains in the bilateral posterior lateral temporal cortex ROI. Regions with distinct activation are seen for some domains, e.g. phonology and biological motion, whereas some domains appear subsumed by others, such as tools by semantic control (indicated by dashed lines). To aid interpretation based on the full set of analyses assessing and comparing each domain, this synthesis figure comprises a number of clusters from ALE analyses of a single domain (semantic control, biological motion, theory of mind, and left-hemisphere phonology), as well as the results of subtraction and conjunction analyses between domains (for the phonology and semantics clusters in bilateral STS/STG).

Direct comparison across a series of 7 ALE meta-analyses assessing diverse domains, delineated the functional organization of the pLTC. The resulting structure was neither a single discrete region per domain (as previously associated with the basal temporal lobe; Kanwisher 2017) nor a highly overlapping region suggesting a great deal of shared processing across many domains (as identified in the inferior parietal cortex; [Bibr ref71][Bibr ref71]), but a midpoint on this continuum. Many domains recruited dissociable areas, likely reflecting discrete functional regions. However, a number of domains implicated highly similar subregions and may reflect shared processing. This may be expected as the domains vary in breadth, with a limited number of domains having hierarchical relationships, such as between tools and semantics. However, these domains are typically studied independently and the precise nature of their neural relationships (e.g. tools overlapping with semantic control but not semantic representation, faces overlapping with theory of mind and not semantics) is not apparent without testing. In addition, some broad domains (e.g. theory of mind and semantic representation) were also found to rely on overlapping neural correlates. These findings have been synthesized into a summary diagram (Fig. 5). The remainder of the Discussion considers this organization and its implications, including possible explanations for the reliance of multiple domains on shared regions.

### Left-lateralized domains

#### Semantics and phonology

Both semantics and phonology had a left-hemisphere focus, with some limited right pSTS involvement. These domains were underpinned by dissociable pLTC regions consistent with previous meta-analytic, theoretical, and neuropsychological work (Bates et al. 2003; Hickok and Poeppel 2004, 2007; Rogers et al. 2004; Vigneau et al. 2006; Binder et al. 2009; Friederici 2009, 2011; Noonan et al. 2013; Mesulam et al. 2014; Jackson 2020). A discrete region of bilateral STG was implicated in phonology; this region is associated with processing speech sounds (Binder et al. 2000; Scott et al. 2000) and damage here is linked to conduction aphasia (Damasio and Damasio 1980; Hickok 2000; Buchsbaum et al. 2011) and pure word deafness (Poeppel 2001; Stefanatos et al. 2005). In contrast, a swathe of the left pSTS extending dorsally to the ventral edge of AG, was implicated in general semantic processing (due to its involvement in semantics, yet not semantic control), which overlapped only minimally with phonology; further, in both hemispheres, subtraction analyses indicate that the STG shows significantly greater activation likelihood for phonology than semantics. This indicates broadly separable regions for phonology and semantic representation in the pLTC, consistent with the relative independence of damage to semantic and phonological processes observed within the neuropsychological literature (Hodges and Patterson 1996; Robson et al. 2012). For instance, although damage to the pSTG leads to the combined semantic-phonological impairment seen in Wernicke’s aphasia, lesions in the pMTG result in multimodal semantic impairment without phonological impairment, seen in semantic aphasia (Robson et al. 2012). This dissociation is consistent with sharper cytoarchitectural distinctions between the STG and MTG than between other temporal gyri (Brodmann 1909; Bajada et al. 2017; Jackson et al. 2018). The precise interpretation of the semantic left pSTS region, which is seen in the present analysis for semantics but not phonology, and is on the edge of the posterior temporal semantic control region, requires further investigation. One possibility is that the pSTS region may be involved in the comprehension of sentences (Friederici et al. 2003, 2009) or of narrative gestalt meaning, increasing its involvement as consistent, time-extended meaning builds up, perhaps in conjunction with parietal regions (Yeshurun et al. 2017; Branzi et al. 2020).

#### Semantic control, tools, and phonology

The left pITG/pMTG/pSTS was also associated with semantics, specifically its controlled access and manipulation; no significant activation likelihood for semantics or semantic control was found in the right hemisphere homologue of this region, with the right pSTS semantics cluster lying more anterior to this area, consistent with prior whole brain results (Noonan et al. 2013; Davey et al.^2016^; Jackson 2020). This aligns with neuroimaging studies of semantic control (Jefferies 2013; Noonan et al. 2013; Jackson 2020; Gao et al. 2021; Hodgson et al. 2021), and with the regions typically damaged in semantic aphasia, in which patients have difficulty accessing weaker or subordinate associations, and poor inhibition of strong associations, following damage to this area due to stroke (Jefferies and Lambon Ralph 2006; Corbett et al. 2009; Noonan et al. 2009). A posterior lateral temporal region for semantic control is also consistent with modeling work that indicates a need for control processes to interact with modality-specific spokes, rather than the multimodal anterior temporal lobe (ATL) hub (Jackson et al. 2021). This left pLTC region, comprising areas of pMTG, pITG, and pSTS, may act as an intermediary between the ATL hub and the IFG (Davey et al. 2016), and is well-placed to interact with surrounding modality-specific spokes, such as visual areas in the fusiform gyrus and occipital lobe, auditory areas in the STG, and praxis areas in inferior parietal cortex (Rogers et al. 2004; Lambon Ralph et al. 2017; Jackson 2020).

Two clusters were also found in left pITG for phonology, both of which overlapped with semantic control. Previously demonstrated to respond particularly to hard phonological tasks (Hodgson et al. 2021) and implicated in the extended multiple demand network (Assem et al. 2020), this region may reflect shared control processing for language subdomains or across a broad set of domains. It is unclear whether this constitutes a discrete region, or whether pMTG and pITG demonstrate a graded shift in a preference for the control of meaningful stimuli. The distinction between control and representation processes seems to be a key organizational principle for language regions (Jackson 2020; Hodgson et al. 2021) and the brain more broadly (Duncan 2010; Fedorenko et al. 2013; Camilleri et al. 2018), and as such is a critical factor to consider when delineating the organization of the pLTC. Representation regions engage more dorsal subregions of the left posterior temporal lobe, whereas control processes—which may be domain-general, or specific to language—engage more ventral subregions, including pSTS, pMTG, and pITG (Assem et al. 2020; Jackson 2020; Hodgson et al. 2021).

Notably, 1 of the 2 clusters of significant conjunction between phonology and semantic control in the pITG also overlaps with the tools domain. The pMTG and pITG are both believed to form part of a “common tool-use circuit”—one that also recruits the inferior parietal lobe and premotor areas—and it has previously been proposed that these posterior temporal regions contribute the storage of function knowledge (Reynaud et al. 2016; Lesourd et al. 2021). Indeed, tools are one semantic category amongst many, and the present analyses found no evidence that tools recruit a distinct region in the pLTC beyond those that are implicated in semantics across other categories; however, it is notable that tools engage an area of the pLTC specialized for control, rather than representation, of semantic information. Although differences in the relative engagement of basal temporal areas across categories are hypothesized to relate to low level visual properties of the stimuli (Whatmough et al. 2002; Price et al. 2003; Rogers et al. 2005; Pourtois et al. 2009; Chen and Rogers 2014), differences in the lateral temporal cortex may have alternative explanations related to higher-level multimodal processes. In comparison with other semantic categories, tools may rely more heavily on control processes (Creem-Regehr and Lee 2005). Much of our conceptual understanding of a manipulable tool relies on praxis, which requires the unfolding of a sequence of movements across time and space. This dynamic time-varying information may be more complex than the static feature information that is sufficient for comprehension of most other semantic categories, such as size or shape, and as such may require greater control to selectively access and manipulate the relevant features. This region may perform semantic control for all categories with tools simply requiring a high level of semantic control, or it may have a particular role in integrating information between the praxis network for planning tool use (in the superior parietal lobule and premotor cortices) and the more conceptual temporal lobe system for tool knowledge (Lewis 2006; Lesourd et al. 2021). Other regions implicated in tool processing beyond the pLTC, such as the inferior parietal cortex, may contribute more to the representation of tools than their controlled access and manipulation (Ishibashi et al. 2016; Reynaud et al. 2016; Buxbaum 2017).

#### The relationship between theory of mind and semantics

Theory of mind recruited the same left pLTC and right pSTS subregions as those recruited for semantic representation, including the pSTS and ventral AG. This area is often labeled as the TPJ in theory of mind literature, a region considered to be of particular importance for mentalizing (Saxe and Kanwisher 2003; Carrington and Bailey 2009; Schurz et al. 2014, 2017; Molenberghs et al. 2016). It has previously been established that the theory of mind network includes semantic regions (Olson et al. 2007, 2013; Duval et al. 2012; Binney and Ramsey 2020; Diveica et al. 2021); here, by formally comparing across domains, we have demonstrated that the same regions are recruited for both domains, a similarity that may typically be obscured by the inconsistent use of anatomical terms or the tendency to investigate each domain independently. There are many possibilities for this high degree of overlap in the left pLTC. Many theory of mind tasks involve meaningful stimuli, such as narratives or vignettes, which would necessarily engage semantic processing areas to comprehend the meanings of the words and track meaning—or indeed multiple meanings—over time (Molenberghs et al. 2016; Branzi et al. 2020), and so this overlap may simply reflect a need for the engagement of semantic networks in theory of mind processing (Binney and Ramsey 2020; Diveica et al. 2021). These activations may have been detected in the present meta-analysis due to different theory of mind task conditions not being adequately matched on semantic demands.

Direct contrasts reveal that although the ventral anterior AG is engaged for both domains, activation likelihood is significantly higher for theory of mind than semantics in this region. Furthermore, the right AG was identified for theory of mind but not semantic cognition. This may suggest that the AG is responsible for theory of mind and some of the semantic studies engage relevant processes. However, the majority of semantic studies that were included utilized single words, which should not require theory of mind processing. Indeed, the role of the AG is a focus of great debate within the semantic cognition literature (Graves et al. 2010; Seghier et al. 2010; Binder and Desai 2011; Noonan et al. 2013; Seghier 2013; Davey et al. 2015; Humphreys et al. 2021) and the laterality of the semantic network is task- and stimuli-dependent (Rice et al. 2015, 2018). Similarly, although theory of mind is often considered to rely on right-lateralized areas (Saxe and Wexler 2005; Döhnel et al. 2012), it may be that these laterality differences are present only after subtracting a left-lateralized verbal semantics network through the use of a non-mentalizing story in false belief tasks. Thus, a more nuanced explanation may require consideration of multiple different factors affecting both the recruitment of the AG and the laterality of the network engaged, such as the use of sentences (Branzi et al. 2020; Humphreys et al. 2020), the stimulus modality, and the effect of difficulty-dependent deactivation (Raichle et al. 2001; Humphreys et al. 2015; Jackson et al. 2019), as well as the social nature of the stimuli. Indeed, as the theory of mind dataset contains a large number of tasks with full sentences, whereas the semantics dataset contains many tasks that focus on single words, this region may show relatively greater activation for theory of mind on the basis of stimuli differences and not theory of mind requirements. If this is viewed as one large bilateral network performing both kinds of tasks with variations in the locus of peak activation, a very different picture emerges of the regions responsible for these domains. The overlap between these 2 domains demonstrates the clear need for cross-domain comparisons and careful consideration of terminology. It may be that the questions surrounding the AG and the pLTC in the theory of mind and semantic literature form 2 parts of a single puzzle where progress would be best achieved by bringing these domains together in future research.

### Right-lateralized domains

Some domains engaged the right pLTC to a relatively greater extent than the left. Biological motion had a right hemisphere focus, engaging a distinct region ventral to theory of mind, centered on the most posterior aspect of MTG (posterior to the other left-hemisphere pMTG subregions), immediately ventral to the AG, and anterior to the occipital lobe. This placement is consistent with prior assessments identifying a region in pSTS for processing dynamic biological stimuli, and forms part of the dorsal route for visual input, with biological motion processing necessitating connections from occipital cortex, including the extrastriate body area (a region demonstrating preferential responses to static body stimuli; Allison et al. 2000; Downing et al. 2001, 2006; Vaina et al. 2001; Grossman and Blake 2002; Rizzolatti and Matelli 2003; Astafiev et al. 2004; Spiridon et al. 2006; Taylor et al. 2007; Myers and Sowden 2008; Kontaris et al. 2009; Thompson and Parasuraman 2012; Sokolov et al. 2018). Faces similarly showed a right hemisphere focus, with only a small cluster in the left hemisphere, located in the homologue of the right pSTS cluster. It is also notable that the overall pattern of relative lateralization in the biological motion and faces domains seen in the present analysis is consistent with the suggestion that social domains are typically right-lateralized (Grossman et al. 2000; Saxe et al. 2004; Saxe and Wexler 2005; Rossion et al. 2012; Dasgupta et al. 2017), and with connectivity analyses that find the right pSTS to be more strongly connected to other biological motion and face processing regions in the right hemisphere, than the left pSTS is to its counterparts (Dasgupta et al. 2017). However, it is important to be cautious when interpreting lateralization in the present results, as a lack of extensive left hemisphere activation likelihood for a given domain does not prove that additional left pLTC regions are not recruited. Formal laterality analyses may be warranted in future studies, to investigate this more closely.

Although distinct regions, the proximity of theory of mind and biological motion areas may support crucial interactions between these domains. Both of these regions demonstrate partial overlap with a small region identified for faces around their border in the right pSTS. This corresponds to a known face-responsive area that is specialized for processing changeable aspects of a face, such as expression, lip movement, and eye gaze (Haxby et al. 2000; Hoffman and Haxby 2000; O’Toole et al. 2002; Calder and Young 2005; Bernstein and Yovel 2015), and which has been argued to constitute part of a third visual pathway for social perception (Pitcher and Ungerleider 2021), although this region is typically reported to be bilateral (O’Toole et al. 2002; Bernstein and Yovel 2015; Müller et al. 2018b). The relatively greater extent of activation likelihood in the right hemisphere in the present analysis could be due to biases in the literature, such as focusing on the right hemisphere for faces or assessing nonspeech facial expressions and movements (De Winter et al. 2015). This face region falls entirely within areas implicated in theory of mind and biological motion without displaying significantly greater engagement for faces. Indeed, when revisiting the prior literature with this result in mind, it is not clear that this face pSTS region has been reliably differentiated from the proposed pSTS region for biological motion (Allison et al. 2000; Peelen et al. 2006; Engell and McCarthy 2013). Similarly, though the cluster for faces in the right posterior MTG near the border with the occipital lobe may correspond to a proposed face-selective region known as the occipital face area (OFA; Gauthier et al. 2000; Haxby et al. 2000; Pitcher et al. 2011), this same region appeared in the conjunction for faces and biological motion with no significant voxels in the faces over biological motion subtraction analysis. Taken together with the lack of significant results for faces over semantics or theory of mind in the left hemisphere, these present results do not suggest the existence of a face-specific region in the pLTC. It is, of course, possible that this region responds differentially across semantic categories, or that it contains distinct neural populations subserving each domain, but these possibilities cannot be disentangled with meta-analyses or group-level analyses. Alternatively, the engagement of this region for faces may simply reflect the common engagement of biological motion and theory of mind processes in studies employing face stimuli. The processing of dynamic and changeable aspects of faces may be subserved by the pSTS as a subset of all dynamic body stimuli. Thus, this region may not be specialized for faces exclusively, but instead for the processing of dynamic biological stimuli in ventral aspects and for information about intentional action more broadly in dorsal aspects, of which expression and facial movements are a subset. The extensive connectivity of the pSTS with regions that subserve social processing tasks, such as the perception of salient social stimuli, the observation and understanding of intentional action, and the attribution of mental states could support this interpretation and may suggest a particular role for this region as a hub or interface between networks supporting distinct tasks (Yang et al. 2015; Dasgupta et al. 2017).

## Conclusions

The application of a cross-domain approach here has helped elucidate the wider organization of the pLTC, illuminating possible subregions and highlighting previously obscured relationships between different domains. Direct comparison utilizing a large amount of data across domains, using the ALE method, allowed for a single high-powered study in contrast to a single task-based neuroimaging study, which would have far smaller samples, may be underpowered (Ioannidis 2005; Szucs and Ioannidis 2020), and must rely upon single, often idiosyncratic, tasks. Here, we have been able to include thousands of participants, and capture common activations across the different variations of tasks used to assess a process or domain. The hypotheses generated here may be tested within individual participants, to further examine to what extent apparent overlapping activation is indeed due to shared regions or processes.

## Funding

This work was supported by a Biotechnology and Biological Sciences Research Council studentship to V.J.H, a British Academy Postdoctoral Fellowship awarded to R.L.J. (grant no. pf170068), a programme grant to M.A.L.R. from the Medical Research Council (grant no. MR/R023883/1), an Advanced Grant from the European Research Council to M.A.L.R. (GAP: 670428) and Medical Research Council intramural funding (grant no. MC_UU_00005/18).

*Conflict of interest statement*: The authors declare no competing interest.

## Authors’ Contributions

All authors conceived the study. VJH and RLJ collected and analyzed data, VJH wrote the initial draft and RLJ and MALR revised the manuscript.

## Data and Code Availability

The toolboxes used to analyze the data are freely available from www.brainmap.org/ale. The code used can be found in Supplementary Materials 1; foci files and results files can be found on GitHub https://github.com/Vicki-H/Cross-domain-pLTC.

## Ethics

No new data were collected for this study. All data were taken from published works that reported on samples of consenting participants.

For the purpose of open access, the author has applied a Creative Commons Attribution (CC BY) license to any Author Accepted Manuscript version arising from this submission.

## Supplementary Material

Final_Supplementary_Hodgson_bhac394

## References

[ref1] Allison T , Puce A, McCarthy G. Social perception from visual cues: role of the STS region. Trends Cogn Sci. 2000:4:267–278. 10859571 10.1016/s1364-6613(00)01501-1

[ref2] Assem M , Glasser MF, Van Essen DC, Duncan J. A domain-general cognitive core defined in multimodally parcellated human cortex. Cereb Cortex. 2020:30:4361–4380. 32244253 10.1093/cercor/bhaa023 PMC7325801

[ref3] Astafiev SV , Stanley CM, Shulman GL, Corbetta M. Extrastriate body area in human occipital cortex responds to the performance of motor actions. Nat Neurosci. 2004:7:542–548. 15107859 10.1038/nn1241

[ref4] Bajada CJ , Jackson RL, Haroon HA, Azadbakht H, Parker GJM, Lambon Ralph MA, Cloutman LL. A graded tractographic parcellation of the temporal lobe. NeuroImage. 2017:155:503–512. 28411156 10.1016/j.neuroimage.2017.04.016 PMC5518769

[ref5] Baron-Cohen S , Wheelwright S, Hill J, Raste Y, Plumb I. The “reading the mind in the eyes” test revised version: a study with normal adults, and adults with asperger syndrome or high-functioning autism. J Child Psychol Psychiatry. 2001:42:241–251. 11280420

[ref6] Bates E , Wilson SM, Saygin AP, Dick F, Sereno MI, Knight RT, Dronkers NF. Voxel-based lesion–symptom mapping. Nat Neurosci. 2003:6:448–450. 12704393 10.1038/nn1050

[ref7] Beauchamp MS , Lee KE, Haxby JV, Martin A. fMRI responses to video and point-light displays of moving humans and manipulable objects. J Cogn Neurosci. 2003:15:991–1001. 14614810 10.1162/089892903770007380

[ref8] Bernstein M , Yovel G. Two neural pathways of face processing: a critical evaluation of current models. Neurosci Biobehav Rev. 2015:55:536–546. 26067903 10.1016/j.neubiorev.2015.06.010

[ref9] Binder JR , Desai RH. The neurobiology of semantic memory. Trends Cogn Sci. 2011:15:527–536. 22001867 10.1016/j.tics.2011.10.001 PMC3350748

[ref10] Binder JR , Frost JA, Hammeke TA, Bellgowan PSF, Springer JA, Kaufman JN, Possing ET. Human temporal lobe activation by speech and nonspeech sounds. Cereb Cortex. 2000:10:512–528. 10847601 10.1093/cercor/10.5.512

[ref11] Binder JR , Desai RH, Graves WW, Conant LL. Where is the semantic system? A critical review and meta-analysis of 120 functional neuroimaging studies. Cereb Cortex. 2009:19:2767–2796. 19329570 10.1093/cercor/bhp055 PMC2774390

[ref12] Binney RJ , Ramsey R. Social semantics: the role of conceptual knowledge and cognitive control in a neurobiological model of the social brain. Neurosci Biobehav Rev. 2020:112:28–38. 31982602 10.1016/j.neubiorev.2020.01.030

[ref13] Bonda E , Petrides M, Ostry D, Evans A. Specific involvement of human parietal systems and the amygdala in the perception of biological motion. J Neurosci. 1996:16:3737–3744. 8642416 10.1523/JNEUROSCI.16-11-03737.1996 PMC6578830

[ref14] Boronat CB , Buxbaum LJ, Coslett HB, Tang K, Saffran EM, Kimberg DY, Detre JA. Distinctions between manipulation and function knowledge of objects: evidence from functional magnetic resonance imaging. Cogn Brain Res. 2005:23:361–373. 10.1016/j.cogbrainres.2004.11.001 15820643

[ref15] Branzi FM , Humphreys GF, Hoffman P, Lambon Ralph MA. Revealing the neural networks that extract conceptual gestalts from continuously evolving or changing semantic contexts. NeuroImage. 2020:220:116802. 32283276 10.1016/j.neuroimage.2020.116802 PMC7573538

[ref16] Brodmann K . Vergleichende Lokalisationslehre der Grosshirnrinde in ihren Prinzipien dargestellt auf Grund des Zellenbaues. Leipzig, Germany: Johann Ambrosius Barth; 1909.

[ref17] Buchsbaum BR , Baldo J, Okada K, Berman KF, Dronkers N, D’Esposito M, Hickok G. Conduction aphasia, sensory-motor integration, and phonological short-term memory – an aggregate analysis of lesion and fMRI data. Brain Lang. 2011:119:119–128. 21256582 10.1016/j.bandl.2010.12.001 PMC3090694

[ref18] Buxbaum LJ . Learning, remembering, and predicting how to use tools: distributed neurocognitive mechanisms: comment on Osiurak and Badets (2016). Psychol Rev. 2017:124:346–360. 28358565 10.1037/rev0000051 PMC5375056

[ref19] Calder AJ , Young AW. Understanding the recognition of facial identity and facial expression. Nat Rev Neurosci. 2005:6:641–651. 16062171 10.1038/nrn1724

[ref20] Camilleri JA , Müller VI, Fox P, Laird AR, Hoffstaedter F, Kalenscher T, Eickhoff SB. Definition and characterization of an extended multiple-demand network. NeuroImage. 2018:165:138–147. 29030105 10.1016/j.neuroimage.2017.10.020 PMC5732056

[ref21] Campanella F , D’Agostini S, Skrap M, Shallice T. Naming manipulable objects: anatomy of a category specific effect in left temporal tumours. Neuropsychologia. 2010:48:1583–1597. 20144630 10.1016/j.neuropsychologia.2010.02.002

[ref22] Carrington SJ , Bailey AJ. Are there theory of mind regions in the brain? A review of the neuroimaging literature. Hum Brain Mapp. 2009:30:2313–2335. 19034900 10.1002/hbm.20671 PMC6871093

[ref23] Carter RM , Huettel SA. A nexus model of the temporal–parietal junction. Trends Cogn Sci. 2013:17:328–336. 23790322 10.1016/j.tics.2013.05.007 PMC3750983

[ref24] Chao LL , Haxby JV, Martin A. Attribute-based neural substrates in temporal cortex for perceiving and knowing about objects. Nat Neurosci. 1999a:2:913–919. 10491613 10.1038/13217

[ref25] Chao LL , Martin A, Haxby JV. Are face-responsive regions selective only for faces? Neuroreport. 1999b:10:2945–2950. 10549802 10.1097/00001756-199909290-00013

[ref26] Chen L , Rogers TT. Revisiting domain-general accounts of category specificity in mind and brain. WIREs Cogn Sci. 2014:5:327–344. 10.1002/wcs.1283 26308567

[ref27] Chen L , Lambon Ralph MA, Rogers TT. A unified model of human semantic knowledge and its disorders. Nat Hum Behav. 2017:1:1–10. 10.1038/s41562-016-0039 PMC5417358 28480333

[ref28] Cohen L , Dehaene S, Naccache L, Lehéricy S, Dehaene-Lambertz G, Hénaff M-A, Michel F. The visual word form area: spatial and temporal characterization of an initial stage of reading in normal subjects and posterior split-brain patients. Brain. 2000:123:291–307. 10648437 10.1093/brain/123.2.291

[ref29] Corbett F , Jefferies E, Ehsan S, Lambon Ralph MA. Different impairments of semantic cognition in semantic dementia and semantic aphasia: evidence from the non-verbal domain. Brain. 2009:132:2593–2608. 19506072 10.1093/brain/awp146 PMC2766180

[ref30] Creem-Regehr SH , Lee JN. Neural representations of graspable objects: are tools special? Cogn Brain Res. 2005:22:457–469. 10.1016/j.cogbrainres.2004.10.006 15722215

[ref31] Damasio H , Damasio AR. The anatomical basis of conduction aphasia. Brain. 1980:103:337–350. 7397481 10.1093/brain/103.2.337

[ref32] Dasgupta S , Tyler SC, Wicks J, Srinivasan R, Grossman ED. Network connectivity of the right STS in three social perception localizers. J Cogn Neurosci. 2017:29:221–234. 27991030 10.1162/jocn_a_01054

[ref33] Davey J , Cornelissen PL, Thompson HE, Sonkusare S, Hallam G, Smallwood J, Jefferies E. Automatic and controlled semantic retrieval: TMS reveals distinct contributions of posterior middle temporal gyrus and angular gyrus. J Neurosci. 2015:35:15230–15239. 26586812 10.1523/JNEUROSCI.4705-14.2015 PMC4649000

[ref34] Davey J , Thompson HE, Hallam G, Karapanagiotidis T, Murphy C, De Caso I, Krieger-Redwood K, Bernhardt BC, Smallwood J, Jefferies E. Exploring the role of the posterior middle temporal gyrus in semantic cognition: integration of anterior temporal lobe with executive processes. NeuroImage. 2016:137:165–177. 27236083 10.1016/j.neuroimage.2016.05.051 PMC4927261

[ref35] De Winter F-L , Zhu Q, Van den Stock J, Nelissen K, Peeters R, de Gelder B, Vanduffel W, Vandenbulcke M. Lateralization for dynamic facial expressions in human superior temporal sulcus. NeuroImage. 2015:106:340–352. 25463458 10.1016/j.neuroimage.2014.11.020

[ref36] Diveica V , Koldewyn K, Binney RJ. Establishing a role of the semantic control network in social cognitive processing: a meta-analysis of functional neuroimaging studies. NeuroImage. 2021:245:118702. 34742940 10.1016/j.neuroimage.2021.118702

[ref37] Döhnel K , Schuwerk T, Meinhardt J, Sodian B, Hajak G, Sommer M. Functional activity of the right temporo-parietal junction and of the medial prefrontal cortex associated with true and false belief reasoning. NeuroImage. 2012:60:1652–1661. 22300812 10.1016/j.neuroimage.2012.01.073

[ref38] Downing PE , Jiang Y, Shuman M, Kanwisher N. A cortical area selective for visual processing of the human body. Science. 2001:293:2470–2473. 11577239 10.1126/science.1063414

[ref39] Downing PE , Peelen MV, Wiggett AJ, Tew BD. The role of the extrastriate body area in action perception. Soc Neurosci. 2006:1:52–62. 18633775 10.1080/17470910600668854

[ref40] Duncan J . The multiple-demand (MD) system of the primate brain: mental programs for intelligent behaviour. Trends Cogn Sci. 2010:14:172–179. 20171926 10.1016/j.tics.2010.01.004

[ref41] Duval C , Bejanin A, Piolino P, Laisney M, de La Sayette V, Belliard S, Eustache F, Desgranges B. Theory of mind impairments in patients with semantic dementia. Brain. 2012:135:228–241. 22232593 10.1093/brain/awr309 PMC3655376

[ref42] Ebisch SJH , Babiloni C, Del Gratta C, Ferretti A, Perrucci MG, Caulo M, Sitskoorn MM, Romani GL. Human neural systems for conceptual knowledge of proper object use: a functional magnetic resonance imaging study. Cereb Cortex. 2007:17:2744–2751. 17283202 10.1093/cercor/bhm001

[ref43] Eickhoff SB , Laird AR, Grefkes C, Wang LE, Zilles K, Fox PT. Coordinate-based activation likelihood estimation meta-analysis of neuroimaging data: a random-effects approach based on empirical estimates of spatial uncertainty. Hum Brain Mapp. 2009:30:2907–2926. 19172646 10.1002/hbm.20718 PMC2872071

[ref44] Eickhoff SB , Bzdok D, Laird AR, Kurth F, Fox PT. Activation likelihood estimation meta-analysis revisited. NeuroImage. 2012:59:2349–2361. 21963913 10.1016/j.neuroimage.2011.09.017 PMC3254820

[ref45] Eickhoff SB , Laird AR, Fox PM, Lancaster JL, Fox PT. Implementation errors in the GingerALE Software: description and recommendations. Hum Brain Mapp. 2017:38:7–11. 27511454 10.1002/hbm.23342 PMC5323082

[ref46] Engell AD , McCarthy G. Probabilistic atlases for face and biological motion perception: an analysis of their reliability and overlap. NeuroImage. 2013:74:140–151. 23435213 10.1016/j.neuroimage.2013.02.025 PMC3690657

[ref47] Epstein R , Harris A, Stanley D, Kanwisher N. The parahippocampal place area: recognition, navigation, or encoding? Neuron. 1999:23:115–125. 10402198 10.1016/s0896-6273(00)80758-8

[ref48] Fedorenko E , Duncan J, Kanwisher N. Broad domain generality in focal regions of frontal and parietal cortex. Proc Natl Acad Sci. 2013:110:16616–16621. 24062451 10.1073/pnas.1315235110 PMC3799302

[ref49] Friederici AD . Pathways to language: fiber tracts in the human brain. Trends Cogn Sci. 2009:13:175–181. 19223226 10.1016/j.tics.2009.01.001

[ref50] Friederici AD . The brain basis of language processing: from structure to function. Physiol Rev. 2011:91:1357–1392. 22013214 10.1152/physrev.00006.2011

[ref51] Friederici AD , Rüschemeyer S-A, Hahne A, Fiebach CJ. The role of left inferior frontal and superior temporal cortex in sentence comprehension: localizing syntactic and semantic processes. Cereb Cortex. 2003:13:170–177. 12507948 10.1093/cercor/13.2.170

[ref52] Friederici AD , Makuuchi M, Bahlmann J. The role of the posterior superior temporal cortex in sentence comprehension. Neuroreport. 2009:20:563–568. 19287322 10.1097/WNR.0b013e3283297dee

[ref53] Frith CD , Frith U. The neural basis of mentalizing. Neuron. 2006:50:531–534. 16701204 10.1016/j.neuron.2006.05.001

[ref54] Gao Z , Zheng L, Chiou R, Gouws A, Krieger-Redwood K, Wang X, Varga D, Ralph MAL, Smallwood J, Jefferies E. Distinct and common neural coding of semantic and non-semantic control demands. NeuroImage. 2021:236:118230. 34089873 10.1016/j.neuroimage.2021.118230 PMC8271095

[ref55] Gardner HE , Lambon Ralph MA, Dodds N, Jones T, Ehsan S, Jefferies E. The differential contributions of pFC and temporo-parietal cortex to multimodal semantic control: exploring refractory effects in semantic aphasia. J Cogn Neurosci. 2012:24:778–793. 22220727 10.1162/jocn_a_00184

[ref56] Gauthier I , Tarr MJ, Moylan J, Skudlarski P, Gore JC, Anderson AW. The fusiform “face area” is part of a network that processes faces at the individual level. J Cogn Neurosci. 2000:12:495–504. 10931774 10.1162/089892900562165

[ref57] Graves WW , Grabowski TJ, Mehta S, Gupta P. The left posterior superior temporal gyrus participates specifically in accessing lexical phonology. J Cogn Neurosci. 2008:20:1698–1710. 18345989 10.1162/jocn.2008.20113 PMC2570618

[ref58] Graves WW , Binder JR, Desai RH, Conant LL, Seidenberg MS. Neural correlates of implicit and explicit combinatorial semantic processing. NeuroImage. 2010:53:638–646. 20600969 10.1016/j.neuroimage.2010.06.055 PMC2930088

[ref59] Grosbras M-H , Beaton S, Eickhoff SB. Brain regions involved in human movement perception: a quantitative voxel-based meta-analysis. Hum Brain Mapp. 2012:33:431–454. 21391275 10.1002/hbm.21222 PMC6869986

[ref60] Grossman ED , Blake R. Brain areas active during visual perception of biological motion. Neuron. 2002:35:1167–1175. 12354405 10.1016/s0896-6273(02)00897-8

[ref61] Grossman E , Donnelly M, Price R, Pickens D, Morgan V, Neighbor G, Blake R. Brain areas involved in perception of biological motion. J Cogn Neurosci. 2000:12:711–720. 11054914 10.1162/089892900562417

[ref62] Haxby JV , Ungerleider LG, Clark VP, Schouten JL, Hoffman EA, Martin A. The effect of face inversion on activity in human neural systems for face and object perception. Neuron. 1999:22:189–199. 10027301 10.1016/s0896-6273(00)80690-x

[ref63] Haxby JV , Hoffman EA, Gobbini MI. The distributed human neural system for face perception. Trends Cogn Sci. 2000:4:223–233. 10827445 10.1016/s1364-6613(00)01482-0

[ref64] Hickok G . Chapter 4 - speech perception, conduction aphasia, and the functional neuroanatomy of language. In: Grodzinsky Y, Shapiro LP, Swinney D, editors. Language and the Brain. Foundations of Neuropsychology. San Diego: Academic Press; 2000. pp. 87–104

[ref65] Hickok G , Poeppel D. Dorsal and ventral streams: a framework for understanding aspects of the functional anatomy of language. Cognition, Towards a New Functional Anatomy of Language. 2004:92:67–99. 10.1016/j.cognition.2003.10.011 15037127

[ref66] Hickok G , Poeppel D. The cortical organization of speech processing. Nat Rev Neurosci. 2007:8:393–402. 17431404 10.1038/nrn2113

[ref67] Hodges JR , Patterson K. Nonfluent progressive aphasia and semantic dementia: a comparative neuropsychological study. J Int Neuropsychol Soc. 1996:2:511–524. 9375155 10.1017/s1355617700001685

[ref68] Hodgson VJ , Lambon Ralph MA, Jackson RL. Multiple dimensions underlying the functional organization of the language network. NeuroImage. 2021:241:118444. 34343627 10.1016/j.neuroimage.2021.118444 PMC8456749

[ref69] Hoffman EA , Haxby JV. Distinct representations of eye gaze and identity in the distributed human neural system for face perception. Nat Neurosci. 2000:3:80–84. 10607399 10.1038/71152

[ref70] Hooker CI , Paller KA, Gitelman DR, Parrish TB, Mesulam M-M, Reber PJ. Brain networks for analyzing eye gaze. Cogn Brain Res. 2003:17:406–418. 10.1016/s0926-6410(03)00143-5 PMC4346170 12880911

[ref71] Humphreys GF , Lambon Ralph MA. Fusion and fission of cognitive functions in the human parietal cortex. Cereb Cortex. 2015:25:3547–3560. 25205661 10.1093/cercor/bhu198 PMC4585503

[ref72] Humphreys GF , Hoffman P, Visser M, Binney RJ, Ralph MAL. Establishing task- and modality-dependent dissociations between the semantic and default mode networks. Proc Natl Acad Sci. 2015:112:7857–7862. 26056304 10.1073/pnas.1422760112 PMC4485123

[ref73] Humphreys GF , Jackson RL, Lambon Ralph MA. Overarching principles and dimensions of the functional organization in the inferior parietal cortex. Cereb Cortex. 2020:30:5639–5653. 32515783 10.1093/cercor/bhaa133 PMC7116231

[ref74] Humphreys GF , Lambon Ralph MA, Simons JS. A unifying account of angular gyrus contributions to episodic and semantic cognition. Trends Neurosci. 2021:44:452–463. 33612312 10.1016/j.tins.2021.01.006

[ref75] Ioannidis JPA . Why most published research findings are false. PLoS Med. 2005:2:e124. 16060722 10.1371/journal.pmed.0020124 PMC1182327

[ref76] Ishibashi R , Pobric G, Saito S, Ralph MAL. The neural network for tool-related cognition: An activation likelihood estimation meta-analysis of 70 neuroimaging contrasts. Cogn Neuropsychol. 2016:33:241–256. 27362967 10.1080/02643294.2016.1188798 PMC4989859

[ref77] Jackson RL . The neural correlates of semantic control revisited. NeuroImage. 2020:224:117444. 33059049 10.1016/j.neuroimage.2020.117444 PMC7779562

[ref78] Jackson RL , Bajada CJ, Rice GE, Cloutman LL, Lambon Ralph MA. An emergent functional parcellation of the temporal cortex. NeuroImage, Segmenting the Brain. 2018:170:385–399. 10.1016/j.neuroimage.2017.04.024 28419851

[ref79] Jackson RL , Cloutman LL, Lambon Ralph MA. Exploring distinct default mode and semantic networks using a systematic ICA approach. Cortex. 2019:113:279–297. 30716610 10.1016/j.cortex.2018.12.019 PMC6459395

[ref80] Jackson RL , Rogers TT, Lambon Ralph MA. Reverse-engineering the cortical architecture for controlled semantic cognition. Nat Hum Behav. 2021:5:774–786. 33462472 10.1038/s41562-020-01034-z PMC7611056

[ref81] Jefferies E . The neural basis of semantic cognition: converging evidence from neuropsychology, neuroimaging and TMS. Cortex. 2013:49:611–625. 23260615 10.1016/j.cortex.2012.10.008

[ref82] Jefferies E , Lambon Ralph MA. Semantic impairment in stroke aphasia versus semantic dementia: a case-series comparison. Brain. 2006:129:2132–2147. 16815878 10.1093/brain/awl153

[ref83] Kanwisher N . The quest for the FFA and where it led. J Neurosci. 2017:37:1056–1061. 28148806 10.1523/JNEUROSCI.1706-16.2016 PMC5296790

[ref84] Kanwisher N , McDermott J, Chun MM. The fusiform face area: a module in human extrastriate cortex specialized for face perception. J Neurosci. 1997:17:4302–4311. 9151747 10.1523/JNEUROSCI.17-11-04302.1997 PMC6573547

[ref85] Kontaris I , Wiggett AJ, Downing PE. Dissociation of extrastriate body and biological-motion selective areas by manipulation of visual-motor congruency. Neuropsychologia. 2009:47:3118–3124. 19643118 10.1016/j.neuropsychologia.2009.07.012 PMC2935969

[ref86] Laird AR , Fox PM, Price CJ, Glahn DC, Uecker AM, Lancaster JL, Turkeltaub PE, Kochunov P, Fox PT. ALE meta-analysis: controlling the false discovery rate and performing statistical contrasts. Hum Brain Mapp. 2005:25:155–164. 15846811 10.1002/hbm.20136 PMC6871747

[ref87] Lambon Ralph MAL , Jefferies E, Patterson K, Rogers TT. The neural and computational bases of semantic cognition. Nat Rev Neurosci. 2017:18:42–55. 27881854 10.1038/nrn.2016.150

[ref88] Lancaster JL , Tordesillas-Gutiérrez D, Martinez M, Salinas F, Evans A, Zilles K, Mazziotta JC, Fox PT. Bias between MNI and Talairach coordinates analyzed using the ICBM-152 brain template. Hum Brain Mapp. 2007:28:1194–1205. 17266101 10.1002/hbm.20345 PMC6871323

[ref89] Lesourd M , Servant M, Baumard J, Reynaud E, Ecochard C, Medjaoui FT, Bartolo A, Osiurak F. Semantic and action tool knowledge in the brain: identifying common and distinct networks. Neuropsychologia. 2021:159:107918. 34166668 10.1016/j.neuropsychologia.2021.107918

[ref90] Lewis J . Cortical networks related to human use of tools. Neurosci Rev J Bringing Neurobiol Neurol Psychiatry. 2006:12(3):211–231. 10.1177/1073858406288327 16684967

[ref91] McCandliss BD , Cohen L, Dehaene S. The visual word form area: expertise for reading in the fusiform gyrus. Trends Cogn Sci. 2003:7:293–299. 12860187 10.1016/s1364-6613(03)00134-7

[ref92] Mesulam M-M , Rogalski EJ, Wieneke C, Hurley RS, Geula C, Bigio EH, Thompson CK, Weintraub S. Primary progressive aphasia and the evolving neurology of the language network. Nat Rev Neurol. 2014:10:554–569. 25179257 10.1038/nrneurol.2014.159 PMC4201050

[ref93] Molenberghs P , Johnson H, Henry JD, Mattingley JB. Understanding the minds of others: a neuroimaging meta-analysis. Neurosci Biobehav Rev. 2016:65:276–291. 27073047 10.1016/j.neubiorev.2016.03.020

[ref94] Müller VI , Cieslik EC, Laird AR, Fox PT, Radua J, Mataix-Cols D, Tench CR, Yarkoni T, Nichols TE, Turkeltaub PE, et al. Ten simple rules for neuroimaging meta-analysis. Neurosci Biobehav Rev. 2018a:84:151–161. 29180258 10.1016/j.neubiorev.2017.11.012 PMC5918306

[ref95] Müller VI , Höhner Y, Eickhoff SB. Influence of task instructions and stimuli on the neural network of face processing: an ALE meta-analysis. Cortex. 2018b:103:240–255. 29665467 10.1016/j.cortex.2018.03.011 PMC5988961

[ref96] Myers A , Sowden PT. Your hand or mine? The extrastriate body area. NeuroImage. 2008:42:1669–1677. 18586108 10.1016/j.neuroimage.2008.05.045

[ref97] Noonan KA , Jefferies E, Corbett F, Lambon Ralph MA. Elucidating the nature of deregulated semantic cognition in semantic aphasia: evidence for the roles of prefrontal and temporo-parietal cortices. J Cogn Neurosci. 2009:22:1597–1613. 10.1162/jocn.2009.21289 19580383

[ref98] Noonan KA , Jefferies E, Visser M, Lambon Ralph MA. Going beyond inferior prefrontal involvement in semantic control: evidence for the additional contribution of dorsal angular gyrus and posterior middle temporal cortex. J Cogn Neurosci. 2013:25:1824–1850. 23859646 10.1162/jocn_a_00442

[ref99] O’Toole AJ , Roark DA, Abdi H. Recognizing moving faces: a psychological and neural synthesis. Trends Cogn Sci. 2002:6:261–266. 12039608 10.1016/s1364-6613(02)01908-3

[ref100] Olson IR , Plotzker A, Ezzyat Y. The Enigmatic temporal pole: a review of findings on social and emotional processing. Brain. 2007:130:1718–1731. 17392317 10.1093/brain/awm052

[ref101] Olson IR , McCoy D, Klobusicky E, Ross LA. Social cognition and the anterior temporal lobes: a review and theoretical framework. Soc Cogn Affect Neurosci. 2013:8:123–133. 23051902 10.1093/scan/nss119 PMC3575728

[ref102] Peelen MV , Downing PE. Selectivity for the human body in the fusiform gyrus. J Neurophysiol. 2005:93:603–608. 15295012 10.1152/jn.00513.2004

[ref103] Peelen MV , Downing PE. The neural basis of visual body perception. Nat Rev Neurosci. 2007:8:636–648. 17643089 10.1038/nrn2195

[ref104] Peelen MV , Wiggett AJ, Downing PE. Patterns of fMRI activity dissociate overlapping functional brain areas that respond to biological motion. Neuron. 2006:49:815–822. 16543130 10.1016/j.neuron.2006.02.004

[ref105] Perrett DI , Hietanen JK, Oram MW, Benson PJ, Rolls ET, Bruce V, Cowey A, Ellis AW, Perrett DI. Organization and functions of cells responsive to faces in the temporal cortex. Philos Trans R Soc Lond Ser B Biol Sci. 1992:335:23–30. 1348133 10.1098/rstb.1992.0003

[ref106] Phillips ML , Young AW, Senior C, Brammer M, Andrew C, Calder AJ, Bullmore ET, Perrett DI, Rowland D, Williams SCR, et al. A specific neural substrate for perceiving facial expressions of disgust. Nature. 1997:389:495–498. 9333238 10.1038/39051

[ref107] Pitcher D , Ungerleider LG. Evidence for a third visual pathway specialized for social perception. Trends Cogn Sci. 2021:25:100–110. 33334693 10.1016/j.tics.2020.11.006 PMC7811363

[ref108] Pitcher D , Walsh V, Duchaine B. The role of the occipital face area in the cortical face perception network. Exp Brain Res. 2011:209:481–493. 21318346 10.1007/s00221-011-2579-1

[ref109] Poeppel D . Pure word deafness and the bilateral processing of the speech code. Cogn Sci. 2001:25:679–693.

[ref110] Pourtois G , Schwartz S, Spiridon M, Martuzzi R, Vuilleumier P. Object representations for multiple visual categories overlap in lateral occipital and medial fusiform cortex. Cereb Cortex. 2009:19:1806–1819. 19015371 10.1093/cercor/bhn210

[ref111] Price CJ , Noppeney U, Phillips J, Devlin JT. How is the fusiform gyrus related to category-specificity? Cogn Neuropsychol. 2003:20:561–574. 20957585 10.1080/02643290244000284

[ref112] Puce A , Allison T, Bentin S, Gore JC, McCarthy G. Temporal cortex activation in humans viewing eye and mouth movements. J Neurosci. 1998:18:2188–2199. 9482803 10.1523/JNEUROSCI.18-06-02188.1998 PMC6792917

[ref113] Raichle ME , MacLeod AM, Snyder AZ, Powers WJ, Gusnard DA, Shulman GL. A default mode of brain function. Proc Natl Acad Sci. 2001:98:676–682. 11209064 10.1073/pnas.98.2.676 PMC14647

[ref114] Reynaud E , Lesourd M, Navarro J, Osiurak F. On the neurocognitive origins of human tool use : a critical review of neuroimaging data. Neurosci Biobehav Rev. 2016:64:421–437. 26976352 10.1016/j.neubiorev.2016.03.009

[ref115] Rice GE , Lambon Ralph MA, Hoffman P. The roles of left versus right anterior temporal lobes in conceptual knowledge: an ALE meta-analysis of 97 functional neuroimaging studies. Cereb Cortex. 2015:25:4374–4391. 25771223 10.1093/cercor/bhv024 PMC4816787

[ref116] Rice GE , Caswell H, Moore P, Hoffman P, Lambon Ralph MA. The roles of left versus right anterior temporal lobes in semantic memory: a neuropsychological comparison of postsurgical temporal lobe epilepsy patients. Cereb Cortex. 2018:28:1487–1501. 29351584 10.1093/cercor/bhx362 PMC6093325

[ref117] Rizzolatti G , Matelli M. Two different streams form the dorsal visual system: anatomy and functions. Exp Brain Res. 2003:153:146–157. 14610633 10.1007/s00221-003-1588-0

[ref118] Robson H , Sage K, Lambon Ralph MA. Wernicke’s aphasia reflects a combination of acoustic-phonological and semantic control deficits: a case-series comparison of Wernicke’s aphasia, semantic dementia and semantic aphasia. Neuropsychologia. 2012:50:266–275. 22178742 10.1016/j.neuropsychologia.2011.11.021

[ref119] Rogers TT , Lambon Ralph MA, Garrard P, Bozeat S, McClelland JL, Hodges JR, Patterson K. Structure and deterioration of semantic memory: a neuropsychological and computational investigation. Psychol Rev. 2004:111:205–235. 14756594 10.1037/0033-295X.111.1.205

[ref120] Rogers TT , Hocking J, Mechelli A, Patterson K, Price C. Fusiform activation to animals is driven by the process, not the stimulus. J Cogn Neurosci. 2005:17:434–445. 15814003 10.1162/0898929053279531

[ref121] Rogers TT , Patterson K, Jefferies E, Lambon Ralph MA. Disorders of representation and control in semantic cognition: effects of familiarity, typicality, and specificity. Neuropsychologia, Spec Iss: Semantic Cognition. 2015:76:220–239. 10.1016/j.neuropsychologia.2015.04.015 PMC4582808 25934635

[ref122] Rossion B , Hanseeuw B, Dricot L. Defining face perception areas in the human brain: a large-scale factorial fMRI face localizer analysis. Brain Cogn. 2012:79:138–157. 22330606 10.1016/j.bandc.2012.01.001

[ref123] Saxe R . Uniquely human social cognition. Curr Opin Neurobiol, Cognitive Neurosci. 2006:16:235–239. 10.1016/j.conb.2006.03.001 16546372

[ref124] Saxe R , Kanwisher N. People thinking about thinking people: The role of the temporo-parietal junction in theory of mind. NeuroImage. 2003:19:1835–1842. 12948738 10.1016/s1053-8119(03)00230-1

[ref125] Saxe R , Wexler A. Making sense of another mind: The role of the right temporo-parietal junction. Neuropsychologia. 2005:43:1391–1399. 15936784 10.1016/j.neuropsychologia.2005.02.013

[ref126] Saxe R , Xiao D-K, Kovacs G, Perrett DI, Kanwisher N. A region of right posterior superior temporal sulcus responds to observed intentional actions. Neuropsychologia. 2004:42:1435–1446. 15246282 10.1016/j.neuropsychologia.2004.04.015

[ref127] Schurz M , Radua J, Aichhorn M, Richlan F, Perner J. Fractionating theory of mind: A meta-analysis of functional brain imaging studies. Neurosci Biobehav Rev. 2014:42:9–34. 24486722 10.1016/j.neubiorev.2014.01.009

[ref128] Schurz M , Tholen MG, Perner J, Mars RB, Sallet J. Specifying the brain anatomy underlying temporo-parietal junction activations for theory of mind: a review using probabilistic atlases from different imaging modalities. Hum Brain Mapp. 2017:38:4788–4805. 28608647 10.1002/hbm.23675 PMC6867045

[ref129] Scott SK , Blank CC, Rosen S, Wise RJS. Identification of a pathway for intelligible speech in the left temporal lobe. Brain. 2000:123:2400–2406. 11099443 10.1093/brain/123.12.2400 PMC5630088

[ref130] Seghier ML . The angular gyrus: multiple functions and multiple subdivisions. Neuroscientist. 2013:19:43–61. 22547530 10.1177/1073858412440596 PMC4107834

[ref131] Seghier ML , Fagan E, Price CJ. Functional subdivisions in the left angular gyrus where the semantic system meets and diverges from the default network. J Neurosci. 2010:30:16809–16817. 21159952 10.1523/JNEUROSCI.3377-10.2010 PMC3105816

[ref132] Sokolov AA , Zeidman P, Erb M, Ryvlin P, Friston KJ, Pavlova MA. Structural and effective brain connectivity underlying biological motion detection. Proc Natl Acad Sci. 2018:115:E12034–E12042. 30514816 10.1073/pnas.1812859115 PMC6305007

[ref133] Spiridon M , Fischl B, Kanwisher N. Location and spatial profile of category-specific regions in human extrastriate cortex. Hum Brain Mapp. 2006:27:77–89. 15966002 10.1002/hbm.20169 PMC3264054

[ref134] Stefanatos GA , Gershkoff A, Madigan S. On pure word deafness, temporal processing, and the left hemisphere. J Int Neuropsychol Soc. 2005:11:456–470. 16209426

[ref135] Szucs D , Ioannidis JPA. Sample size evolution in neuroimaging research: an evaluation of highly-cited studies (1990–2012) and of latest practices (2017–2018) in high-impact journals. NeuroImage. 2020:221:117164. 32679253 10.1016/j.neuroimage.2020.117164

[ref136] Taylor JC , Wiggett AJ, Downing PE. Functional MRI analysis of body and body part representations in the extrastriate and fusiform body areas. J Neurophysiol. 2007:98:1626–1633. 17596425 10.1152/jn.00012.2007

[ref137] Thompson J , Parasuraman R. Attention, biological motion, and action recognition. NeuroImage, Neuroergonomics: The human brain in action and at work. 2012:59:4–13. 10.1016/j.neuroimage.2011.05.044 21640836

[ref138] Tranel D , Kemmerer D, Adolphs R, Damasio H, Damasio AR. Neural correlates of conceptual knowledge for actions. Cogn Neuropsychol. 2003:20:409–432. 20957578 10.1080/02643290244000248

[ref139] Turkeltaub PE , Eden GF, Jones KM, Zeffiro TA. Meta-analysis of the functional neuroanatomy of single-word reading: method and validation. NeuroImage. 2002:16:765–780. 12169260 10.1006/nimg.2002.1131

[ref140] Tzourio-Mazoyer N , Landeau B, Papathanassiou D, Crivello F, Etard O, Delcroix N, Mazoyer B, Joliot M. Automated anatomical labeling of activations in SPM using a macroscopic anatomical parcellation of the MNI MRI single-subject brain. NeuroImage. 2002:15:273–289. 11771995 10.1006/nimg.2001.0978

[ref141] Vaina LM , Solomon J, Chowdhury S, Sinha P, Belliveau JW. Functional neuroanatomy of biological motion perception in humans. Proc Natl Acad Sci. 2001:98:11656–11661. 11553776 10.1073/pnas.191374198 PMC58785

[ref142] Vigneau M , Beaucousin V, Hervé PY, Duffau H, Crivello F, Houdé O, Mazoyer B, Tzourio-Mazoyer N. Meta-analyzing left hemisphere language areas: Phonology, semantics, and sentence processing. NeuroImage. 2006:30:1414–1432. 16413796 10.1016/j.neuroimage.2005.11.002

[ref143] Visser M , Jefferies E, Lambon Ralph MA. Semantic processing in the anterior temporal lobes: a meta-analysis of the functional neuroimaging literature. J Cogn Neurosci. 2009:22:1083–1094. 10.1162/jocn.2009.21309 19583477

[ref144] Whatmough C , Chertkow H, Murtha S, Hanratty K. Dissociable brain regions process object meaning and object structure during picture naming. Neuropsychologia. 2002:40:174–186. 11640940 10.1016/s0028-3932(01)00083-5

[ref145] Xu J , Lyu H, Li T, Xu Z, Fu X, Jia F, Wang J, Hu Q. Delineating functional segregations of the human middle temporal gyrus with resting-state functional connectivity and coactivation patterns. Hum Brain Mapp. 2019:(18):5159–5171. 31423713 10.1002/hbm.24763 PMC6865466

[ref146] Yang J , Small SL. Language processing, functional magnetic resonance imaging of. In: Wright JD, editors. International encyclopedia of the social & behavioral sciences. Second ed. Oxford: Elsevier; 2015. pp. 368–380

[ref147] Yang DY-J , Rosenblau G, Keifer C, Pelphrey KA. An integrative neural model of social perception, action observation, and theory of mind. Neurosci Biobehav Rev. 2015:51:263–275. 25660957 10.1016/j.neubiorev.2015.01.020 PMC4940188

[ref148] Yarkoni T , Poldrack RA, Nichols TE, Van Essen DC, Wager TD. Large-scale automated synthesis of human functional neuroimaging data. Nat Methods. 2011:8:665–670. 21706013 10.1038/nmeth.1635 PMC3146590

[ref149] Yeshurun Y , Nguyen M, Hasson U. Amplification of local changes along the timescale processing hierarchy. Proc Natl Acad Sci. 2017:114:9475–9480. 28811367 10.1073/pnas.1701652114 PMC5584410

[ref150] Zaitchik D . When representations conflict with reality: The preschooler’s problem with false beliefs and “false” photographs. Cognition. 1990:35:41–68. 2340712 10.1016/0010-0277(90)90036-j

