# Supplementary material for: The cross-domain functional organization of posterior lateral temporal cortex: insights from ALE meta-analyses of 7 cognitive domains spanning 12,000 participants
Source: Cereb Cortex. 2022 Oct 20;33(8):4990–5006. doi: 10.1093/cercor/bhac394 (PMC10110446; doi:10.1093/cercor/bhac394)
Supplement: Final_Supplementary_Hodgson_bhac394 [file final_supplementary_hodgson_bhac394.zip › Final_Supplementary_Hodgson_bhac394.docx]

**Supplementary Materials for**

**The cross-domain functional organisation of posterior lateral temporal cortex: Insights from ALE meta-analyses of seven cognitive domains spanning 12000 participants**

**Supplemental Materials 1**

Code used for activation likelihood estimation analysis:

java -cp /C:/gingerale/v3.0.2/GingerALE.jar org.brainmap.meta.getALE2 /C:/domain.txt -mask=/C:/mask.nii -p=.001 -perm=10000 -clust=.001 -nonadd

Code used for contrast analysis:

java -cp /imaging/local/software/gingerale/v3.0.2/GingerALE.jar org.brainmap.meta.getALE2Contrast /C:/domain1_p001_C001_10k_ALE /C:/domain2_p001_C001_10k_ALE /C:/domain1anddomain2_p001_C001_10k_ALE -mask=/C:/mask.nii
-out1=domain1 -out2=domain2 -p=.001 -perm=10000 -minVol=20


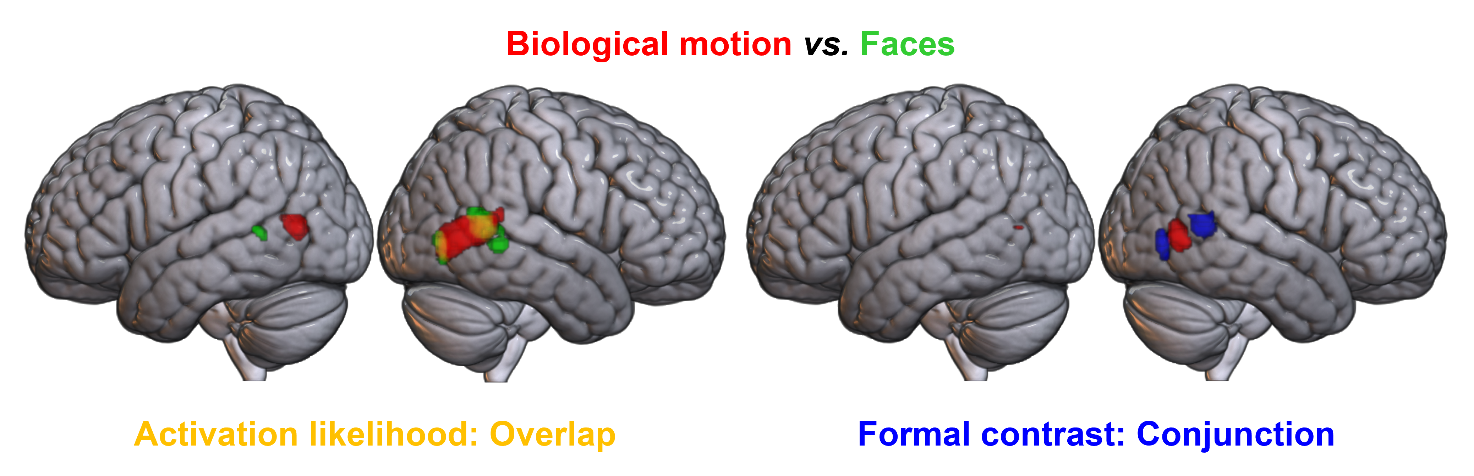


**Supplementary Figure 1.** Formal contrast analyses between the biological motion and faces domains, at a voxel-level threshold of p<.001. Left: overlays of ALE maps, showing biological motion in red and faces in green, with overlap in yellow. Right: results of formal contrast and conjunction analyses; biological motion > faces in red, faces > biological motion in green, and conjunction in blue.


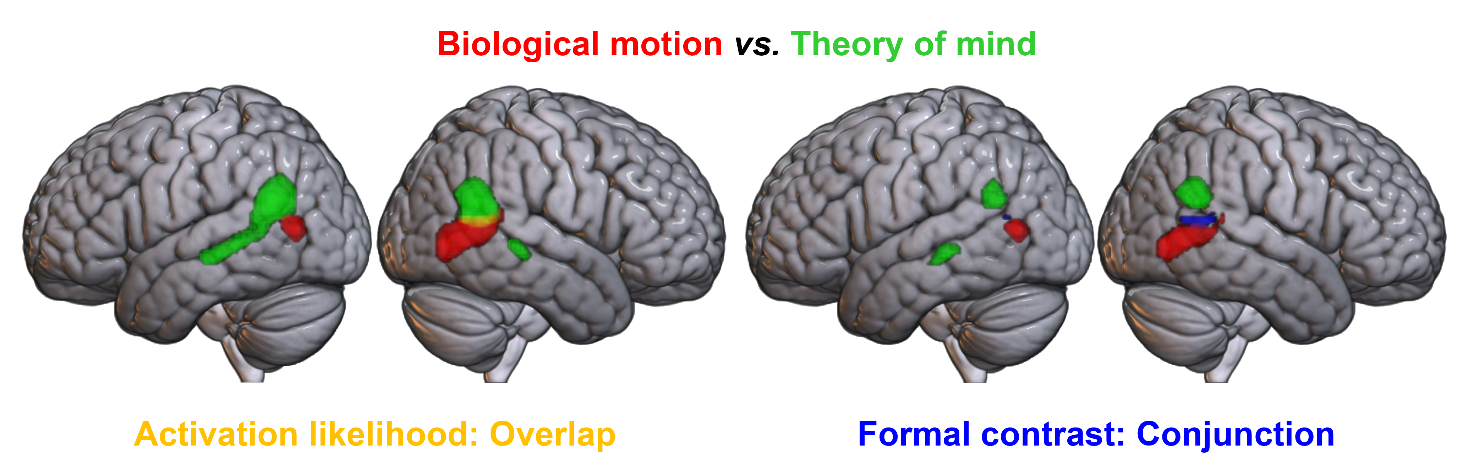


**Supplementary Figure 2.** Formal contrast analyses between the biological motion and theory of mind domains, at a voxel-level threshold of p<.001. Left: overlays of ALE maps, showing biological motion in red and theory of mind in green, with overlap in yellow. Right: results of formal contrast and conjunction analyses; biological motion > theory of mind in red, theory of mind > biological motion in green, and conjunction in blue.


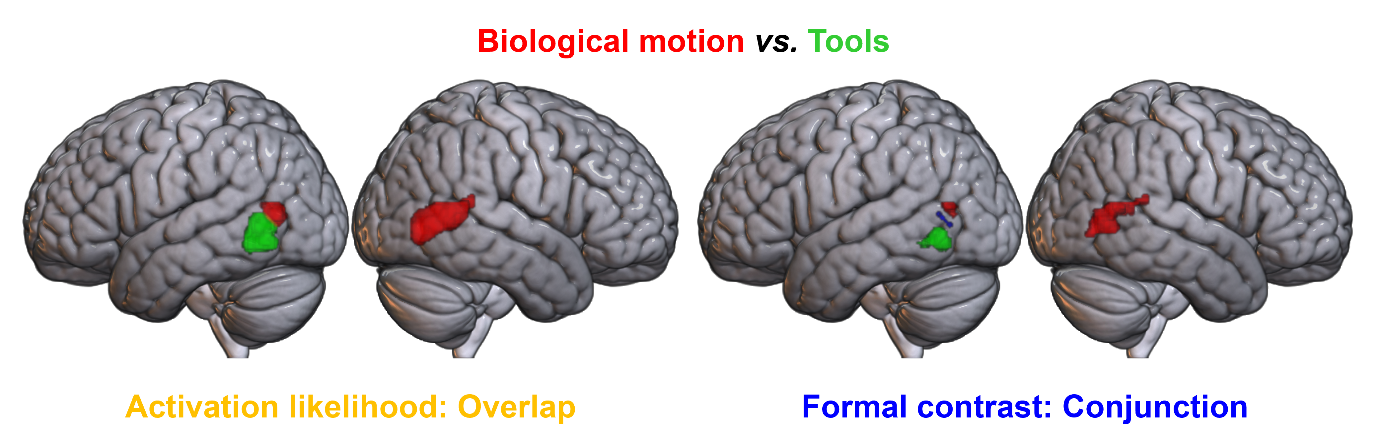


**Supplementary Figure 3.** Formal contrast analyses between the biological motion and tools domains, at a voxel-level threshold of p<.001. Left: overlays of ALE maps, showing biological motion in red and tools in green, with overlap in yellow. Right: results of formal contrast and conjunction analyses; biological motion > tools in red, tools > biological motion in green, and conjunction in blue.

**
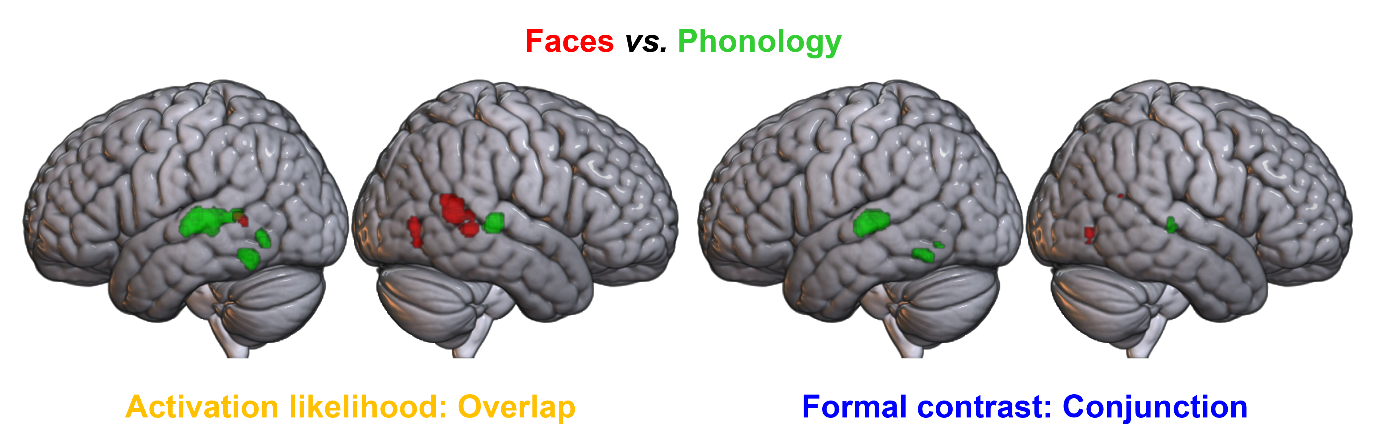
**

**Supplementary Figure 4.** Formal contrast analyses between the faces and phonology domains, at a voxel-level threshold of p<.001. Left: overlays of ALE maps, showing faces in red and phonology in green, with overlap in yellow. Right: results of formal contrast and conjunction analyses; faces > phonology in red, phonology > faces in green, and conjunction in blue.

**
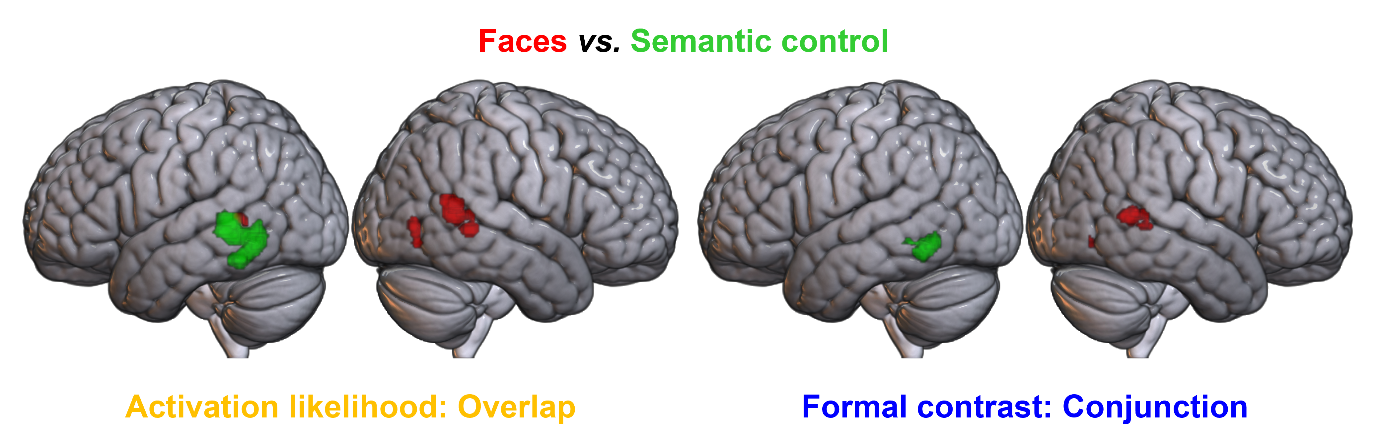
**

**Supplementary Figure 5.** Formal contrast analyses between the faces and semantic control domains, at a voxel-level threshold of p<.001. Left: overlays of ALE maps, showing faces in red and semantic control in green, with overlap in yellow. Right: results of formal contrast and conjunction analyses; faces > semantic control in red, semantic control > faces in green, and conjunction in blue.

**
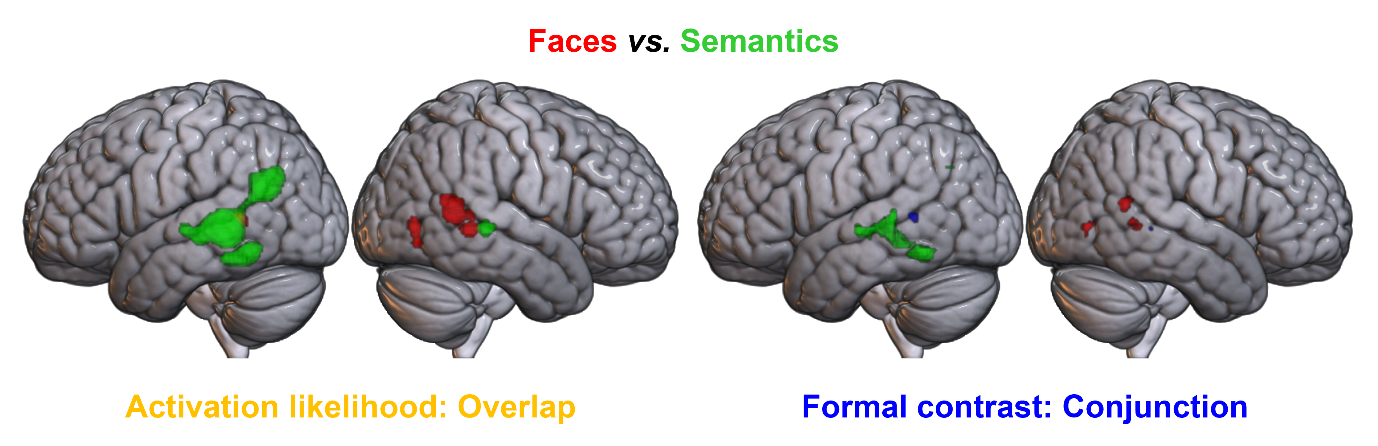
**

**Supplementary Figure 6.** Formal contrast analyses between the faces and semantics domains, at a voxel-level threshold of p<.001. Left: overlays of ALE maps, showing faces in red and semantics in green, with overlap in yellow. Right: results of formal contrast and conjunction analyses; faces > semantics in red, semantics > faces in green, and conjunction in blue.

**
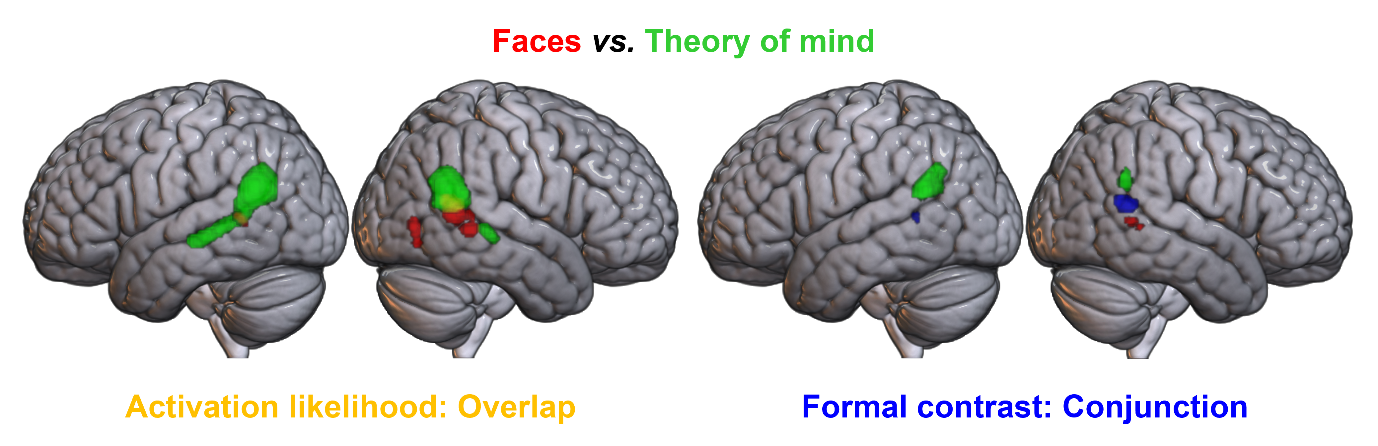
**

**Supplementary Figure 7.** Formal contrast analyses between the faces and theory of mind domains, at a voxel-level threshold of p<.001. Left: overlays of ALE maps, showing faces in red and theory of mind in green, with overlap in yellow. Right: results of formal contrast and conjunction analyses; faces > theory of mind in red, theory of mind > faces in green, and conjunction in blue.

**
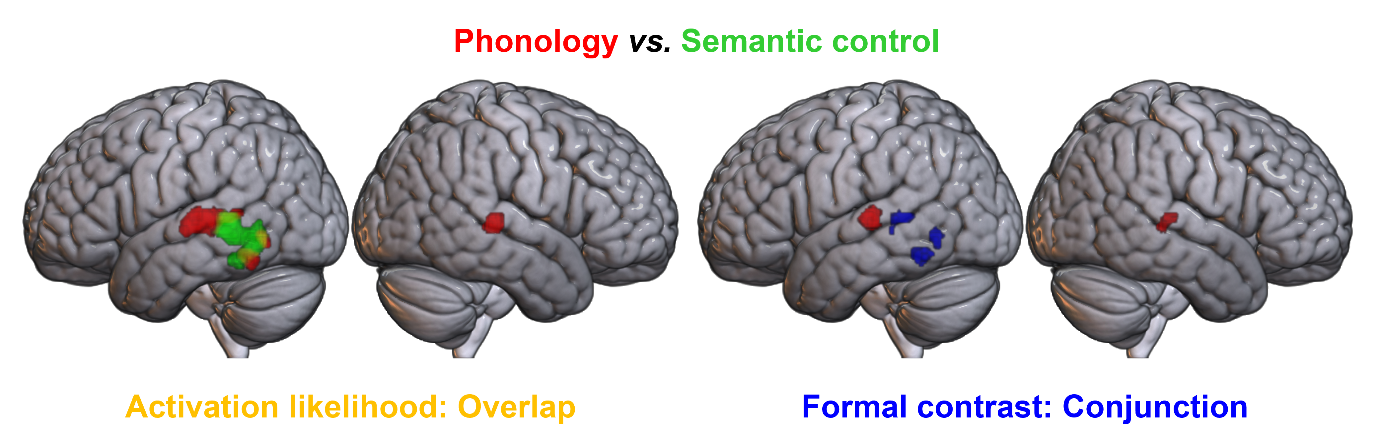
**

**Supplementary Figure 8.** Formal contrast analyses between the phonology and semantic control domains, at a voxel-level threshold of p<.001. Left: overlays of ALE maps, showing phonology in red and semantic control in green, with overlap in yellow. Right: results of formal contrast and conjunction analyses; phonology > semantic control in red, semantic control > phonology in green, and conjunction in blue.

**
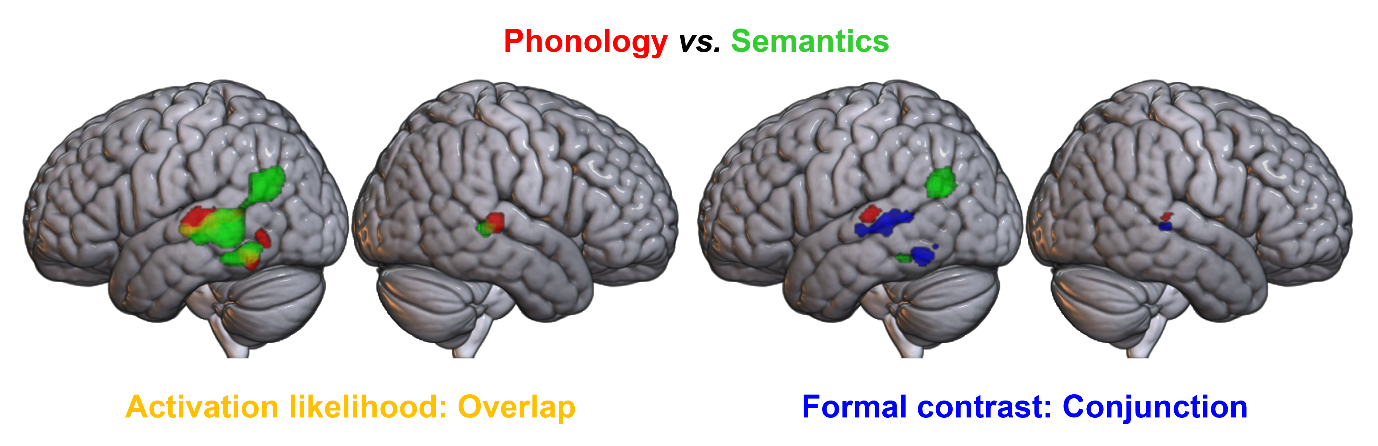
**

**Supplementary Figure 9.** Formal contrast analyses between the phonology and semantics domains, at a voxel-level threshold of p<.001. Left: overlays of ALE maps, showing phonology in red and semantics in green, with overlap in yellow. Right: results of formal contrast and conjunction analyses; phonology > semantics in red, semantics > phonology in green, and conjunction in blue.

**
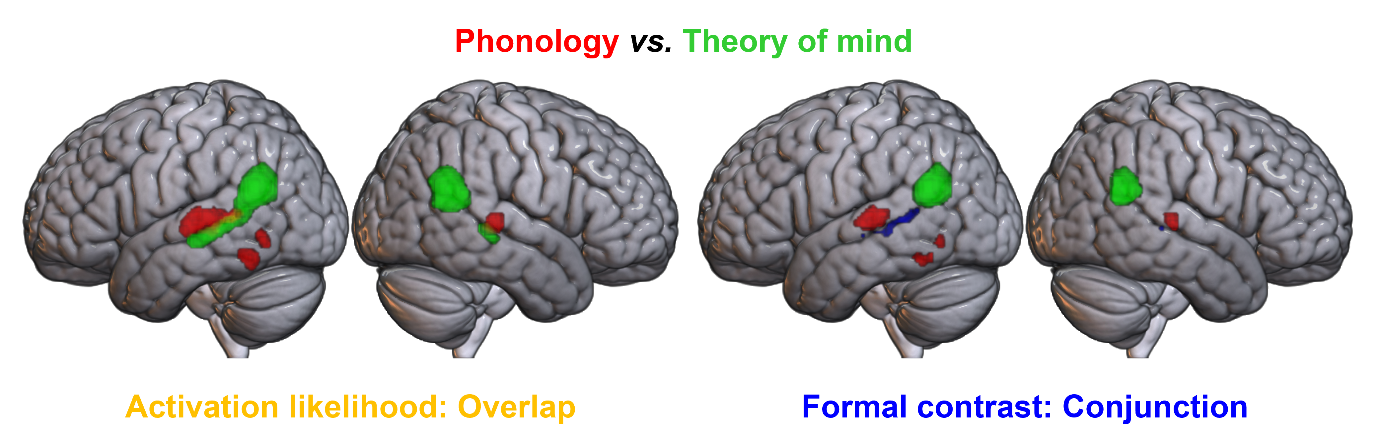
**

**Supplementary Figure 10.** Formal contrast analyses between the phonology and theory of mind domains, at a voxel-level threshold of p<.001. Left: overlays of ALE maps, showing phonology in red and theory of mind in green, with overlap in yellow. Right: results of formal contrast and conjunction analyses; phonology > theory of mind in red, theory of mind > phonology in green, and conjunction in blue.

**
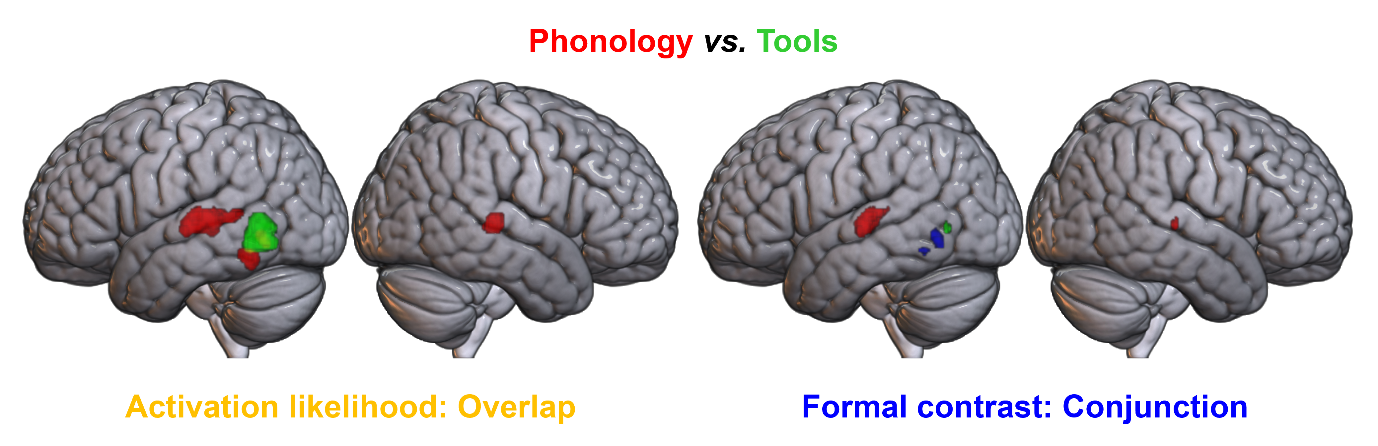
**

**Supplementary Figure 11.** Formal contrast analyses between the phonology and tools domains, at a voxel-level threshold of p<.001. Left: overlays of ALE maps, showing phonology in red and tools in green, with overlap in yellow. Right: results of formal contrast and conjunction analyses; phonology > tools in red, tools > phonology in green, and conjunction in blue.

**
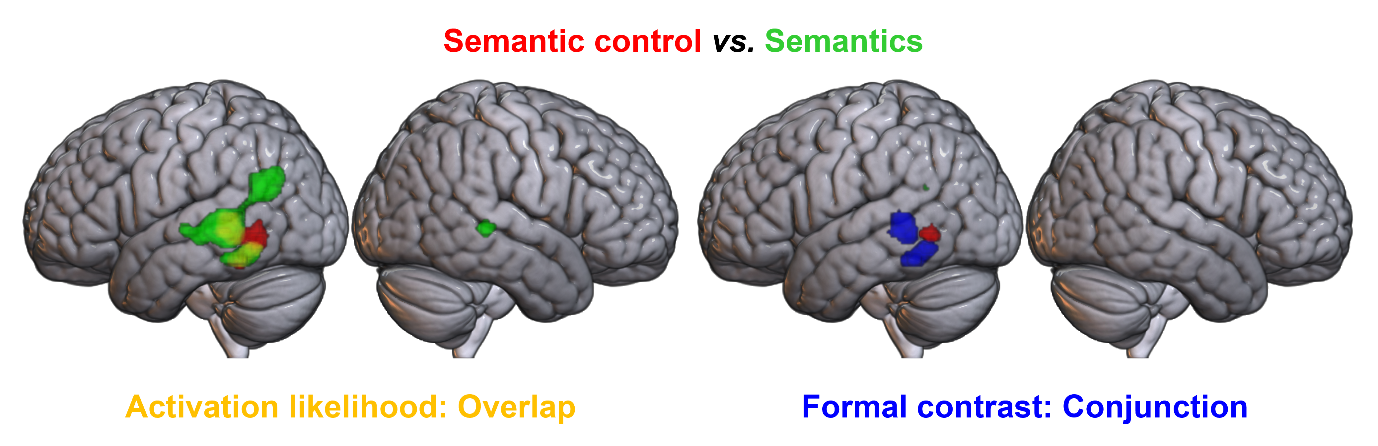
**

**Supplementary Figure 12.** Formal contrast analyses between the semantic control and semantics domains, at a voxel-level threshold of p<.001. Left: overlays of ALE maps, showing semantic control in red and semantics in green, with overlap in yellow. Right: results of formal contrast and conjunction analyses; semantic control > semantics in red, semantics > semantic control in green, and conjunction in blue.

**
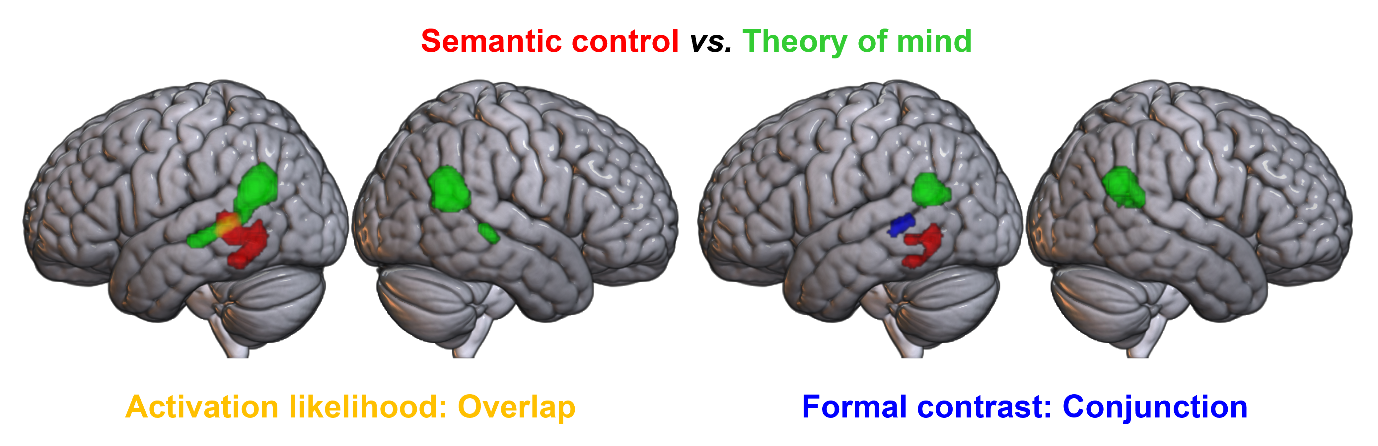
**

**Supplementary Figure 13.** Formal contrast analyses between the semantic control and theory of mind domains, at a voxel-level threshold of p<.001. Left: overlays of ALE maps, showing semantic control in red and theory of mind in green, with overlap in yellow. Right: results of formal contrast and conjunction analyses; semantic control > theory of mind in red, theory of mind > semantic control in green, and conjunction in blue.

**
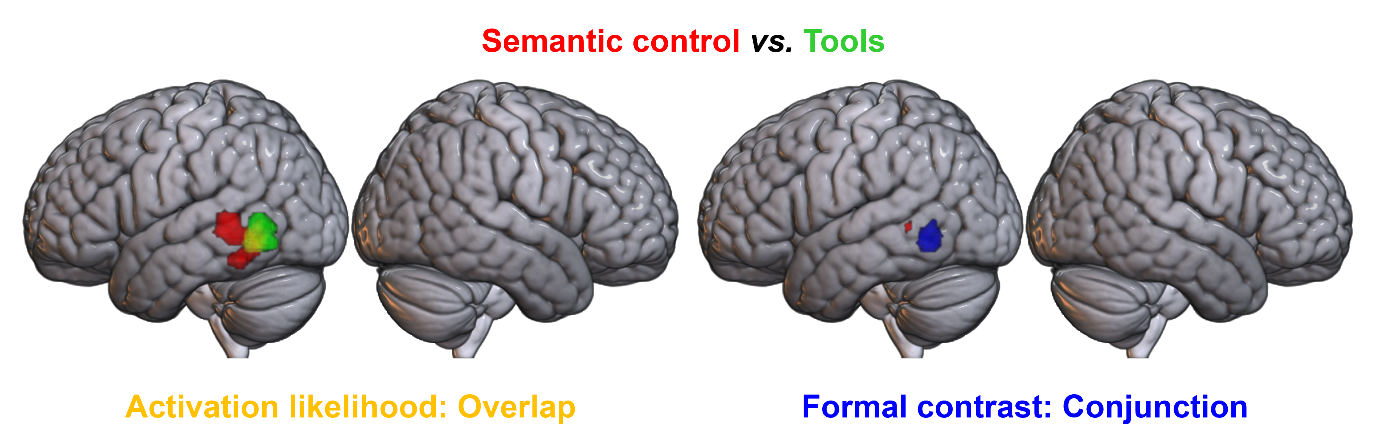
**

**Supplementary Figure 14.** Formal contrast analyses between the semantic control and tools domains, at a voxel-level threshold of p<.001. Left: overlays of ALE maps, showing semantic control in red and tools in green, with overlap in yellow. Right: results of formal contrast and conjunction analyses; semantic control > tools in red, tools > semantic control in green, and conjunction in blue.

**
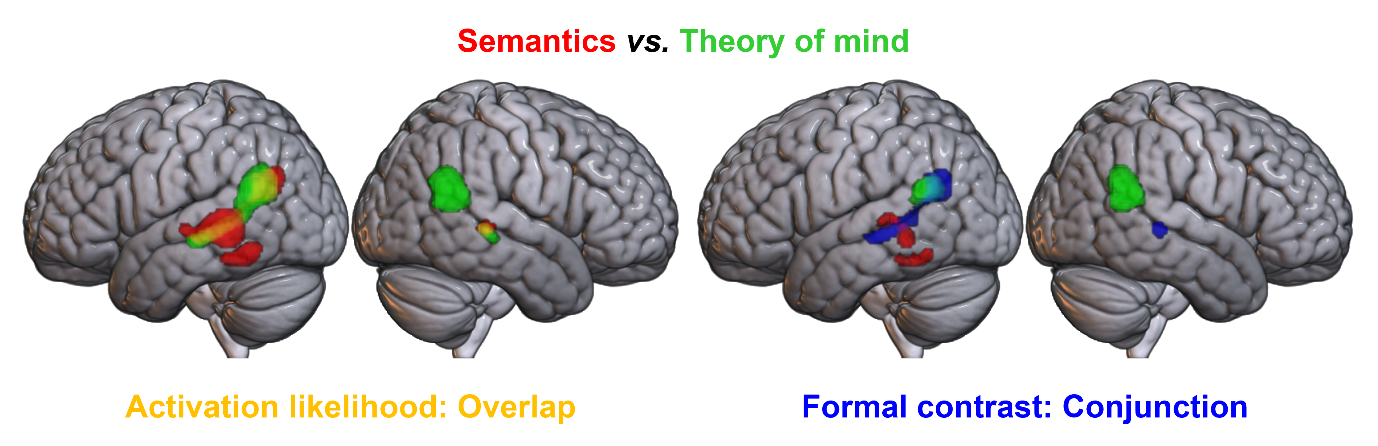
**

**Supplementary Figure 15.** Formal contrast analyses between the semantics and theory of mind domains, at a voxel-level threshold of p<.001. Left: overlays of ALE maps, showing semantics in red and theory of mind in green, with overlap in yellow. Right: results of formal contrast and conjunction analyses; semantics > theory of mind in red, theory of mind > semantics in green, and conjunction in blue.

**
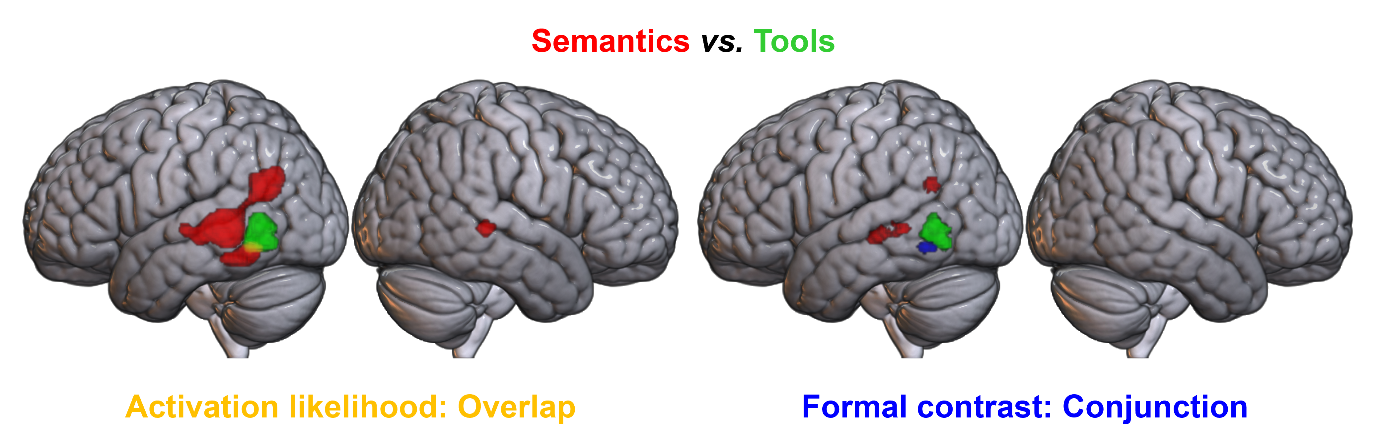
**

**Supplementary Figure 16.** Formal contrast analyses between the semantics and tools domains, at a voxel-level threshold of p<.001. Left: overlays of ALE maps, showing semantics in red and tools in green, with overlap in yellow. Right: results of formal contrast and conjunction analyses; semantics > tools in red, tools > semantics in green, and conjunction in blue.

**
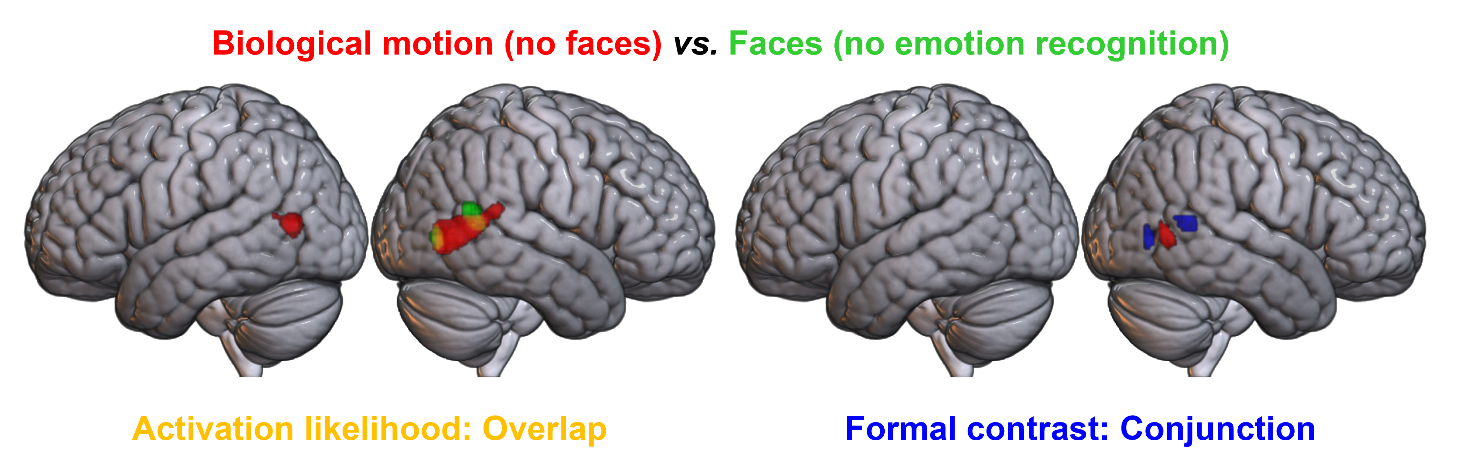
**

**Supplementary Figure 17.** Formal contrast analyses between the biological motion (no faces) and faces (no explicit recognition) reduced domains, at a voxel-level threshold of p<.001. Left: overlays of ALE maps, showing biological motion in red and faces in green, with overlap in yellow. Right: results of formal contrast and conjunction analyses; biological motion > faces in red, faces > biological motion in green, and conjunction in blue.

**
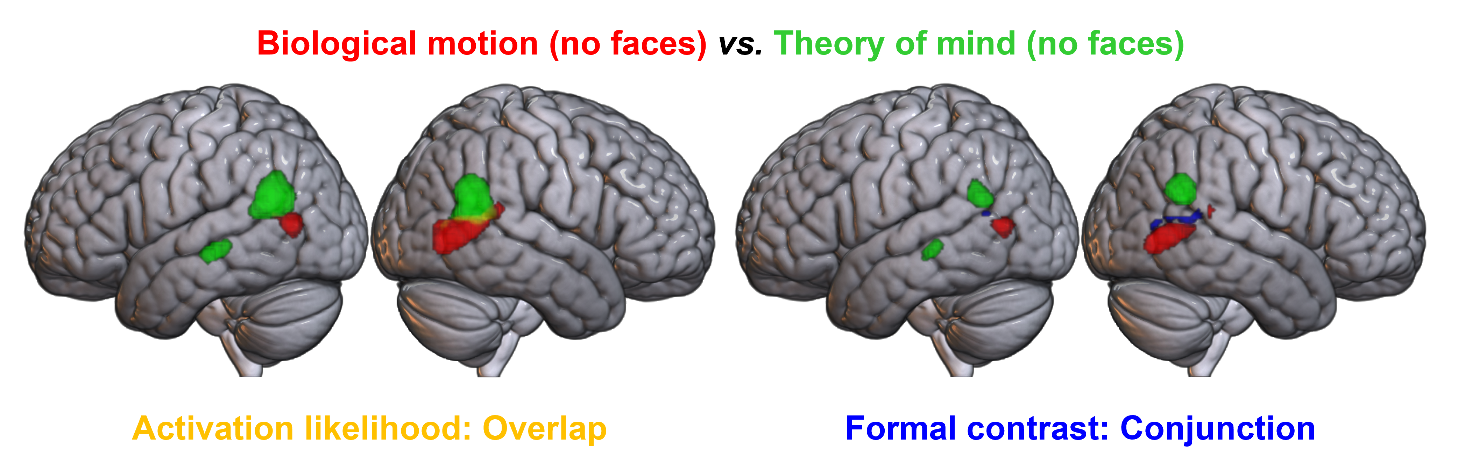
**

**Supplementary Figure 18.** Formal contrast analyses between the biological motion (no faces) and theory of mind (no faces) reduced domains, at a voxel-level threshold of p<.001. Left: overlays of ALE maps, showing biological motion in red and theory of mind in green, with overlap in yellow. Right: results of formal contrast and conjunction analyses; biological motion > theory of mind in red, theory of mind > biological motion in green, and conjunction in blue.

**
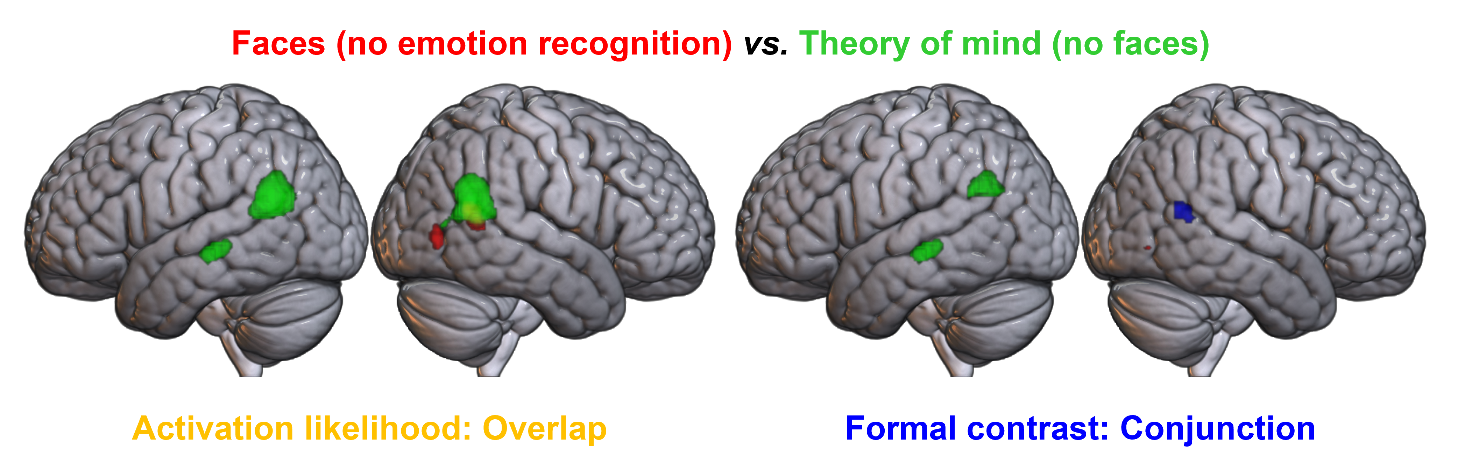
**

**Supplementary Figure 19.** Formal contrast analyses between the faces (no explicit emotion recognition) and theory of mind (no faces) reduced domains, at a voxel-level threshold of p<.001. Left: overlays of ALE maps, showing faces in red and theory of mind in green, with overlap in yellow. Right: results of formal contrast and conjunction analyses; faces > theory of mind in red, theory of mind > faces in green, and conjunction in blue.

**Supplementary Table 1:** Experiments included in biological motion dataset

| **BIOLOGICAL MOTION** | | | | |
| --- | --- | --- | --- | --- |
| **Author & Year** | **DOI** | **N** | **Foci** | **Task contrast(s)** |
| **Experiments included in full dataset and subset without faces** | | | | |
| Bonda et al. 1996 | <https://doi.org/10.1523/JNEUROSCI.16-11-03737.1996> | 11 | 5 | Point light displays (dancing) > random motion (dots); passive observation for recognition afterwards |
| Cross et al. 2006 | <https://doi.org/10.1016/j.neuroimage.2006.01.033> | 10 | 6 | Observing dance movements, while rating own ability to perform them > fixation |
| Grezes et al. 2001 | <https://doi.org/10.1006/nimg.2000.0740> | 10 | 1 | Passive observation of point light walker > rotating cube |
| Grezes et al. 2007 | <https://doi.org/10.1016/j.neuroimage.2006.11.030> | 16 | 8 | Observe videos of people opening doors > static frames taken from videos; detect oddball (upside down) stimulus |
| Grossman & Blake 2002 | <https://doi.org/10.1016/S0896-6273(02)00897-8> | 10 | 6 | Passive observation of point light display walker > scrambled walker |
| Howard et al. 1996 | <https://doi.org/10.1016/S0960-9822(02)00646-2> | 6 | 1 | Passive observation of point light display running > random motion (dots) |
| Iseki et al. 2008 | <https://doi.org/10.1016/j.neuroimage.2008.03.010> | 16 | 4 | Passive observation videos of normal gait movements > movie of scrambled frames |
| Jung et al. 2009 | <https://doi.org/10.1007/s00406-008-0833-8> | 9 | 9 | Point light displays (multiple activities) > scrambled point light animations; performing 1-back task throughout |
| Michels et al. 2005 | [10.1097/00001756-200507130-00002](https://doi.org/10.1097/00001756-200507130-00002) | 4 | 5 | Point light displays; detect human figure > detect luminance changes in stationary dots |
| Peelen et al. 2006 | <https://doi.org/10.1016/j.neuron.2006.02.004> | 17 | 4 | Animations > scrambled images of animations; 1-back task |
| Peuskens et al. 2005 | <https://doi.org/10.1111/j.1460-9568.2005.04106.x> | 6 | 4 | Observe point light displays > scrambled animations |
| Pichon et al. 2008 | <https://doi.org/10.1080/17470910701394368> | 16 | 4 | Video of actor opening/closing door > static video frames; detect oddball stimulus |
| Santi et al. 2003 | <https://doi.org/10.1162/089892903322370726> | 10 | 5 | Observe point light display (jumping/walking) > scrambled point light display |
| Saygin et al. 2004 | <https://doi.org/10.1523/JNEUROSCI.0504-04.2004> | 12 | 4 | Point light display (multiple actions) > scrambled point light display; detect colour of dots |
| Thompson et al. 2005 | <https://doi.org/10.1523/JNEUROSCI.2129-05.2005> | 10 | 5 | Animated mannequins > static mannequin body parts; detect changes in gait |
| Zentgraf et al. 2005 | <https://doi.org/10.1016/j.neuroimage.2005.02.015> | 10 | 1 | Videos of actors performing gymnastic movements > blank screen; observe to evaluate or imagine movement |
| Vaina et al. 2001 | <https://doi.org/10.1073/pnas.191374198> | 5 | 3 | Passive observation of point light display > scrambled display |
| Cunnington et al. 2006 | <https://doi.org/10.1016/j.neuroimage.2005.09.028> | 14 | 4 | Finger movements > scrambled image, observe to imitate |
| Gallagher & Frith 2004 | <https://doi.org/10.1016/j.neuropsychologia.2004.05.006> | 12 | 2 | Passive observation of hand movements > still frame of neutral hand gesture |
| Grezes et al. 1998 | <https://doi.org/10.1080/026432998381023> | 10 | 3 | Passive observation of meaningless hand movements > static hand |
| Grezes et al. 1999 | <https://doi.org/10.1093/brain/122.10.1875> | 9 | 3 | Passive observation of meaningless hand movements > static hand |
| Holle et al. 2010 | <https://doi.org/10.1016/j.neuroimage.2009.08.058> | 16 | 1 | Passive observation of hand gestures > fixation |
| Jonas et al. 2007 | <https://doi.org/10.1016/j.neuroimage.2007.03.028> | 17 | 1 | Passive observation of finger movements > static hand |
| Lui et al. 2008 | <https://doi.org/10.1080/17470910701458551> | 16 | 4 | Hand movements > blue screen, observe to imagine |
| Montgomery et al. 2007 | <https://doi.org/10.1093/scan/nsm004> | 14 | 5 | Passive observation of hand gestures > blank screen |
| Villarreal et al. 2008 | <https://doi.org/10.1016/j.neuropsychologia.2008.03.004> | 17 | 3 | Arm/hand movements > fixation, observe to recognise later |
| Wheaton et al. 2004 | <https://doi.org/10.1016/j.neuroimage.2003.12.043> | 12 | 5 | Passive observation of hand moving > static hand; passive observation of leg moving > static leg |
| Biagi et al. 2010 | <https://doi.org/10.1016/j.brainresbull.2009.12.002> | 12 | 5 | Passive observation of hand grasping object (ball/cup) > static hand |
| Chong et al. 2008 | <https://doi.org/10.1016/j.neuroimage.2007.11.030> | 15 | 6 | Hand reaching/grasping (discriminate grip type) > diamond shapes (discriminate colour) |
| Filimon et al. 2007 | <https://doi.org/10.1016/j.neuroimage.2007.06.008> | 15 | 3 | Passive observation of hand grasping abstract object > stationary object |
| Gazzola et al. 2007 | <https://doi.org/10.1016/j.neuroimage.2007.02.003> | 16 | 2 | Passive observation of hand grasping object > static hand & object |
| Grafton et al. 1996 | <https://doi.org/10.1007/BF00227183> | 7 | 2 | Passive observation of hand grasping object > static hand holding object |
| Meister & Iacoboni 2007 | [10.1371/journal.pone.0000891](https://dx.doi.org/10.1371%2Fjournal.pone.0000891) | 14 | 3 | Hand manipulating object > blank screen; count number of fingers touching the object |
| Molnar-Szakacs et al. 2006 | <https://doi.org/10.1016/j.neuroimage.2006.07.035> | 12 | 7 | Observation of hand manipulating object > blank screen |
| Perani et al. 2001 | <https://doi.org/10.1006/nimg.2001.0872> | 8 | 5 | Passive observation of hand grasping geometric object > stationary object |
| Shmuelof et al. 2005 | <https://doi.org/10.1016/j.neuron.2005.06.034> | 11 | 2 | Passive observation of hand grasping object |
| Schubotz & Von Cramon 2004 | <https://doi.org/10.1523/JNEUROSCI.1169-04.2004> | 18 | 3 | Two-handed actions > movie of changing objects; observe to judge efficacy afterwards |
| Schubotz & Von Cramon 2009 | <https://doi.org/10.1162/jocn.2009.21049> | 18 | 3 | Passive observation of two handed-actions > blank screen |
| Tai et al. 2004 | <https://doi.org/10.1016/j.cub.2004.01.005> | 7 | 2 | Passive observation of human hand grasping object > static hand; passive observation of robot hand grasping object > static hand |
| Turella et al. 2009 | <https://doi.org/10.1016/j.neuroimage.2009.03.002> | 16 | 2 | Passive observation of hand reaching/grasping object > static arm & object |
| **Experiments included in the full dataset but not in the subset without faces** | | | | |
| Fox et al. 2009 | <https://doi.org/10.1002/hbm.20630> | 16 | 4 | Moving faces > moving objects; 1-back task |
| Kilts et al. 2003 | <https://doi.org/10.1006/nimg.2002.1323> | 13 | 2 | Dynamic faces > static faces; judge physical position of face relative to centre |
| Lee et al. 2009 | <https://doi.org/10.1016/j.neuropsychologia.2009.10.005> | 17 | 4 | Head turning motion > movie of scrambled frames; observe and detect red dot stimulus |
| Hooker et al. 2003 | <https://doi.org/10.1016/S0926-6410(03)00143-5> | 10 | 4 | Video of moving gaze > moving arrow on static face; monitor target of eye gaze |
| Pelphrey et al. 2005 | <https://doi.org/10.1093/cercor/bhi064> | 15 | 3 | Passive observation of animated character shifting eye gaze > still frame of character |
| Puce et al. 1998 | <https://doi.org/10.1523/JNEUROSCI.18-06-02188.1998> | 11 | 7 | Observation of eye gaze shift > static face with moving radial background |
| Pierno et al. 2006 | <https://doi.org/10.1162/jocn.2006.18.12.2130> | 14 | 9 | Passive observation of eye gaze shift > static eyes |
| Wicker et al. 1998 | <https://doi.org/10.1006/nimg.1998.0357> | 10 | 5 | Passive observation of eye gaze shift > static eyes |
| Calvert & Campbell 2003 | <https://doi.org/10.1162/089892903321107828> | 8 | 8 | Observation of lower half of face, mouth forming meaningless syllables > closed mouth images |
| Calvert et al. 1997 | <https://doi.org/10.1126/science.276.5312.593> | 7 | 7 | Lip movements > static face |
| Campbell et al. 2001 | <https://doi.org/10.1016/S0926-6410(01)00054-4> | 7 | 10 | Lip movements > static face |
| Paulesu et al. 2003 | <https://doi.org/10.1152/jn.00926.2002> | 8 | 7 | Observation of backward video of face speaking > static face |
| Thompson et al. 2007 | <https://doi.org/10.1016/j.neuroimage.2007.05.058> | 11 | 2 | Passive observation of animated face opening mouth > radial grating motion |

**Supplementary Table 2:** Experiments included in faces dataset

| **FACES** | | | | |
| --- | --- | --- | --- | --- |
| **Author & Year** | **DOI** | **N** | **Foci** | **Task contrast(s)** |
| **Experiments included in full dataset and subset without explicit emotion recognition** | | | | |
| Hessl et al. 2007 | <https://doi.org/10.1093/brain/awl338> | 13 | 3 | Passive viewing of fearful faces > scrambled faces |
| Andersson et al. 2008 | <https://doi.org/10.1016/j.biopsych.2007.02.022> | 16 | 2 | Neutral faces > houses, face discrimination |
| Bird et al. 2006 | <https://doi.org/10.1016/j.neuroimage.2006.02.037> | 16 | 2 | Passive viewing of neutral & fearful Ekman faces > houses |
| Iidaka et al. 2001 | <https://doi.org/10.1162/089892901753294338> | 12 | 1 | Faces > rectangles; gender judgement |
| Iidaka et al. 2002 | <https://doi.org/10.1002/hipo.1113> | 12 | 1 | Faces > rectangles; gender judgement |
| Rossion et al. 2012 | <https://doi.org/10.1016/j.bandc.2012.01.001> | 40 | 4 | Photographs of faces > scrambled faces; photographs of faces > cars photos; 1-back identity matching |
| Slotnik et al. 2013 | <https://doi.org/10.1016/j.neuroimage.2013.06.032> | 12 | 3 | Faces > common objects; pleasantness judgement task |
| Johnston et al. 2005 | <https://doi.org/10.1111/j.1460-9568.2005.04294.x> | 10 | 2 | Faces > shapes; gender judgement |
| Maurer et al. 2007 | <https://doi.org/10.1016/j.neuropsychologia.2006.11.016> | 12 | 1 | Passive viewing of greyscale faces > objects/houses |
| Pourtois et al. 2005a | <https://doi.org/10.1162/0898929054475181> | 14 | 3 | Faces > houses; 1-back identity matching |
| Ramirez-Ruiz et al. 2008 | <https://doi.org/10.1002/mds.22258> | 10 | 5 | Colour photos of faces > patterns; 1-back identity matching |
| Bukowski et al. 2013 | <https://doi.org/10.1016/j.cortex.2013.05.002> | 11 | 2 | Pictures of faces > cars/scrambled faces; 1-back identity matching |
| Ethofer et al. 2013 | <https://doi.org/10.1016/j.neuroimage.2013.02.064> | 23 | 2 | Faces > houses/objects/scenes; 1-back identity matching |
| Pegors et al. 2015 | <https://doi.org/10.1162/jocn_a_00777> | 28 | 2 | Colour photos of faces > photos of places/natural scenes; attractiveness rating |
| Koppe et al. 2015 | <https://doi.org/10.1016/j.neuroimage.2015.06.081> | 30 | 2 | Angry/happy faces > geometric figures; gender judgement |
| Opmeer et al. 2016 | <https://doi.org/10.1002/da.22425> | 32 | 1 | Colour photos, various emotions > scrambled faces; gender judgement |
| Frässle et al. 2016 | <https://doi.org/10.1016/j.neuroimage.2015.09.055> | 20 | 2 | Passive viewing of greyscale neutral faces > scrambled images |
| Schultz et al. 2009 | <https://doi.org/10.1007/s00221-009-1721-9> | 10 | 1 | Surprised/angry faces > scrambled faces; 1-back identity matching |
| Bonner-Jackson et al. 2005 | <https://doi.org/10.1016/j.biopsych.2005.05.011> | 26 | 1 | Unfamiliar faces > visually presented words; encoding task |
| Hasson et al. 2002 | <https://doi.org/10.1016/S0896-6273(02)00662-1> | 13 | 3 | Passive viewing of greyscale faces > buildings/letter strings |
| Kranz & Ishai 2006 | <https://doi.org/10.1016/j.cub.2005.10.070> | 40 | 2 | Greyscale faces > phase-scrambled faces; attractiveness judgement |
| Pierce et al. 2004 | <https://doi.org/10.1093/brain/awh289> | 9 | 1 | Greyscale faces > unfamiliar faces; greyscale faces > fixation; gender judgement |
| Platek et al. 2006 | <https://doi.org/10.1002/hbm.20168> | 12 | 1 | Neutral familiar faces > unfamiliar faces; neutral unfamiliar faces > scrambled faces |
| Williams et al. 2005 | <https://doi.org/10.1016/j.neuroimage.2004.08.017> | 13 | 1 | Ekman faces > photos of houses; identity matching |
| **Experiments included in full dataset but not subset without explicit emotion recognition** | | | | |
| Seitz et al. 2008 | <https://doi.org/10.1016/j.neuroscience.2007.10.066> | 14 | 1 | Happy/sad Ekman faces, recognise emotion > count earrings on faces |
| Narumoto et al. 2001 | <https://doi.org/10.1016/S0926-6410(01)00053-2> | 11 | 2 | Ekman faces, emotion/identity matching > scrambled images, contour matching |
| Payer et al. 2008 | <https://doi.org/10.1016/j.drugalcdep.2007.09.009> | 12 | 3 | Ekman faces > shapes; emotion judgement |
| Fitzgerald et al. 2006 | <https://doi.org/10.1016/j.neuroimage.2005.11.003> | 20 | 1 | Photos of Ekman faces > radios; emotion judgement |
| Kesler-West et al. 2001 | <https://doi.org/10.1016/S0926-6410(00)00073-2> | 21 | 4 | Emotional & neutral faces > scrambled faces; emotion judgement |
| Scheuerecker et al. 2007 | <https://doi.org/10.1159/000110726> | 12 | 2 | Sad/angry faces > geometric shapes; gender/emotion matching |
| Nomi et al. 2008 | <https://doi.org/10.1186/1744-9081-4-41> | 14 | 2 | Happy/sad/neutral faces > scrambled faces; emotion judgement/empathising |
| Fakra et al. 2008 | <https://doi.org/10.1016/j.schres.2007.11.040> | 14 | 2 | Angry/fearful faces > shapes; emotion judgement |
| Herrington et al. 2011 | <https://doi.org/10.1016/j.neuroimage.2011.03.072> | 39 | 2 | Ekman faces > houses; identity matching |
| Quintana et al. 2011 | <https://doi.org/10.1016/j.pscychresns.2010.09.005> | 15 | 1 | Greyscale faces > shapes/complex patterns; emotion matching task |
| Torrisi et al. 2013 | <https://doi.org/10.1016/j.neuroimage.2013.06.025> | 52 | 7 | Greyscale Ekman faces > basic shapes; emotion judgement |
| Prochnow et al. 2013b | <https://doi.org/10.1080/17470919.2013.812536> | 12 | 1 | Greyscale Ekman faces > scrambled faces; emotion judgement |
| Prochnow et al. 2014 | <https://doi.org/10.1016/j.bandc.2014.07.004> | 26 | 1 | Ekman faces > scrambled faces; emotion judgement |
| Binelli et al. 2015 | <https://dx.doi.org/10.1503%2Fjpn.140384> | 20 | 1 | Emotional faces > shapes; emotion judgement |
| Britton et al. 2006 | <https://doi.org/10.1016/j.neuroimage.2005.12.050> | 12 | 1 | Emotional faces > fixation; emotional faces > neutral faces; judge emotion intensity |
| Wright et al. 2006 | https://doi.org/10.1016/j.neuroimage.2005.07.047 | 12 | 5 | Emotional faces > scrambled faces; emotion judgement |
| Brown et al. 2006 | https://doi.org/10.1037/1528-3542.6.2.239 | 58 | 1 | Angry/fearful faces > shapes; emotion judgement |

**Supplementary Table 3:** Experiments included in phonology dataset

| **PHONOLOGY** | | | | |
| --- | --- | --- | --- | --- |
| **Author & Year** | **DOI** | **N** | **Foci** | **Task contrast(s)** |
| Baciu et al. 2005 | <https://doi.org/10.1016/j.ejrad.2004.11.004> | 10 | 4 | Words, rhyme judgement > unreadable characters, visual judgement |
| Bohland et al. 2006 | <https://doi.org/10.1016/j.neuroimage.2006.04.173> | 13 | 1 | Syllable sequences > rest; go/no-go overt reading task |
| Booth et al. 2002 | <https://doi.org/10.1002/hbm.10054> | 13 | 1 | Auditory & visual words, rhyme > semantic judgement |
|  |  | 12 | 2 | Word rhyme judgement > lines, judge length; words rhyme judgement > tone, pitch judgement |
| Demonet et al. 1992 | <https://doi.org/10.1093/brain/115.6.1753> | 9 | 3 | Phoneme monitoring in non-words > tone detection task |
| Desai et al. 2006 | <https://doi.org/10.1162/jocn.2006.18.2.278> | 125 | 1 | Verb generation task |
| Devlin et al. 2003 | <https://doi.org/10.1162/089892903321107837> | 12 | 5 | Visual words, syllable number > semantic category judgement |
| Fiebach et al. 2007 | <https://doi.org/10.1162/jocn.2007.19.12.2035> | 12 | 1 | Phonological > semantic working memory task |
| Ghosh et al. 2008 | <https://doi.org/10.1044/1092-4388(2008/07-0119)> | 10 | 2 | Bisyllabic > monosyllabic words, covert reading task |
| Gold et al. 2005 | <https://doi.org/10.1093/cercor/bhi024> | 32 | 1 | Regularise irregular words > generate category-appropriate verbs |
| Gruber et al. 2009 | <https://doi.org/10.1002/hbm.20849> | 18 | 4 | Phonological working memory > orthographical/letter case judgement |
| Katzir et al. 2005 | <https://doi.org/10.1016/j.neuroimage.2005.04.013> | 12 | 2 | Judge whether names start with same letter > visual perception of symbol grid |
| Kuo et al. 2004 | <https://doi.org/10.1016/j.neuroimage.2003.12.007> | 10 | 4 | Homophone > character form judgement; Chinese/Korean characters |
| Liu et al. 2008 | <https://doi.org/10.1162/jocn.2009.21141> | 16 | 1 | Rhyme judgement > 1-back symbol matching task; rhyme judgement > 1-back tone matching task |
| Lurito et al. 2000 | [https://doi.org/10.1002/1097-0193(200007)10:3<99::AID-HBM10>3.0.CO;2-Q](https://doi.org/10.1002/1097-0193(200007)10:3%3c99::AID-HBM10%3e3.0.CO;2-Q) | 5 | 5 | Covertly generate words beginning with given letter > fixate on symbol |
| McDermott et al. 2003 | <https://doi.org/10.1016/S0028-3932(02)00162-8> | 20 | 1 | Rhyme judgement > semantic relatedness judgement |
| Mummery et al. 1998 | <https://doi.org/10.1162/089892998563059> |  |  | Judgements about number of syllables > about colour/location |
| Price et al. 1997 | <https://doi.org/10.1162/jocn.1997.9.6.727> | 6 | 2 | Syllable number judgement > living/non-living judgement |
| Scott et al. 2003 | <https://doi.org/10.1016/S1053-8119(03)00083-1> | 9 | 2 | Judge syllable number > judge whether noun can apply to human |
| Seghier et al. 2004 | <https://doi.org/10.1002/hbm.20053> | 26 | 3 | Rhyme detection > visual perceptual categorisation |
| Specht et al. 2003 | <https://doi.org/10.1016/S0304-3940(03)00494-4> | 15 | 2 | Detect pseudoword amongst words > detect low-pitched tone |
| Tyler et al. 2005 | <https://doi.org/10.1016/j.neuropsychologia.2005.03.008> | 18 | 5 | Phonological similarity judgement > white/pink noise |
| Xu et al. 2001 | <https://doi.org/10.1093/cercor/11.3.267> | 12 | 2 | Rhyming task > colour matching task |
| Xu et al. 2002 | <https://doi.org/10.1006/nimg.2002.1215> | 6 | 3 | Rhyme judgement > bold text judgement |
| Braun et al. 1997 | <https://doi.org/10.1093/brain/120.5.761> | 20 | 2 | Paced/automatic speech > non-lexical production |
| Heim et al. 2002 | <https://doi.org/10.1016/S0304-3940(02)00494-9> | 12 | 2 | Production of random phonemes > rest |
| Price et al. 1996 | <https://doi.org/10.1093/brain/119.3.919> | 6 | 13 | Repetition > passive listening |
| Warburton et al. 1996 | <https://doi.org/10.1093/brain/119.1.159> | 6 | 5 | Silent repetition of pseudowords > rest |
| Wildgruber et al. 2001 | <https://doi.org/10.1006/nimg.2000.0672> | 10 | 1 | Silent repetition of syllable > rest |
| Calvert et al. 1999 | <https://doi.org/10.1097/00001756-199908200-00033> | 5 | 5 | Silent repetition of numbers > rest |
| Beauregard et al. 1997 | <https://doi.org/10.1162/jocn.1997.9.4.441> | 10 | 2 | Passive viewing of letters > rest |
| Joanisse & Gati 2003 | <https://doi.org/10.1016/S1053-8119(03)00046-6> | 7 | 3 | Phonemes > non-speech sine waves; same/different judgement task |
| Paulesu et al. 2000 | <https://doi.org/10.1038/71163> | 78 | 6 | Read words aloud > rest |
| Poeppel et al. 2004 | <https://doi.org/10.1016/j.neuropsychologia.2003.07.010> | 10 | 9 | Phoneme categorisation > tone direction discrimination |
| Belin & Zatorre 2003 | <https://doi.org/10.1097/00001756-200311140-00019> | 14 | 2 | Passive listening of spoken syllables > rest |
| Hugdahl et al. 2003 | <https://doi.org/10.1016/S0093-934X(02)00500-X> | 13 | 3 | Vowel vs syllable judgement > rest |
| Jancke et al. 2002 | <https://doi.org/10.1006/nimg.2001.1027> | 6 | 9 | Passive listening > rest |
| Herbster et al. 1997 | [https://doi.org/10.1002/(SICI)1097-0193(1997)5:2<84::AID-HBM2>3.0.CO;2-I](https://doi.org/10.1002/(SICI)1097-0193(1997)5:2%3c84::AID-HBM2%3e3.0.CO;2-I) | 10 | 1 | Read irregular/nonwords > respond to letter strings |
| Kotz et al. 2002 | <https://doi.org/10.1006/nimg.2002.1316> | 13 | 1 | Pseudowords > words; determine if “word” is German |
| Meyer et al. 2002 | <https://doi.org/10.1002/hbm.10042> | 14 | 2 | Syntactic > normal sentences; syntactic > prosodic sentences; active vs passive tense judgement |
| Binder et al. 2000 | <https://doi.org/10.1093/cercor/10.5.512> | 28 | 7 | Pseudowords > rest; reversed monosyllabic words > rest; passive listening |
| Cappa et al. 1998 | <https://doi.org/10.1006/nimg.1998.0368> | 13 | 2 | Detect letter “e” in pseudowords > rest |
| Fiez et al. 1996 | <https://doi.org/10.1093/cercor/6.1.1> | 3 | 2 | Passive listening of words/pseudowords > rest |
| Hickock et al. 2003 | <https://doi.org/10.1162/jocn.2003.15.5.673> | 9 | 4 | Pseudowords > rest; listen, occasionally rehearse |
| Heim et al. 2003 | <https://doi.org/10.1016/S0926-6410(02)00284-7> | 8 | 3 | Judge whether name (of line drawing) starts with specific letter > semantic judgement |
| Bunge et al. 2001 | <https://doi.org/10.1093/brain/124.10.2074> | 23 | 1 | Phonological working memory (array of letters) task |
| Paulesu et al. 1993 | <https://doi.org/10.1038/362342a0> | 6 | 4 | Working memory (letter strings); rhyme judgement > orthography |
| Rypma et al. 1999 | <https://doi.org/10.1006/nimg.1998.0404> | 6 | 7 | Working memory; uppercase letter array |
| Belin et al. 2000 | <https://doi.org/10.1038/35002078> | 8 | 4 | Passive listening of vocal sounds > non-vocal stimuli (instruments, nature sound) |
| Bouhali et al. 2019 | 10.1073/pnas.1904184116 | 20 | 1 | Pseudowords > words; lexical decision & reading aloud |
| Kwok et al. 2019 | <https://doi.org/10.1016/j.bandl.2019.104662> | 17 | 1 | Tone judgement > font size judgement |
| Kim et al. 2017 | <https://doi.org/10.1155/2017/5732642> | 24 | 3 | Phonological > logographic reading (Korean characters) |
| Hoffman et al. 2015 | <https://doi.org/10.1073/pnas.1502032112> | 26 | 4 | Real word reading > saying “ok” to letter strings |
| Tzourizo-Mazoyer et al. 2015 | 10.1007/s00429-014-0746-4 | 281 | 9 | Passive listening of overlearned words > viewing scrambled drawings |
| Salo et al. 2015 | 10.3389/fnhum.2015.00102 | 15 | 1 | Beginning letter judgement > colour of font/gender of speaker judgement |
| Rao & Singh 2015 | 10.1016/j.bandl.2014.11.010 | 14 | 2 | Naming aloud, low > high frequency words |
| Diaz et al. 2014 | 10.3389/fnhum.2014.00167 | 16 | 10 | Phonologically related > unrelated distractors; name object from line drawings |
| Hernandez et al. 2013 | 10.1111/psyp.12141 | 16 | 1 | Rhyme judgement (pairs of French words) > font judgement (pairs of unreadable sequences) |
| Danelli et al. 2013 | 10.1002/hbm.22098 | 28 | 5 | Pseudoword reading (covert) > view strings of lines; auditory rhyming > tone discrimination |
| Yoo et al. 2012 | 10.1016/j.bandl.2012.04.011 | 22 | 2 | Repetition of pseudowords > rest; perceptual judgement (word vs pseudoword) > rest |
| Peyrin et al. 2012 | 10.1016/j.bandl.2011.12.015 | 14 | 2 | Letter rhyme judgement > fixation |
| Prado et al. 2011 | 10.1002/hbm.21159 | 26 | 1 | Rhyme judgement > visual perception task |
| Perrone-Bertolotti et al. 2011 | 10.1016/j.jneuroling.2011.03.003 | 22 | 2 | Phoneme monitoring (pseudowords) > visual perception (meaningless font strings) |
| Kircher et al. 2011 | 10.1016/j.brainres.2011.03.054 | 15 | 4 | Verbal fluency, letter & rhyme > rest |

**Supplementary Table 4:** Experiments included in semantic control dataset

| **SEMANTIC CONTROL** | | | | |
| --- | --- | --- | --- | --- |
| **Author & Year** | **DOI** | **N** | **Foci** | **Task contrast(s)** |
| Willems et al. 2008 | https://doi.org/10.1162/jocn.2008.20085 | 19 | 2 | Incongruent > congruent sequences |
|  |  | 19 | 1 | Incongruent > congruent |
| Balthasar et al. 2011 | https://doi.org/10.1016/j.brainres.2011.06.054 | 18 | 2 | Homonyms > identification |
| Hsu et al. 2011 | https://doi.org/10.1162/jocn.2011.21619 | 12 | 2 | Less > more semantic distance to foils |
| Vitello et al. 2014 | https://doi.org/10.3389/fnhum.2014.00530 | 20 | 2 | Presence of ambiguous word in sentence > no ambiguous word |
|  |  | 20 | 3 | Sentence with no ambiguous words > signal correlated noise |
| Rodd et al. 2012 | https://doi.org/10.1093/cercor/bhr252 | 15 | 9 | Ambiguity across time |
|  |  | 15 | 6 | Speech > signal correlated noise |
| Tahmasebi et al. 2012 | https://doi.org/10.1093/cercor/bhr205 | 24 | 1 | Ambiguous homonym > unambiguous word |
| Bekinschtein et al. 2011 | https://doi.org/10.1523/JNEUROSCI.5058-10.2011 | 12 | 1 | Ambiguous homonym/homophone > unambiguous word |
| Tune et al. 2016 | https://doi.org/10.1523/JNEUROSCI.4100-15.2016 | 18 | 9 | Anomalous > normal sentences |
| Willems et al. 2016 | <https://doi.org/10.1093/cercor/bhv075> | 24 | 5 | Word context surprisal |
| Obleser et al. 2010 | https://doi.org/10.1093/cercor/bhp128 | 16 | 2 | Low > high cloze probability |
|  |  | 16 | 1 | High > low intelligibility |
| Scharinger et al. 2016 | https://doi.org/10.1002/hbm.23060 | 22 | 2 | Unpredictable > predictable word in incomplete > complete sentence |
| Krieger-Redwood et al. 2015 | https://doi.org/10.1016/j.neuropsychologia.2015.02.030 | 22 | 6 | Strong > weak associations between pictures |
| Schnur et al. 2009 | https://doi.org/10.1073/pnas.0805874106 | 16 | 3 | Semantic blocks > mixed (greater interference) |
| Tobia et al. 2017 | https://doi.org/10.14814/phy2.13078 | 16 | 1 | Subordinate > prototypical word use |
| Thompson Schill et al. 1997 | https://doi.org/10.1073/pnas.94.26.14792 | 6 | 2 | High > low selection |
|  |  | 6 | 1 | High > low selection |
| Bitan et al. 2017 | https://psycnet.apa.org/doi/10.1037/neu0000357 | 23 | 1 | Subordinate > dominant meaning relation |
| Abraham et al. 2012 | https://doi.org/10.1016/j.neuropsychologia.2012.04.015 | 19 | 2 | Use > location |
| Snyder et al. 2011 | https://doi.org/10.1162/jocn_a_00023 | 18 | 3 | High > low competition |
| Balthasar et al. 2011 | https://doi.org/10.1016/j.brainres.2011.06.054 | 18 | 2 | Homonyms > identification |
| Grindrod et al. 2008 | https://doi.org/10.1016/j.brainres.2008.07.017 | 15 | 1 | Lexical decision on ambiguous words |
| Madore 2019 | https://doi.org/10.1093/cercor/bhx312 | 32 | 10 | Alternative uses > associations |
| Hallam et al. 2016 | https://doi.org/10.1016/j.neuropsychologia.2016.09.012 | 18 | 1 | Weak associations/high retrieval > strong associations/low retrieval demands |
| Jeon 2012 | 10.1097/WNR.0b013e32835a19ae | 14 | 1 | Homonym > unambiguous word |
| Tylen et al. 2015 | https://doi.org/10.1016/j.neuroimage.2015.07.047 | 24 | 2 | Incoherent > coherent story |
| Mestres-Misse et al. 2014 | https://doi.org/10.1016/j.neuroimage.2014.05.002 | 23 | 3 | Subordinacy/ambiguity/incongruence |
| Newman et al. 2010 | https://doi.org/10.1016/j.bandl.2010.02.001 | 20 | 2 | Unrelated > related sentence |
| Peelle et al. 2009 | https://doi.org/10.1016/j.neuropsychologia.2008.10.027 | 25 | 2 | Inconsistent > consistence description |
| Zhu et al. 2012 | https://doi.org/10.1016/j.neuroimage.2012.02.036 | 27 | 2 | Incongruence/cloze probability parameter |
| Carter et al. 2019 | https://doi.org/10.1016/j.neuroimage.2019.01.018 | 41 | 2 | Surprisal of word in context |
| Huang et al. 2012 | https://doi.org/10.1016/j.brainres.2011.11.060 | 23 | 1 | Unexpected > expected word |
| Zhu et al. 2013 | https://doi.org/10.1016/j.neuroimage.2013.02.060 | 26 | 2 | Violation > low cloze > high cloze probability |
| Moberget et al. 2014 | https://doi.org/10.1523/JNEUROSCI.2264-13.2014 | 32 | 1 | Incongruent > congruent sentences |
|  |  | 32 | 3 | Sentences > scrambled sentences |
| Bedny et al. 2008 | https://doi.org/10.1093/cercor/bhn018 | 20 | 1 | Ambiguous > unambiguous |
| Gennari et al. 2007 | https://doi.org/10.1016/j.neuroimage.2007.01.015 | 17 | 1 | Ambiguous > unambiguous |
| Hirshorn & Thompson Schill 2006 | https://doi.org/10.1016/j.neuropsychologia.2006.03.035 | 10 | 5 | Switch > cluster |
| Persson et al. 2004 | https://doi.org/10.1016/j.neuroimage.2004.08.004 | 22 | 1 | High > low selection demands |
| Race et al. 2009 | https://doi.org/10.1162/jocn.2009.21132 | 26 | 3 | Different > same attribute; novel decision > repeated |
| Snyder et al. 2007 | https://doi.org/10.1162/jocn.2007.19.5.761 | 14 | 2 | Related by specific attribute > global relation |
| Zempleni et al. 2007 | https://doi.org/10.1016/j.neuroimage.2006.09.048 | 16 | 2 | Ambiguous > unambiguous |

**Supplementary Table 5:** Experiments included in semantics dataset

| **SEMANTICS** | | | | |
| --- | --- | --- | --- | --- |
| **Author & Year** | **DOI** | **N** | **Foci** | **Task contrast(s)** |
| Booth et al. 2006 | https://doi.org/10.1016/j.brainres.2005.11.097 | 13 | 1 | Semantic decision > phonological/rhyme decision |
| Demonet et al. 1992 | https://doi.org/10.1093/brain/115.6.1753 | 9 | 4 | Words > phonemes, monitoring task (press for target) |
| Devlin et al. 2003 | https://doi.org/10.1162/089892903321107837 | 12 | 1 | Semantic (man-made vs natural) > phonological (syllable number) decision |
| Fletcher et al. 1995 | https://doi.org/10.1016/0010-0277(95)00692-R | 6 | 2 | Reading physical stories > unlinked sentences |
| Gesierich et al. 2012 | https://doi.org/10.1093/cercor/bhr286 | 21 | 5 | Familiar faces (same profession judgement) > unfamiliar/scrambled faces (same-different task) |
| Gitelman et al. 2005 | https://doi.org/10.1016/j.neuroimage.2005.03.014 | 14 | 2 | Match/non-match task; semantic category > homonym/acronym/control |
| Gourovitch et al. 2000 | https://doi.org/10.1037//0894-4105.14.3.353 | 18 | 1 | Semantic (category) > phonological (letter) fluency task |
| Homae et al. 2003 | https://doi.org/10.1016/S1053-8119(03)00272-6 | 10 | 3 | Auditory sentence > jumbled phrases; judge whether probe is present |
| Kotz et al. 2002 | https://doi.org/10.1006/nimg.2002.1316 | 13 | 4 | Words > pseudoword priming; judge whether target is German |
| Kuperberg et al. 2000 | https://doi.org/10.1162/089892900562138 | 4 | 2 | Sentences with semantic violation > word strings |
| Mechelli et al. 2007 | https://doi.org/10.1002/hbm.20272 | 20 | 2 | Semantic relation judgement (category) > phonological relation judgement (share first phoneme) |
| Mummery et al. 1998 | https://doi.org/10.1162/089892998563059 | 10 | 2 | Semantic (colour/location of origin similarity) > phonological (syllable number) decision on word triads |
| Price et al. 1997 | https://doi.org/10.1162/jocn.1997.9.6.727 | 6 | 1 | Semantic (living/non-living) > phonological (syllable number) decision |
| Roskies et al. 2001 | https://doi.org/10.1162/08989290152541485 | 20 | 1 | Semantic (synonym) > phonological (rhyme) decision |
| Seghier et al. 2010 | https://doi.org/10.1523/JNEUROSCI.3377-10.2010 | 94 | 3 | Semantics > perceptual matching on stimuli triads |
| Sugiura et al. 2006 | https://doi.org/10.1016/j.neuroimage.2006.01.002 | 24 | 2 | Detect personally familiar > famous > unfamiliar names |
| Wirth et al. 2011 | <https://doi.org/10.1016/j.neuroimage.2010.10.039> | 21 | 1 | Semantic (living/non-living) > phonological (syllable number) decision on concrete words |
| Von Kriegstein et al. 2003 | https://doi.org/10.1016/S0926-6410(03)00079-X | 14 | 1 | Recognise verbal content of sentence > speech envelope noise |
| Xiao et al. 2005 | https://doi.org/10.1002/hbm.20105 | 14 | 2 | Real > pseudowords; judge whether word is real |
| Craik et al. 1999 | https://doi.org/10.1111%2F1467-9280.00102 | 8 | 2 | Judging social desirability/accuracy of trait > syllable number of trait name |
| Baumgaertner et al. 2007 | https://doi.org/10.1111/j.1460-9568.2007.05346.x | 19 | 2 | Sentences > reversed sentences; videos > scrambled videos |
| Davis et al. 2004 | https://doi.org/10.1016/S0093-934X(03)00471-1 | 11 | 1 | Words (1-back relatedness task) > letter strings |
| Devlin et al. 2002 | https://doi.org/10.1016/S0028-3932(01)00066-5 | 12 | 1 | Experiment 1: lexical decision task > letter detection |
|  |  | 8 | 6 | Experiment 2: semantic categorisation > letter categorisation task |
| Baumgaertner et al. 2002 | https://doi.org/10.1006/nimg.2002.1134 | 9 | 3 | Read sentences ending in words > pseudowords |
| Hagoort et al. 1999 | https://doi.org/10.1162/089892999563490 | 10 | 4 | Reading (overt and covert) words > pseudowords |
| Binder et al. 1999 | https://doi.org/10.1162/089892999563265 | 30 | 1 | Judge if animal is found in US/domesticated > judge if triplet contains target consonants |
| Joubert et al. 2004 | https://doi.org/10.1016/S0093-934X(03)00403-6 | 10 | 3 | Covert reading of low frequency words > pseudowords |
| Kuchinke et al. 2005 | https://doi.org/10.1016/j.neuroimage.2005.06.050 | 20 | 5 | Words > nonwords; lexical decision task |
| Binder et al. 2003 | https://doi.org/10.1162/089892903321593108 | 24 | 5 | Words > nonwords; lexical decision task |
| Meyer et al. 2002 | https://doi.org/10.1016/S0911-6044(03)00026-5 | 14 | 2 | Normal > pseudo-speech; judge whether there is meaning |
| Noppeney et al. 2003 | https://doi.org/10.1016/S0093-934X(02)00525-4 | 9 | 1 | Real > reversed words; semantic feature decision vs speaker gender decision |
| Orfanidou et al. 2006 | https://doi.org/10.1162/jocn.2006.18.8.1237 | 13 | 6 | Words > nonwords, lexical decision task |
| Perani et al. 1999 | https://doi.org/10.1016/S0028-3932(98)00073-6 | 11 | 1 | Objects (line drawings), same-different judgement > shape discrimination |
| Pilgrim et al. 2002 | https://doi.org/10.1006/nimg.2002.1105 | 14 | 4 | Semantic categorisation > same-different judgement on letter strings |
| Rissman et al. 2003 | https://doi.org/10.1162/089892903322598120 | 15 | 2 | Words > nonwords; lexical decision with priming |
| Robertson et al. 2000 | https://doi.org/10.1111%2F1467-9280.00251 | 8 | 2 | Sentence with definite/indefinite articles > letter strings |
| Damasio et al. 2001 | https://doi.org/10.1006/nimg.2001.0775 | 10 | 3 | Naming actions > control task (upside down or not) |
| Farias et al. 2005 |  | 10 | 1 | Purpose of action > nature of action |
|  |  | 10 | 2 | Purpose of action > nature of action |
| Tieleman et al. 2005 | https://doi.org/10.1016/j.neuroimage.2005.02.017 | 22 | 3 | Semantic categorisation (animal vs object) > visual perception (lower vs upper case letters) |
| Bright et al. 2004 | https://doi.org/10.1016/j.bandl.2004.01.010 | 38 | 1 | Semantic categorisation (pictures only) > shape/colour visual perception task |
| D’Arcy et al. 2007 | https://doi.org/10.1016/j.neures.2006.09.018 | 10 | 5 | Picture-word matching > baseline |
| Devlin et al. 2000 | https://doi.org/10.1006/nimg.2000.0595 | 16 | 1 | Semantic categorisation (same/different) > letter string categorisation |
| Foki et al. 2008 | https://doi.org/10.1016/j.neuroimage.2007.10.020 | 23 | 1 | Overt reading of sentences > non-meaningful tongue movements |
| Gerlach et al. 1999 | https://doi.org/10.1093/brain/122.11.2159 | 15 | 3 | Semantic decision (object vs non-object) > pattern discrimination task |
| Herbster et al. 1997 | https://doi.org/10.1002/(SICI)1097-0193(1997)5:2%3C84::AID-HBM2%3E3.0.CO;2-I | 10 | 1 | Overt reading regular/irregular words > say “hi” |
| Ikuta et al. 2006 | https://doi.org/10.1016/j.bandl.2005.10.006 | 34 | 1 | Covert reading SOV sentences > single words (verb/noun) |
| Kang et al. 2006 | https://doi.org/10.1016/j.neuroimage.2006.03.016 | 17 | 1 | Listening to speech > white noise |
| Thierry & Price 2006 | https://doi.org/10.1162/jocn.2006.18.6.1018 | 12 | 2 | Experiment 1, verbal auditory, words/stories > scrambled |
|  |  | 12 | 2 | Experiment 1, nonverbal auditory, meaningful environmental sounds > scrambled |
|  |  | 12 | 2 | Experiment 2, verbal visual, transcripts of words/stories > letter strings |
|  |  | 12 | 2 | Experiment 2, nonverbal visual, videos > distorted |
| Sergent et al. 1992 | https://doi.org/10.1093/brain/115.1.15 | 7 | 2 | Identity > gender judgement |
| Gorno-Tempini et al. 1998 | https://doi.org/10.1093/brain/121.11.2103 | 6 | 1 | Reading famous names > unfamiliar/control names |
| Leveroni et al. 2000 | https://doi.org/10.1523/JNEUROSCI.20-02-00878.2000 | 11 | 4 | Familiarity judgement; familiar > unfamiliar faces |
| Damasio et al. 2004 | https://doi.org/10.1016/j.cognition.2002.07.001 | 68 | 1 | Pictures > scrambled pictures |
| Elfgren et al. 2006 | https://doi.org/10.1016/j.neuroimage.2005.09.060 | 15 | 4 | Famous > unfamiliar faces |
| Sugiura et al. 2008 | https://doi.org/10.1016/j.neuroimage.2008.03.054 | 25 | 1 | Familiar > unfamiliar faces |
| Nielson et al. 2010 | https://doi.org/10.1016/j.bandc.2010.01.006 | 17 | 3 | Familiar > unfamiliar faces |
| Brambati et al. 2010 | https://doi.org/10.1016/j.neuroimage.2010.06.045 | 12 | 2 | Matching faces to semantic category > scrambled faces |
| Gesierich et al. 2012 | https://doi.org/10.1093/cercor/bhr286 | 12 | 2 | Familiar > unfamiliar faces |
| Dreyer & Pulvermueller 2018 | https://doi.org/10.1016/j.cortex.2017.10.021 | 28 | 1 | Silent reading of nouns > meaningless hashmark strings |
| Bulut et al. 2017 | https://doi.org/10.1371/journal.pone.0188526 | 20 | 2 | Sentences > word character list (Chinese characters) |
| Perrone-Bertolotti et al. 2017 | https://doi.org/10.3389/fnhum.2017.00325 | 24 | 1 | Semantic categorisation (living/non-living) > unreadable characters (Patterson font) |
| Liuzzi et al. 2017 | https://doi.org/10.1016/j.neuroimage.2017.02.032 | 18 | 2 | Feature/property judgement task on written words > responding whether they heard/saw a stimulus at all |
| Matchin et al. 2017 | https://doi.org/10.1016/j.cortex.2016.12.010 | 16 | 6 | Natural sentences > real word lists/phrase structure/Jabberwocky sentences |
| Zhuang & Devereux 2017 | https://doi.org/10.1080/23273798.2016.1241886 | 16 | 3 | Listening to phrases > isolated words |
| Redcay et al. 2016 | https://doi.org/10.1002/hbm.23251 | 24 | 3 | Observing communicative gestures > noncommunicative “self-adaptor” gestures |
|  |  | 24 | 2 | Reading real sentences > Jabberwocky sentences |
| Haberling et al. 2016 | https://doi.org/10.1016/j.cortex.2016.06.003 | 91 | 5 | Observing videos of pantomime gestures > unknown sign language |
|  |  | 91 | 2 | Judge synonyms > judge same/different letter strings |
| Wang et al. 2016 | https://doi.org/10.3389/fpsyg.2016.00947 | 16 | 2 | Meaningful characters > characters made from “nonsense strokes” (Chinese characters) |
| Bautista & Wilson 2016 | https://doi.org/10.1080/23273798.2015.1123281 | 12 | 2 | Clear auditory speech > scrambled & spectrally rotated speech |
| Higuchi et al. 2015 | https://doi.org/10.1002/brb3.413 | 28 | 2 | Viewing real words (kanji) > checkerboard pattern for visual baseline |
| Rogalsky et al. 2015 | https://doi.org/10.1080/23273798.2015.1066831 | 15 | 2 | Sentences > scrambled strings of words |
| Bonhage et al. 2015 | https://doi.org/10.1016/j.cortex.2015.04.011 | 18 | 5 | Meaningful declarative sentences > Jabberwocky sentences |
| AbdulSabur et al. 2014 | https://doi.org/10.1016/j.cortex.2014.01.017 | 18 | 5 | Narrative stories > nursery rhymes |
| Kyong et al. 2014 | https://doi.org/10.1162/jocn_a_00583 | 19 | 7 | Sentences with high > low intelligibility |
| Slioussar et al. 2014 | https://doi.org/10.1016/j.bandl.2014.01.006 | 21 | 4 | Viewing real > nonwords |
| Bruffaerts et al. 2013 | https://doi.org/10.1523/JNEUROSCI.1548-13.2013 | 19 | 1 | Property verification task (animal pictures/names) > scrambled images/words of animals |
| Ludersdorfer et al. 2013 | https://doi.org/10.3389/fnhum.2013.00491 | 29 | 4 | Visual: words > pseudowords & false fonts |
|  |  | 29 | 2 | Auditory: words > pseudowords & reversed speech |
| Simard et al. 2013 | https://doi.org/10.1016/j.bandl.2011.08.002 | 14 | 3 | Rule switching task; semantic > phonological rule |
| Straube et al. 2012 | https://doi.org/10.1371/journal.pone.0051207 | 16 | 2 | Verbal auditory: listening to known > unknown language |
|  |  | 16 | 2 | Nonverbal visual: viewing iconic > meaningless gesture |
| Wende et al. 2012 | https://doi.org/10.1016/j.neuroimage.2012.06.003 | 18 | 2 | Semantic/free association fluency task > phonological fluency task (rhyme) |
| Zekveld et al. 2012 | https://doi.org/10.1016/j.bandl.2012.05.006 | 18 | 2 | With > without semantically related cues, while listening to degraded/near-unintelligible speech |
| Visser et al. 2012 | https://doi.org/10.1162/jocn_a_00244 | 15 | 1 | Semantic association (CCT/PPT) > scrambled visual matching task |
|  |  | 15 | 1 | Pictures only, CCT/PPT > scrambled pictures |
|  |  | 15 | 1 | Words only, CCT/PPT > scrambled words |
| Abraham et al. 2012 | https://doi.org/10.1016/j.neuropsychologia.2012.04.015 | 19 | 2 | Semantic tasks (alternate uses, object-location) > n-back (2 levels of difficulty) |
| Zhang et al. 2012 | https://doi.org/10.1016/j.ijpsycho.2012.02.013 | 14 | 1 | Word > nonword, lexical decision task |
| Szlachta et al. 2012 | https://doi.org/10.1016/j.bandl.2012.02.007 | 21 | 11 | Words (verbs & nouns) > musical rain (passive listening) |
| Carota et al. 2012 | https://doi.org/10.1162/jocn_a_00219 | 18 | 5 | Words (tools, animals, food) > hashmark strings |
| Pulvermüller et al. 2012 | https://doi.org/10.1016/j.neuroimage.2011.12.020 | 23 | 6 | Noun-verb phrases > hashmark strings |
| Geranmayeh et al. 2012 | https://doi.org/10.1016/j.bandl.2012.02.005 | 19 | 2 | Spoken language production > meaningless tongue movements |
| Welcome & Joanisse 2012 | https://doi.org/10.1016/j.bandl.2011.12.011 | 20 | 1 | Same/different category judgement > rhyme judgement & font case judgement |
| Hervais-Adelman et al. 2012 | https://doi.org/10.1080/01690965.2012.662280 | 15 | 5 | Comprehensible > incomprehensible noise vocoded speech (target detection task) |
| Hauk & Pulvermüller 2011 | https://doi.org/10.3389/fnhum.2011.00149 | 21 | 5 | Words > hashmark strings (passive reading) |
| Birn et al. 2010 | https://doi.org/10.1016/j.neuroimage.2009.07.036 | 14 | 2 | Semantic fluency task > overlearned responses (e.g. listening months of the year) |
| Seghier et al. 2011 | https://doi.org/10.1093/cercor/bhq203 | 60 | 5 | Semantic matching (pyramids and palm trees task) > perceptual matching of meaningless stimuli |
| Obleser et al. 2011 | https://doi.org/10.1016/j.neuroimage.2011.03.035 | 14 | 1 | Less > more vocoded speech |
| Hocking et al. 2011 | https://doi.org/10.1002/hbm.21040 | 13 | 1 | Environmental sounds (yes/no semantic judgements about object properties/category) > perceptual judgements (loud/quiet) |
| Rapp & Lipka 2011 | https://doi.org/10.1162/jocn.2010.21507 | 10 | 2 | Reading words > viewing consonant strings & checkerboard patterns |
| Rodd et al. 2010 | https://doi.org/10.1016/j.neuropsychologia.2009.12.035 | 14 | 4 | Semantic judgements on sentences (auditory) > signal correlated noise |
| Khader et al. 2010 | https://doi.org/10.1016/j.brainres.2009.12.082 | 16 | 3 | Generate completing word (noun/verb) > generate rhyme/letter search |
| Kuchinke et al. 2009 | https://doi.org/10.1016/j.bandc.2008.07.014 | 15 | 1 | Semantic relatedness judgement > grammatical judgement (noun vs verb) |
| Cao et al. 2009 | https://doi.org/10.1002/hbm.20546 | 13 | 1 | Meaning judgement task > visual perception task (judging series of straight lines) |
| Leff et al. 2008 | https://doi.org/10.1523/JNEUROSCI.2903-08.2008 | 26 | 1 | Normal > reversed speech (make decision about gender of speaker) |
| Alain et al. 2008 | https://doi.org/10.1162/jocn.2008.20014 | 16 | 3 | Semantic category judgement > location of sound judgement |
| Sabri et al. 2008 | https://doi.org/10.1016/j.neuroimage.2007.09.052 | 28 | 1 | Normal speech > rotated speech; words > pseudowords |
| Hakonen et al. 2017 | https://doi.org/10.1002/brb3.789 | 20 | 2 | Less > more degraded speech |
| Boulenger et al. 2009 | https://doi.org/10.1093/cercor/bhn217 | 18 | 7 | Normal sentences > hashmark strings, passive reading |
| Lin et al. 2015 | https://doi.org/10.1162/jocn_a_00852 | 20 | 2 | Words > pseudowords (Chinese characters) |
| Ludersdorfer et al. 2016 | https://doi.org/10.1016/j.neuroimage.2015.09.039 | 29 | 6 | Semantic judgement (living/non-living) on auditory words > tone pitch judgement |
| Segal & Petrides 2012 | https://doi.org/10.1111/j.1460-9568.2011.07937.x | 9 | 1 | Writing object names > copying words |
| Carota et al. 2017 | https://doi.org/10.1093/cercor/bhw379 | 23 | 1 | Reading words > viewing hashmark strings |
| Schuil et al. 2013 | https://doi.org/10.3389/fnhum.2013.00100 | 20 | 1 | Real sentences > unpronounceable nonword sentences |
| Garbin et al. 2012 | https://doi.org/10.1371/journal.pone.0045091 | 12 | 6 | Words > pseudowords; lexical decision task |
| Vignali 2019 | https://doi.org/10.1016/j.neuroimage.2018.08.061 | 21 | 1 | Words > pseudowords (WW > PP) |
| Wu et al. 2013 | https://doi.org/10.1038/srep02049 | 19 | 5 | Words > checkerboard pattern (passive reading/viewing) |
| Groussard et al. 2010 | https://doi.org/10.1016/j.neuroimage.2009.10.039 | 11 | 1 | Verbal semantic task (matching halves of proverb) > perceptual condition (matching syllable sequences) |
| Kim et al. 2009 | https://doi.org/10.1016/j.jneuroling.2008.07.005 | 36 | 2 | Sentences > word lists; judge “semantic plausibility” |
| Bozic & Marslen-Wilson 2013 | 10.2298/PSI1304439B | 13 | 3 | Real words > musical rain (gap detection non-lexical task) |
| Bagga et al. 2013 | https://doi.org/10.1007/s12038-013-9387-7 | 18 | 1 | Semantic judgement (concrete vs abstract) > case judgement |
| Chan et al. 2009 | https://doi.org/10.1016/j.neuroimage.2009.06.078 | 22 | 2 | Synonym judgement (Chinese characters) > physical comparison of pseudocharacters |
| Malins et al. 2016 | https://doi.org/10.1016/j.neuropsychologia.2016.08.027 | 18 | 2 | Unrelated words > false font/pseudoword conditions |
| Harrington et al. 2009 | https://doi.org/10.1016/j.cortex.2007.10.015 | 8 | 2 | Drawing familiar objects > non-objects |
| Husain et al. 2012 | https://doi.org/10.1016/j.brainres.2012.08.029 | 16 | 5 | Meaningful iconic > meaningless gestures (viewing) |
| Lindenberg et al. 2012 | https://doi.org/10.1002/hbm.21258 | 20 | 1 | Meaningful iconic > meaningless gestures (viewing) |
| Marques et al. 2009 | https://doi.org/10.1016/j.cortex.2008.07.004 | 21 | 3 | Short sentences (true/false judgement) > baseline (string of cross symbols) |
| Christensen et al. 2008 | https://doi.org/10.1097%2FWNR.0b013e3283060a9d | 14 | 1 | Auditory speech (semantic judgements) > reversed speech |
| Saur et al. 2008 | https://doi.org/10.1073/pnas.0805234105 | 33 | 3 | Meaningful sentences > meaningless pseudosentences |
| Emmorey et al. 2013 | https://doi.org/10.1016/j.bandl.2013.05.001 | 14 | 2 | Semantic judgement (concrete/not) > font judgement (underlined/not) |
| Matchin et al. 2019 | https://doi.org/10.1016/j.neuropsychologia.2019.01.019 | 20 | 6 | Synonymy judgement on noun/verb phrases > lists of words |
| Zou et al. 2016 | https://doi.org/10.3389/fnhum.2015.00714 | 17 | 11 | Morpheme judgements on spoken phrases (Chinese) > tone judgement |
| Emmorey et al. 2010 | https://doi.org/10.1016/j.neuroimage.2009.08.001 | 14 | 2 | Viewing meaningful pantomime gestures > unknown ASL signs |
| Smith et al. 2012 | https://doi.org/10.1080/02643294.2012.706218 | 14 | 1 | Property verification task on word pairs > letter verification task |
| Roxbury et al. 2014 | https://doi.org/10.1186/1744-9081-10-34 | 17 | 2 | Words > pseudowords; lexical decision task |
| Hayashi et al. 2014 | https://doi.org/10.1016/j.neures.2013.10.007 | 16 | 2 | Reading words (kanji) > viewing strings of asterisks |
| Sachs et al. 2008 | https://doi.org/10.1016/j.neuropsychologia.2007.08.015 | 14 | 3 | Semantic categorisation task (word triads) > letter categorisation task |
| Erb et al. 2013 | https://doi.org/10.1523/JNEUROSCI.4596-12.2013 | 30 | 6 | High > low intelligibility; repeat sentence back |
| Oblseser et al. 2008 | https://doi.org/10.1523/JNEUROSCI.1290-08.2008 | 16 | 3 | High > low intelligibility, vocoded speech |
| Just et al. 2008 | https://doi.org/10.1016/j.brainres.2007.12.075 | 29 | 1 | Listening to speech during simulated driving task > simulated driving task alone |
| Kinno et al. 2008 | https://doi.org/10.1002/hbm.20441 | 14 | 5 | Sentences > pictures/letter strings |
| Jensen et al. 2011 | https://doi.org/10.1016/j.eplepsyres.2010.12.003 | 12 | 4 | Words > nonwords, lexical decision task |
| Barros-Loscertales et al. 2012 | https://doi.org/10.1093/cercor/bhr324 | 59 | 3 | Words > hashmark strings, passive viewing |
| Europa et al. 2019 | https://doi.org/10.3389/fnhum.2019.00027 | 21 | 1 | Listening to sentences (sentence-picture verification task) > reversed speech |
| Raettig & Kotz 2008 | https://doi.org/10.1016/j.neuroimage.2007.09.030 | 16 | 1 | Words > pseudowords, lexical decision task |
| Chow et al. 2008 | https://doi.org/10.1016/j.neuroimage.2007.11.044 | 15 | 7 | Words > pseudowords; lexical decision task on items embedded within written narrative |
| Moseley et al. 2012 | https://doi.org/10.1093/cercor/bhr238 | 18 | 7 | Words > hashmark strings |
| Weiss et al. 2015 | https://doi.org/10.1016/j.neuroimage.2015.07.029 | 18 | 2 | Read words > say “pass” to asterisk stringss |
| Chang et al. 2019 | https://doi.org/10.1002/hbm.24502 | 26 | 4 | Words > maths |
| Vagharchakian et al. 2012 | https://doi.org/10.1523/JNEUROSCI.5685-11.2012 | 16 | 5 | High > low intelligibility (sentences) |
|  |  | 16 | 3 | Auditory: high > low intelligibility |
|  |  | 16 | 2 | Visual: high > low intelligibility |
| Schmitt et al. 2019 | https://doi.org/10.1080/23273798.2018.1533139 | 40 | 2 | Listening to sentences in intelligible (German) > unintelligible language (Vietnamese) |
| Chouinard et al. 2008 | https://doi.org/10.1016/j.neuroimage.2008.02.011 | 14 | 3 | Naming objects from images > counting scrambled objects |
| Zvyagintsev et al. 2013 | https://doi.org/10.1111/ejn.12140 | 15 | 1 | Visual imagery of common objects > counting |
| Bick et al. 2008 | https://doi.org/10.1162/jocn.2008.20028 | 14 | 5 | Judge whether word pairs are related > judge whether line patterns are identical |
| Raposo et al. 2016 | https://doi.org/10.1016/j.neuropsychologia.2016.06.036 | 18 | 1 | Semantic (pleasantness judgement) > perceptual task (letter number decision) |
| Peelle et al. 2010 | https://doi.org/10.1016/j.neuroimage.2010.05.015 | 6 | 2 | Unambiguous spoken sentences > signal correlated noise |
| Gutchess et al. 2010 | https://doi.org/10.1093/scan/nsp059 | 20 | 1 | Judge semantic relationship (category or association) > judge whether words are identical |
| Bhattasali et al. 2019 | https://doi.org/10.1080/23273798.2018.1518533 | 51 | 3 | Bottom-up parser action count |
| Marques et al. 2008 | https://doi.org/10.1016/j.brainres.2007.11.070 | 21 | 1 | Semantic judgement (yes/no to motion property) > strings of crosses |
| Herve et al. 2012 | https://doi.org/10.1016/j.neuroimage.2012.03.073 | 51 | 9 | Emotion judgement > grammar judgement (sentences) |
| Bonhage et al. 2014 | https://doi.org/10.1162/jocn_a_00566 | 18 | 4 | Sentence fragments > word lists random order; judge order of words |
| Holle et al. 2008 | https://doi.org/10.1016/j.neuroimage.2007.10.055 | 17 | 7 | Auditory sentence + disambiguating meaningful gesture > auditory sentence + unrelated “grooming” gesture |
| Grindrod et al. 2014 | https://doi.org/10.1016/j.bandl.2014.10.001 | 23 | 2 | Words > nonwords |
| Snijders et al. 2009 | https://doi.org/10.1093/cercor/bhn187 | 28 | 4 | Sentences > word sequences; passive viewing |
| Chiao et al. 2009 | https://doi.org/10.1016/j.neuropsychologia.2008.09.023 | 12 | 4 | Social status change > colour change detection (photographs) |
| Metz-Lutz et al. 2010 | https://doi.org/10.3389/fnhum.2010.00059 | 11 | 1 | Theatrical/narrative events > non-events (auditory & visual) |
| Rogalsky & Hickok 2009 | https://doi.org/10.1093/cercor/bhn126 | 14 | 1 | Semantic anomaly > syntactic/grammar error judgement; sentences > noun lists (localiser) |
| Sun et al. 2017 | https://doi.org/10.1016/j.jmr.2016.12.012 | 11 | 3 | Semantic relatedness judgement > font size (word triads) |
| Liu et al. 2009 | https://doi.org/10.1162/jocn.2009.21141 | 16 | 2 | Semantic relatedness judgement (word pairs) > identity judgement on tone pairs |
| Diaz & McCarthy 2009 | https://doi.org/10.1016/j.brainres.2009.05.043 | 16 | 2 | Words > nonwords; working memory task |
| Taminato et al. 2014 | https://doi.org/10.1016/j.neures.2014.09.001 | 35 | 2 | Object recognition (with varying visual noise) > overlearned object recognition (control) |
| Wright et al. 2008 | https://doi.org/10.1002/hbm.20443 | 34 | 1 | Naming task > matching (1-back) task |
|  |  | 34 | 2 | Verbal only: Naming task > matching (1-back) task |
|  |  | 34 | 2 | Nonverbal only: Naming task > matching (1-back) task |
| Leung & Alain 2010 | https://doi.org/10.1016/j.neuroimage.2010.12.055 | 16 | 1 | Category (human/musical instrument/other) > location (azimuth) judgements on sounds |
| McGettigan et al. 2012 | https://doi.org/10.1016/j.neuropsychologia.2012.01.010 | 26 | 30 | Main effect of visual & auditory clarity |
| Takeichi et al. 2010 | https://doi.org/10.1016/j.neuroimage.2009.10.063 | 23 | 3 | Normal speech > reversed & modulated speech |
| Pallier et al. 2011 | https://doi.org/10.1073/pnas.1018711108 | 40 | 4 | Normal > Jabberwocky sentences |
| Geudiche et al. 2016 | https://doi.org/10.1016/j.cortex.2016.03.014 | 16 | 10 | Intelligibility from cueing/repetition |
| Soch et al. 2017 | https://doi.org/10.1093/cercor/bhw206 | 110 | 2 | Self/other trait judgement task (self-referential processing task) > syllable counting |
| Chou et al. 2009 | https://doi.org/10.1007/s00221-009-1942-y | 31 | 2 | Semantic judgement (are Chinese character pairs related) > perceptual judgement (are pseudocharacters identical) |
| Davis et al. 2011 | https://doi.org/10.1162/jocn_a_00084 | 12 | 7 | Intelligibility (coherent & anomalous prose) |
| Egidi & Caramazza 2016 | https://doi.org/10.1162/jocn_a_00982 | 28 | 1 | Semantic judgement (consistency of story ending) > listening only |
| Clos et al. 2014 | https://doi.org/10.1002/hbm.22151 | 29 | 2 | Effect of propositional prior |
| Van Ettinger-Veenstra et al. 2016 | https://doi.org/10.3389/fnhum.2016.00110 | 27 | 2 | Sentence-reading > viewing symbol sequences |
| Graves et al. 2010 | https://doi.org/10.1016/j.neuroimage.2010.06.055 | 23 | 1 | Experiment 1: meaningful (forward) > reversed phrases |
|  |  | 22 | 1 | Experiment 2: meaningful > reversed phrases; meaningful/reversed/nonwords judgement |
| Adank et al. 2012 | https://doi.org/10.1016/j.neuropsychologia.2011.10.024 | 26 | 3 | Clear > noisy speech (high > low intelligibility) |
| Wright et al. 2011 | https://doi.org/10.1162/jocn.2010.21450 | 14 | 4 | Auditory speech > musical rain |

**Supplementary Table 6:** Experiments included in theory of mind dataset

| **THEORY OF MIND** | | | | |
| --- | --- | --- | --- | --- |
| **Author & Year** | **DOI** | **N** | **Foci** | **Task contrast(s)** |
| **Experiments included in full dataset and subset without faces** | | | | |
| Abraham et al. 2010 | https://doi.org/10.1080/17470910903166853 | 22 | 3 | Reading sentences about belief/desire > control |
| Abraham et al. 2008 | <https://doi.org/10.1016/j.concog.2008.03.011> | 17 | 4 | Reading sentences about person-related mental states > non-mental (relational/non-intentional) sentences |
| Aichhorn et al. 2006 | <https://doi.org/10.1016/j.neuroimage.2005.10.026> | 18 | 1 | Other > self perspective; perspective-taking task on visual scene |
| Aichhorn et al. 2008 | <https://doi.org/10.1162/jocn.2009.21082> | 21 | 6 | False belief > false photograph task |
| Assaf et al. 2009 | <https://doi.org/10.1007/s11682-008-9047-y> | 19 | 2 | Main effect of mentalisation (show > no-show) |
| Bahnemann et al. 2010 | <https://doi.org/10.1093/scan/nsp045> | 25 | 3 | Theory of mind > appearance judgement; viewing picture pairs/animations |
| Bodden et al. 2013 | <https://dx.doi.org/10.2478%2Fv10053-008-0129-6> | 30 | 3 | Affective theory of mind > physical control; Yoni task |
| Brune et al. 2008 | <https://doi.org/10.1016/j.neuropsychologia.2008.01.023> | 13 | 5 | Theory of mind > physical properties; cartoon stories |
| Chaminade et al. 2012 | <https://doi.org/10.3389/fnhum.2012.00103> | 19 | 1 | Intentional agent/opponent > randomised moves; computerised game |
| Cheung et al. 2012 | <https://doi.org/10.1016/j.ijpsycho.2011.12.002> | 23 | 3 | False belief > physical; Sally-Anne paradigm |
| Decety et al. 2004 | <https://doi.org/10.1016/j.neuroimage.2004.05.025> | 12 | 2 | Competitive > independent; game with tokens |
| Dodell-Feder et al. 2011 | <https://doi.org/10.1016/j.neuroimage.2010.12.040> | 62 | 3 | False belief > false photograph task |
| Dohnel et al. 2012 | <https://doi.org/10.1016/j.neuroimage.2012.01.073> | 18 | 6 | Belief reasoning > physical reasoning (Sally-Anne) |
| Dofour et al. 2013 | <https://doi.org/10.1371/journal.pone.0075468> | 462 | 7 | False belief > false photograph task |
| Elliott et al. 2006 | <https://doi.org/10.1080/17470910601041358> | 12 | 2 | Cooperation > playing alone; gambling paradigm |
| Feng et al. 2014 | <https://doi.org/10.1016/j.neulet.2014.01.059> | 17 | 3 | Point-to-other > point-to-self jokes |
| Ferstl & von Cramon 2002 | <https://doi.org/10.1006/nimg.2002.1247> | 9 | 2 | Theory of mind > control sentences |
| Fukui et al. 2006 | <https://doi.org/10.1016/j.neuroimage.2006.03.039> | 16 | 1 | Human > computer component (dilemma game) |
| Gallagher et al. 2000 | <https://doi.org/10.1016/S0028-3932(99)00053-6> | 6 | 6 | Theory of mind cartoons/stories > non-TOM |
| Gobbini et al. 2007 | <https://doi.org/10.1162/jocn.2007.19.11.1803> | 21 | 8 | False belief > physical stories; social > random/non-social motion |
| Grezes et al. 2004 | <https://doi.org/10.1016/S1053-8119(03)00665-7> | 6 | 3 | False > correct expectation (viewing people lifting weights) |
| Gweon et al. 2012 | <https://doi.org/10.1111/j.1467-8624.2012.01829.x> | 8 | 3 | Mental > physical stories |
| Harris et al. 2005 | <https://doi.org/10.1016/j.neuroimage.2005.05.021> | 12 | 4 | Attribution of behaviour to person/character > other factors |
| Herve et al. 2013 | <https://doi.org/10.1371/journal.pone.0054400> | 42 | 4 | Theory of mind (belief/deception/empathy) > plausibility judgements (sentences) |
| Hooker et al. 2008 | <https://doi.org/10.1093/scan/nsn019> | 20 | 7 | False belief > true belief |
| Jenkins et al. 2010 | <https://doi.org/10.1093/cercor/bhp109> | 15 | 2 | Mentalising > non-social stories |
| Jimura et al. 2010 | <https://doi.org/10.1016/j.brainres.2010.03.016> | 34 | 1 | Mental state reasoning > physical control reasoning |
| Kana et al. 2009 | <https://doi.org/10.1080/17470910802198510> | 12 | 4 | ToM/social > random animations of shapes |
| Kestemont et al. 2013 | <https://doi.org/10.1093/scan/nss022> | 17 | 1 | Person causal events > semantic baseline judgement |
| Kircher et al. 2009 | <https://doi.org/10.1016/j.neulet.2009.03.026> | 12 | 1 | Human player (prisoners dilemma) > low level baseline |
| Kliemann et al. 2008 | <https://doi.org/10.1016/j.neuropsychologia.2008.06.010> | 26 | 2 | False belief > false photograph |
| Koelkebeck et al. 2011 | <https://doi.org/10.1080/17470919.2011.620763> | 15 | 2 | ToM > random motion of animated shapes |
| Krach et al. 2008 | <https://doi.org/10.1371/journal.pone.0002597> | 20 | 1 | Human opponent (prisoners dilemma) > control |
| Lee et al. 2011 | <https://doi.org/10.1080/17470919.2011.620774> | 14 | 16 | False belief > simple reading; false belief > false photo |
| Lombardo et al. 2010 | <https://doi.org/10.1162/jocn.2009.21287> | 33 | 2 | Mentalising > physical judgements about others |
| Ma et al. 2011 | <https://doi.org/10.1080/17470919.2010.485884> | 15 | 2 | Diagnostic > irrelevant personality traits |
| Malhi et al. 2008 | <https://doi.org/10.1111/j.1399-5618.2008.00643.x> | 20 | 3 | ToM > random movements of animated shapes |
| Mano et al. 2009 | <https://doi.org/10.1016/j.neuropsychologia.2008.12.011> | 18 | 5 | Consistent > inconsistent protagonist in written stories |
| Marjoram et al. 2006 | <https://doi.org/10.1016/j.neuroimage.2006.02.011> | 13 | 1 | ToM-related joke > physical joke (cartoons) |
| Martin & Weisberg et al. 2003 | <https://doi.org/10.1080/02643290342000005> | 12 | 6 | Social > mechanical animations |
| Mason et al. 2008 | <https://doi.org/10.1016/j.neuropsychologia.2007.07.018> | 18 | 2 | Emotional inference > rest; intentional inference > rest |
| McAdams & Krawczyk 2011 | <https://doi.org/10.1016/j.pscychresns.2011.06.016> | 17 | 5 | People/social > visuospatial/random/bumper car animations |
| Mier et al. 2010 | <https://doi.org/10.1017/S0033291709992133> | 16 | 3 | Affective ToM judgements > emotion recognition |
| Mitchell 2008 | <https://doi.org/10.1093/cercor/bhm051> | 20 | 1 | False belief > false photograph |
| Modinos et al. 2010 | <https://doi.org/10.1016/j.neuropsychologia.2010.09.030> | 36 | 2 | Cognitive > affective mental state judgements |
| Moran et al. 2012 | <https://doi.org/10.1523/JNEUROSCI.5511-11.2012> | 31 | 6 | Social > mechanical animations; false belief > false photograph task |
| Murphy et al. 2010 | <https://doi.org/10.1016/j.schres.2009.11.009> | 10 | 1 | Social evaluation of others > semantic evaluation |
| Otsuka et al. 2009 | <https://doi.org/10.1016/j.neulet.2009.07.064> | 22 | 2 | Theory of mind judgement > tense consistency judgement |
| Otsuka et al. 2011 | <https://doi.org/10.1371/journal.pone.0019320> | 22 | 1 | Theory of mind judgement > tense consistency judgement |
| Polosan et al. 2011 | <https://doi.org/10.1016/j.bandc.2011.08.018> | 14 | 1 | Human > computer opponent (Stroop game) |
| Rabin & Rosenbaum 2012 | <https://doi.org/10.1016/j.neuroimage.2012.05.002> | 18 | 11 | Theory of mind judgements for familiar > unfamiliar others |
| Rapp et al. 2010 | <https://doi.org/10.1016/j.bandl.2009.11.007> | 15 | 1 | Reading ironic > literal sentences |
| Rilling et al. 2008 | <https://doi.org/10.1016/j.neuroimage.2008.03.044> | 20 | 1 | Human opponent (prisoners dilemma) > lone gambling |
| Rilling et al. 2004 | <https://doi.org/10.1016/j.neuroimage.2004.04.015> | 19 | 1 | Human opponent > computer/control (gambling) |
| Roser et al. 2012 | <https://doi.org/10.1016/j.schres.2012.05.020> | 14 | 5 | Theory of mind > non-theory of mind questions (cartoons) |
| Ross & Olson 2010 | <https://doi.org/10.1016/j.neuroimage.2009.11.012> | 15 | 3 | Social > mechanical/bumper car animations |
| Rothmayr et al. 2011 | <https://doi.org/10.1016/j.neuroimage.2010.12.052> | 12 | 4 | False belief > true belief (cartoons) |
| Saft et al. 2013 | <https://doi.org/10.1007/s11682-012-9209-9> | 26 | 3 | Theory of mind > physical/control questions about cartoon |
| Samson et al. 2008 | <https://doi.org/10.1080/17470910701745858> | 17 | 1 | Theory of mind joke > semantic joke (cartoon) |
| Saxe & Kanwisher 2003 | <https://doi.org/10.1016/S1053-8119(03)00230-1> | 25 | 3 | Theory of mind > mechanical inference stories |
|  |  | 21 | 2 | False belief > false photograph |
| Saxe & Powell 2006 | [https://doi.org/10.1111/j.1467-9280.2006.01768.x](https://doi.org/10.1111%2Fj.1467-9280.2006.01768.x) | 12 | 4 | False belief > false photo; thoughts > bodily sensations/appearance |
| Schnell et al. 2011 | <https://doi.org/10.1016/j.neuroimage.2010.08.024> | 21 | 5 | Affective ToM > visuospatial judgements |
| Seger et al. 2004 | <https://doi.org/10.1016/j.neuropsychologia.2004.02.003> | 12 | 1 | Judgements about others’ preference > vowel number |
| Shibata et al. 2010 | <https://doi.org/10.1016/j.brainres.2009.10.030> | 13 | 1 | Ironic > literal sentences |
| Sommer et al. 2007 | <https://doi.org/10.1016/j.neuroimage.2007.01.042> | 16 | 1 | False belief > true belief cartoons |
| Sommer et al. 2010 | <https://doi.org/10.1016/j.pscychresns.2010.01.007> | 14 | 1 | Goal-related emotion > reality judgements |
| Spiers & Maguire 2006 | <https://doi.org/10.1016/j.neuropsychologia.2006.03.028> | 20 | 3 | Thinking about minds; ToM thoughts > no directed thought |
| Spotorno et al. 2012 | <https://doi.org/10.1016/j.neuroimage.2012.06.046> | 20 | 2 | Ironic > literal sentences |
| Sripada et al. 2009 | <https://dx.doi.org/10.1097%2FWNR.0b013e32832d0a67> | 26 | 1 | Human > computer opponent, gambling task |
| Uchiyama et al. 2006 | <https://doi.org/10.1016/j.brainres.2006.09.088> | 20 | 1 | Sarcastic/non-sarcastic > unrelated sentences |
| Van der Meer et al. 2011 | <https://doi.org/10.1016/j.neuroimage.2011.03.053> | 19 | 2 | High > low information inhibition (Sally-Anne scenario) |
| Van Hoeck et al. 2014 | <https://doi.org/10.1016/j.neuroimage.2013.12.043> | 19 | 1 | False belief > reality reasoning |
| Vanderwal et al. 2008 | <https://doi.org/10.1016/j.neuroimage.2008.03.058> | 11 | 5 | Social > mechanical/bumper car animations |
| Varga et al. 2013 | <https://doi.org/10.1016/j.bandl.2013.05.017> | 24 | 4 | Irony > control/physical (auditory speech) |
| Veroude et al. 2012 | <https://doi.org/10.1080/13803395.2011.630650> | 24 | 1 | Self/other emotion or action > baseline/fixation (reading) |
| Vollm et al. 2006 | <https://doi.org/10.1016/j.neuroimage.2005.07.022> | 13 | 2 | Empathy/ToM > physical cartoons |
| Walter et al. 2004 | <https://doi.org/10.1162/0898929042947838> | 13 | 6 | Communicative intention/private intention > physical causality cartoons |
|  |  | 12 | 11 | Social interaction/communicative intention > physical causality cartoons |
| Walter et al. 2009 | <https://doi.org/10.1093/scan/nsn047> | 12 | 11 | Social interaction/communicative intention > physical causality cartoons |
| Wang et al. 2006 | <https://doi.org/10.1093/scan/nsl018> | 12 | 2 | Ironic > non-ironic stories |
| Young et al. 2007 | <https://doi.org/10.1073/pnas.0701408104> | 10 | 2 | False belief > false photograph |
|  |  | 17 | 2 | False belief > false photograph |
| Young & Saxe 2010 | <https://doi.org/10.1016/j.neuropsychologia.2010.05.012> | 17 | 4 | False belief > false photograph; mental states > physical events |
| Young & Saxe 2008 | <https://doi.org/10.1016/j.neuroimage.2008.01.057> | 17 | 2 | False belief > false photograph |
| Young & Saxe 2009 | <https://doi.org/10.1162/jocn.2009.21137> | 14 | 3 | False belief > false photograph |
| Young et al. 2011 | <https://doi.org/10.1080/17470919.2010.529712> | 17 | 2 | False belief > false photograph |
| Zaitchik et al. 2010 | <https://doi.org/10.1016/j.neuropsychologia.2010.04.031> | 15 | 2 | Belief > control/physical sentences |
| Kobayashi et al. 2008 | https://doi.org/10.1093/scan/nsm039 | 16 | 1 | Theory of mind > physical stories |
| Alderson-Day et al. 2016 | https://doi.org/10.1093/scan/nsv094 | 19 | 2 | Theory of mind > physical stories |
| Bartholomeusz et al. 2018 | https://doi.org/10.1016/j.pscychresns.2018.08.011 | 22 | 2 | Theory of mind > physical stories |
| Chakroff et al. 2016 | https://doi.org/10.1093/scan/nsv131 | 23 | 2 | False belief > false photograph localiser |
| Cole et al. 2019 | https://doi.org/10.1016/j.neuropsychologia.2018.11.008 | 20 | 2 | Infer intention > judge success of an action (video clips) |
| Corradi-Dell’Acqua et al. 2014 | https://doi.org/10.1093/scan/nst097 | 46 | 4 | Belief/emotion judgements > physical photo judgements |
| Deuse et al. 2016 | https://doi.org/10.1093/scan/nsw094 | 38 | 3 | Valence judgement of social scenes > location judgements |
| Dodell-Feder et al. 2014 | https://doi.org/10.1016/j.nicl.2013.11.006 | 18 | 5 | Thoughts > appearance (judging truth of statements) |
| Geiger et al. 2019 | https://doi.org/10.1016/j.neuroimage.2019.116102 | 32 | 1 | Mood > movement identification (animated mannequin) |
| Hartwright et al. 2015 | https://doi.org/10.1016/j.neuropsychologia.2014.12.015 | 21 | 4 | False belief > false photograph |
| Hennion et al. 2016 | https://doi.org/10.1016/j.neuropsychologia.2016.11.007 | 25 | 3 | Theory of mind > goal directed & random movements (animated shapes) |
| Jack & Pelphrey 2015 | https://doi.org/10.1093/cercor/bhu146 | 34 | 3 | Theory of mind > random movements (animated shapes) |
| Jacoby et al. 2016 | https://doi.org/10.1016/j.neuroimage.2015.11.025 | 17 | 7 | False belief > false photo; theory of mind > pain events (passive movie watching) |
| Jenkins et al. 2014 | https://doi.org/10.1371/journal.pone.0105341 | 19 | 5 | False belief > false photo; individual/group attributions > non-theory of mind baseline |
| Kandylaki et al. 2015 | https://doi.org/10.1002/hbm.22907 | 20 | 3 | False belief > physical causality stories |
| Lavoie et al. 2016 | https://doi.org/10.1016/j.cortex.2016.04.017 | 19 | 5 | Intention > physical inference (multiple time points) |
| Lee & McCarthy 2016 | https://doi.org/10.1093/cercor/bhu292 | 19 | 2 | False belief > false photograph |
| Lewis et al. 2017 | https://doi.org/10.1093/scan/nsx034 | 17 | 2 | False belief > factual memory judgements |
| Lin et al. 2018 | https://doi.org/10.3758/s13415-018-0568-6 | 39 | 1 | False belief > false photograph |
| Moessnang et al. 2016 | https://doi.org/10.1093/scan/nsw098 | 46 | 3 | Theory of mind > goal directed movement |
| Mohnke et al. 2016 | https://doi.org/10.1093/scan/nsv111 | 297 | 2 | Detecting changes in mental state > detecting change in number of people present |
| Naughtin et al. 2017 | https://doi.org/10.1002/hbm.23700 | 22 | 4 | False belief > false photograph |
| Oliver et al. 2018 | https://doi.org/10.1093/scan/nsy013 | 35 | 1 | False belief > false photograph |
| Otti et al. 2015 | https://doi.org/10.1371/journal.pone.0135912 | 20 | 2 | Theory of mind > random movements (animated shapes) |
| Powell et al. 2017 | https://doi.org/10.1016/j.neuroscience.2017.04.042 | 12 | 1 | Theory of mind > physical judgements, story endings |
| Schmitgen et al. 2016 | https://doi.org/10.1016/j.neuroimage.2016.01.029 | 21 | 5 | Detecting changes in mental state > detecting change in number of people present |
| Schneider et al. 2014 | https://doi.org/10.1016/j.neuroimage.2014.07.014 | 16 | 4 | False belief > false photograph |
| Specht & Wigglesworth 2018 | https://doi.org/10.1111/sjop.12410 | 18 | 2 | Theory of mind > cartoon matching (story ending judgement) |
| Wang et al. 2015 | https://doi.org/10.3389/fnbeh.2015.00133 | 56 | 1 | Theory of mind > physical causality, story ending judgement task |
| Willert et al. 2015 | https://doi.org/10.1111/bdi.12352 | 81 | 3 | Detecting changes in mental state > detecting change in number of people present |
| Cassidy et al. 2021 | https://doi.org/10.1080/13825585.2020.1718060 | 40 | 2 | False belief > false photograph |
| Kirkovski et al. 2016 | https://doi.org/10.1007/s10803-015-2639-7 | 23 | 4 | Theory of mind > goal directed/random movement, animated shapes task |
| Schlaffke et al. 2014 | https://doi.org/10.1002/hbm.22610 | 39 | 7 | Inferring mental states > physical judgements |
| **Experiments included in full dataset but not subset without faces** | | | | |
| Adams et al. 2010 | <https://doi.org/10.1162/jocn.2009.21187> | 28 | 7 | Mental state > gender judgement (photos) |
| Ampe et al. 2014 | <https://doi.org/10.1080/13554794.2012.741251> | 20 | 3 | Unusual intention/means > normal (photos) |
| Baron-Cohen et al. 1999 | <https://doi.org/10.1017/S0021963001006643> | 12 | 7 | Mental > gender judgement (RMET) |
| Canessa et al. 2012 | <https://doi.org/10.1371/journal.pone.0042347> | 27 | 7 | Cooperative/social interaction > landscapes (photos) |
| Castelli et al. 2010 | <https://doi.org/10.1016/j.neuropsychologia.2010.05.005> | 12 | 1 | Mental > gender judgement (RMET) |
| Contreras et al. 2013 | <https://doi.org/10.1162/jocn_a_00403> | 25 | 4 | Mental > physical judgements (individuals & groups) |
|  |  | 14 | 4 | Mental > physical judgements; false belief > false photo |
| De Achaval et al. 2012 | <https://doi.org/10.1016/j.schres.2011.11.010> | 14 | 5 | Mental > gender judgement (RMET) |
| Derntl et al. 2010 | <https://doi.org/10.1016/j.psyneuen.2009.10.006> | 24 | 2 | Perspective taking (photographs of faces) |
| Focquaert et al. 2010 | <https://doi.org/10.1016/j.brainresbull.2010.08.008> | 12 | 4 | Mental > gender judgement (RMET) |
| Gallagher & Frith 2004 | <https://doi.org/10.1016/j.neuropsychologia.2004.05.006> | 12 | 6 | Expressive > instrumental gestures; instrumental/expressive gestures > null (videos) |
| German et al. 2004 | <https://doi.org/10.1162/0898929042947892> | 16 | 4 | Videos of pretend > real actions |
| Hooker et al. 2010 | <https://doi.org/10.1016/j.brainres.2009.10.006> | 15 | 5 | Social change > no change |
| Krach et al. 2009 | <https://doi.org/10.1186/1471-2202-10-9> | 12 | 3 | Human opponent > computer/low level baseline |
| Liew et al. 2011 | <https://doi.org/10.1002/hbm.21164> | 18 | 4 | Gesture videos > still photos |
| Rabin et al. 2010 | <https://doi.org/10.1162/jocn.2009.21344> | 18 | 2 | Vivid > vague theory of mind; theory of mind > autobiographical photos |
| Reniers et al. 2014 | <https://doi.org/10.1080/17470919.2013.861360> | 15 | 1 | Theory of mind for sad > neutral pictures |
| Russell et al. 2010 | <https://doi.org/10.1176/appi.ajp.157.12.2040> | 7 | 2 | Mental > gender judgement (RMET) |
| Schiffer et al. 2013 | <https://doi.org/10.1371/journal.pone.0060278> | 22 | 2 | Mental > gender judgement (RMET) |
| Spunt & Lieberman 2012a | <https://doi.org/10.1523/JNEUROSCI.5715-11.2012> | 21 | 2 | Answer questions about motivation > implementation |
| Spunt & Lieberman 2012b | <https://doi.org/10.1016/j.neuroimage.2011.10.005> | 22 | 4 | Cause > mechanics of facial expressions |
| Spunt et al. 2011 | <https://doi.org/10.1162/jocn.2010.21446> | 15 | 2 | Why > what/how of actions (videos) |
| Todorov et al. 2007 | <https://doi.org/10.1016/j.neuropsychologia.2006.04.018> | 10 | 2 | Familiar > novel faces (trait association) |
| Wolf et al. 2010 | <https://doi.org/10.1016/j.neuroimage.2009.08.060> | 18 | 3 | Theory of mind > physical inference (videos) |
| Kanske et al. 2015 | https://doi.org/10.1016/j.neuroimage.2015.07.082 | 178 | 6 | Theory of mind > factual reasoning (EmpaToM) |
|  |  | 25 | 15 | False belief > false photo; theory of mind > factual reasoning (EmpaToM) |
| Libero et al. 2014 | https://doi.org/10.1186/2040-2392-5-50 | 22 | 1 | Judgement of intention > means (handling of objects) |
| Spunt & Adolphs 2014 | https://doi.org/10.1016/j.neuroimage.2014.05.023 | 29 | 2 | Why > how inferences |
|  |  | 21 | 2 | Why > how inferences |
| Tholen et al. 2020 | https://doi.org/10.1002/hbm.24966 | 130 | 5 | Theory of mind/mental state attribution > factual reasoning (EmpaToM) |
| Thye et al. 2018 | https://doi.org/10.1016/j.neuroscience.2018.01.045 | 18 | 4 | Emotion > gender (RMET, reading mind in the voice); intentional > physical causality judgements |

**Supplementary Table 7:** Experiments included in tools dataset

| **TOOLS** | | | | |
| --- | --- | --- | --- | --- |
| **Author & Year** | **DOI** | **N** | **Foci** | **Task contrast(s)** |
| Anzellotti et al. 2011 | <https://doi.org/10.1162/jocn.2010.21567> | 11 | 4 | Tools > animals, categorisation of greyscale images |
| Boronat et al. 2005 | <https://doi.org/10.1016/j.cogbrainres.2004.11.001> | 15 | 1 | Tools > scrambled, line drawings & names |
| Bohlhalter et al. 2009 | <https://doi.org/10.1093/cercor/bhn168> | 15 | 7 | Planning tool use movements > rest |
| Buxbaum et al. 2006 | <https://doi.org/10.1016/j.brainres.2006.08.010> | 15 | 1 | Object use action decision > scrambled baseline (photos) |
| Chao et al. 1999 | <https://doi.org/10.1038/13217> | 8 | 1 | Tools > animals, viewing greyscale images |
|  |  | 14 | 2 | Tools > animals, covert naming of greyscale images |
| Chao et al. 2002 | <https://doi.org/10.1093/cercor/12.5.545> | 7 | 1 | Tools > animals, covert naming of greyscale images |
| Creem-Regehr & Lee 2005 | <https://doi.org/10.1016/j.cogbrainres.2004.10.006> | 12 | 13 | Tools > scrambled images, viewing greyscale images |
| Choi et al. 2001 | <https://doi.org/10.1007/s002210100777> | 10 | 1 | Performing tool actions > finger tapping |
| Canessa et al. 2008 | <https://doi.org/10.1093/cercor/bhm110> | 15 | 1 | Manipulation/action matching of tool pairs > context similarity of pairs (photos) |
| Damasio et al. 1996 | <https://doi.org/10.1038/380499a0> | 9 | 3 | Tools > unfamiliar faces, overt naming from photos |
| Fridman et al. 2006 | <https://doi.org/10.1016/j.neuroimage.2005.07.026> | 19 | 1 | Planning tool use > rest |
| Grezes & Decety 2002 | <https://doi.org/10.1016/S0028-3932(01)00089-6> | 10 | 5 | Tools > non-object size judgement |
| Grafton et al. 1996 | <https://doi.org/10.1007/BF00227183> | 7 | 2 | Imagined grasping > observing grasping of real objects |
| Handy et al. 2003 | <https://doi.org/10.1038/nn1031> | 14 | 3 | Viewing line drawings of tools > non-tools/rest |
| Kiyosawa et al. 1996 | <https://doi.org/10.1007/BF00695250> | 4 | 4 | Naming tools > rest |
| Kellenbach et al. 2003 | <https://doi.org/10.1162/089892903321107800> | 9 | 2 | Tools > scrambled objects; answer yes/no question about associated actions |
| Liljestrom et al. 2008 | <https://doi.org/10.1016/j.neuroimage.2008.03.016> | 15 | 1 | Covert naming of tools from line drawings > rest |
| Moscovitch et al. 1995 | <https://doi.org/10.1073/pnas.92.9.3721> | 13 | 3 | Object memory > perceptual (similarity judgement) baseline |
| Okada et al. 2000 | <https://doi.org/10.1016/S0304-3940(00)01612-8> | 12 | 1 | Tools > animals; covert naming from greyscale images |
| Phillips et al. 2002 | <https://doi.org/10.1080/13506280143000610> | 26 | 3 | Judging action (twist vs no twist) > screen size judgement |
| Peran et al. 2010 | <https://doi.org/10.1016/j.brainres.2010.02.082> | 12 | 1 | Imagined use (from line drawings) > fixation |
| Tranel et al. 2005 | <https://doi.org/10.1016/j.bandl.2004.01.011> | 10 | 2 | Tools > faces; covert naming from photographs |
| Valyear et al. 2007 | <https://doi.org/10.1016/j.neuroimage.2007.03.031> | 11 | 9 | Tools > animals; covert naming from photos |
| Vingerhoets et al. 2009 | <https://doi.org/10.1016/j.neuroimage.2009.05.100> | 15 | 2 | Object-based actions > pointing (imagined movement) |
| Vingerhoets 2008 | <https://doi.org/10.1016/j.neuroimage.2007.12.058> | 14 | 2 | Familiar > unfamiliar tools; orientation judgement |
| Wierenga et al. 2009 | <https://doi.org/10.1017/S1355617709090468> | 20 | 4 | Tools > vehicles > animals; overt naming from greyscale photographs |
| Bracci et al. 2012 | <https://doi.org/10.1152/jn.00619.2011> | 14 | 1 | Tools > animals; 1-back task on greyscale photos |
| Perani et al. 1999 | <https://doi.org/10.1016/S0028-3932(98)00073-6> | 11 | 1 | Tools > animals; same/different judgement on line drawings |
| Roberts & Humphrey 2010 | <https://doi.org/10.1016/j.neuroimage.2010.05.044> | 12 | 3 | Tools > natural scenes; category similarity judgement |
| Vingerhoets et al. 2011 | <https://doi.org/10.1002/hbm.21078> | 16 | 2 | Tool > non-tool movements; planning pantomime actions |
| Vingerhoets et al. 2013 | <https://doi.org/10.1016/j.cortex.2011.11.003> | 10 | 1 | Tool > control object (egg); pantomiming actions |
| Cappa et al. 1998 | <https://doi.org/10.1006/nimg.1998.0368> | 13 | 2 | Tools > animals; familiarity/use judgements |
| Grossman et al. 2013 | <https://doi.org/10.1016/j.neuroimage.2012.11.057> | 18 | 3 | Manmade > natural objects; same/different judgement (words) |
| Lewis et al. 2005 | <https://doi.org/10.1523/JNEUROSCI.0419-05.2005> | 20 | 5 | Tools > animals; covert category naming (auditory clips) |
| Mahon et al. 2007 | <https://doi.org/10.1016/j.neuron.2007.07.011> | 17 | 2 | Tools > animals; naming of greyscale photos |
| Mahon et al. 2010 | [https://doi.org/10.1177/0956797610370754](https://doi.org/10.1177%2F0956797610370754) | 20 | 1 | Tools > non-tools; size judgement (spoken words) |
| Kroliczak et al. 2009 | <https://doi.org/10.1093/cercor/bhn261> | 12 | 2 | Tools > “mental” verbs; pantomiming movements |
| Johnson-Frey et al. 2005 | <https://doi.org/10.1093/cercor/bhh169> | 24 | 3 | Tools > scrambled tools; planning use gestures |
| Kassuba et al. 2011 | <https://doi.org/10.1016/j.neuroimage.2011.02.032> | 19 | 5 | Object > texture stimuli; matching |
| Roberts & Humphreys 2010 | <https://doi.org/10.1016/j.neuroimage.2010.05.044> | 14 | 3 | Objects > scenes; category matching |
